# Computational prediction of a multi-epitope Human Metapneumovirus vaccine candidate through integrated reverse vaccinology and pan-genomic approaches

**DOI:** 10.1371/journal.pone.0357758

**Published:** 2026-09-15

**Authors:** Nazia Fairooz Alam, Moumita Chakrabarty, Tanmoy Debnath, Mohammad Ashik Sheikh, Md Imtiaz, Sadia Jannat Tauhida, Md Nazmul Hasan, Md Mohaimenul Islam Tareq

**Affiliations:** 1 Analytical Research and Development, The ACME Laboratories Ltd, Dhulivita, Dhamrai, Dhaka, Bangladesh; 2 Microbiology Program, Department of Mathematics and Natural Sciences, BRAC University, Dhaka, Bangladesh; 3 One Health Laboratory, International Centre for Diarrheal Diseases Research, Bangladesh (icddr, b), Dhaka, Bangladesh; 4 Laboratory of Pharmaceutical Biotechnology and Bioinformatics, Jashore University of Science and Technology, Jashore, Bangladesh; 5 Infinity Research and Innovation Institute, Dhaka, Bangladesh; Cairo University Faculty of Veterinary Medicine, EGYPT

## Abstract

Human metapneumovirus (HMPV) is a primary cause of global respiratory infections yet no approved vaccine currently exists. This study computationally predicts a multi-epitope vaccine candidate using a diverse dataset of 65 HMPV sequences spanning five continents. Following the screening of lead proteins for antigenicity and virulence, fifteen highly conserved MHC-I, MHC-II and B-cell epitopes were prioritized. These were integrated with a putative L7/L12 adjuvant using optimized AAY, GPGPG, and KK linkers to design three constructs (HMPV_V1–V3). Structural validation identified HMPV-V2 as the lead candidate that exhibits a Z-score of-5.24 and 87.7% of residues in favored Ramachandran regions indicating excellent stereochemical quality and structural stability. In silico docking indicated a strong predicted binding affinity between HMPV-V2 and the TLR4 receptor (energy: −969.2). Immune simulations predicted a robust adaptive response characterized by high IgG1 titers, memory B-cell maturation, and a Th1-dominant cytokine profile. Furthermore, molecular dynamics simulations suggested exceptional structural integrity for HMPV-V2, maintaining a low RMSD of 8.213 and RMSF of 0.737 throughout the simulation. Optimized in silico cloning into the pET28a (+) vector indicated a high potential for protein expression in E. coli systems. While these findings provide a theoretically grounded blueprint for vaccine development, this study is entirely computational and lacks experimental validation. Further in vitro and in vivo testing is required to confirm the actual safety and immunogenicity of the proposed candidate.

## Introduction

Human metapneumovirus (HMPV) belongs to the *Pneumoviridae* family. It was first detected in the Netherlands in children afflicted with acute respiratory infections in 2001 [1]. It is retrospectively assumed that HMPV was circulating undetected for a long period of time, which supports its noteworthy public health significance [2]. HMPV is now widely recognized as a leading etiologic agent of respiratory tract infections globally. It causes infections in infants, young children, older adults, and immunocompromised individuals [3,4]. HMPV infections manifest as several forms, such as rhinorrhea, cough, and low-grade fever, to severe lower respiratory tract diseases [3,5].

HMPV infections are found to be very similar to respiratory syncytial virus (RSV) infections, exhibiting seasonal peaks particularly in winter and early spring in temperate regions [6]. Documentation of hospitalization rates reveal that over 600,000 children under five years of age undergo hospital admissions annually [7]. Adults aged ≥ 65 years fall into a very vulnerable group as they miserably suffer from severe respiratory illness, extended hospital stays, and high mortality [8,9]. The burden shows a more severe shape in low- and middle-income countries owing to limited diagnostic infrastructure and co-infections with other respiratory microorganisms [9].

HMPV has two major genetic lineages, A and B. These lineages are subdivided into subgroups A1, A2, B1, and B2 [10]. They often co-circulate, leading to swift shifts in predominance. Genetic variation is the cause of differences in antigenicity, immune evasion, and disease severity [11,12].. Viral surface proteins, namely fusion protein (F) and glycoprotein (G), play the most critical role in ensuring host cell entry and immune modulation. G protein has been mostly observed to mediate interference with host interferon signaling which significantly aids viral pathogenesis and persistence [13].

HMPV is usually transmitted through respiratory droplets and close contact with infected individuals. People with infections shed the virus in nasal and throat secretions for prolonged periods [12,14]. Indirect transmission takes place via contaminated surfaces or fomites, although with less efficiency [15]. Outbreaks are common in households, childcare centers, long-term care facilities, and hospitals, mostly in crowded population settings [16]. Viral stability and shedding in the environment are the reasons for the complexities in infection control [17].

Clinical severity is largely dependent on multiple factors, such as host age, immunological status, comorbidities, prior exposure to HMPV or other related respiratory viruses [15,18]. Older adults with chronic illnesses and infants under 12 months of age are highly prone to be infected with severe disease. In particular, immunocompromised individuals are greatly predisposed to prolonged infections and subsequent complications [19]. Clinical outcomes can be aggravated due to co-infection with RSV, influenza, or any other bacterial pathogens.

The host immune response to HMPV relates to both innate and adaptive immune mechanisms. Innate immunity is triggered by the recognition of viral RNA through pattern recognition receptors, leading to the generation of type I and III interferon responses [20]. Adaptive immunity not only includes neutralizing antibodies targeting the F and G proteins, but also T-cell responses that contribute to viral clearance [21]. However, HMPV can cause recurrent infections and incomplete immunity by suppressing immune signaling and evading host defenses [11,20]. Conspicuous comprehension about these immune mechanisms is very crucial to develop effective vaccines and therapeutics.

At present, no licensed vaccine or specific antiviral therapy is available for HMPV. Vaccine development attempts have already explored live-attenuated, inactivated, recombinant vector, and subunit approaches [22–24]. Live-attenuated vaccines show striking prospects in inducing protective immunity in preclinical models. But this approach raises severe safety concerns. Subunit or inactivated vaccines can ensure improved safety profiles but often require adjuvants to provoke robust immune responses [24]. Expanded cross-lineage protection crops up as a major challenge because of the genetic diversity of circulating HMPV strains [25].

Suitable animal models, namely cotton rats, hamsters, and nonhuman primates, are proven to be essential to evaluate vaccine candidates. These models have the potency to generate relevant data which can provide critical preclinical evidence for vaccine development. Recent advances in reverse vaccinology and immunoinformatic are commendable which have enabled the rational design of multi-epitope subunit vaccines. Therefore, they are successful in predicting conserved B- and T-cell epitopes across HMPV lineages [26–28]. These attempts permit the *in silico* evaluation of antigenicity, allergenicity, population coverage, and structural stability, consequently minimizing the risks associated with live-virus vaccines [29–31].

Despite these advancements, significant unmet gaps remain in HMPV vaccine research, particularly in developing broadly protective and safe vaccines that can overcome the challenges posed by viral genetic diversity and immune evasion mechanisms. Previous studies have explored various vaccine strategies, but often face limitations in achieving comprehensive cross-lineage protection or ensuring optimal immunogenicity without adverse effects [32–36]. Specifically, a key challenge lies in identifying highly conserved and immunogenic epitopes that can elicit both robust humoral and cellular immune responses against the diverse HMPV strains circulating globally, while simultaneously ensuring the vaccine’s safety and stability.

In the conducted study, we successfully ensured relevant applications of reverse vaccinology and immunoinformatic to design a multi-epitope vaccine that can target the conserved antigenic regions of HMPV. Unlike previous approaches that may focus on a limited set of antigens or lack a comprehensive pan-genomic perspective, our integrated strategy leverages pan-genomic analysis to identify highly conserved proteins across a wide range of HMPV strains, thereby enhancing the potential for broad-spectrum protection.

After the proper identification of overlapping B- and T-cell epitopes from the major circulating lineages, we aimed at eliciting both humoral and cellular immunity against the virus. The concerned vaccine constructs underwent further evaluation for their immunogenic potential and binding affinity to immune receptors exploiting computational approaches. The strategy lays out a promising framework for developing safe and broad-spectrum HMPV vaccines. At the same time, it acknowledges the urgent need for prophylactic interventions against HMPV [18].

## Materials and methods

### Retrieval of protein, re-annotation and pan-genome analysis

A collection of 65 HMPV genome sequences was retrieved from the NCBI Virus database (https://www.ncbi.nlm.nih.gov/labs/virus), a well-established and publicly available platform that provides coordinated genomic and proteomic information for a broad spectrum of viral species. The HMPV sequence dataset included strains from multiple geographic regions (Africa, Asia, Europe, North America, and South America). This wide geographical coverage accounts for regional variations in viral evolution. The dataset covers a comprehensive 5-year period from January 1, 2020, to May 16, 2025. This timeframe captures the most recent evolutionary trends of HMPV.. Complete HMPV genome sequences were downloaded from the NCBI Virus database in FASTA format and re-annotated using PROKKA (version 1.14.6), which can be applied to bacterial, archaeal and selected viral genomes with an emphasis on coding sequence prediction [37]. The resulting GFF3 files were evaluated using Roary (version 3.13.0) [38] to cluster homologous coding sequences and identify conserved (core) and accessory genes across the HMPV genomes. Roary was employed as a gene clustering framework based on the Prokka-generated annotations, utilizing the PRANK alignment algorithm (v.170427) for homologous gene comparison [39]. Although Roary was originally developed for bacterial pan-genome analyses, it was applied here to identify conserved coding sequences for downstream reverse vaccinology and comparative genomic analyses. Subcellular localization of the core genes was evaluated using Virus-mPLoc (http://csbio.sjtu.edu.cn/bioinf/virus-multi), a recently introduced fusion-based classifier [40].. Understanding the subcellular location of viral proteins is crucial for developing effective antiviral medicines.

### Prediction of T-cell and B-cell epitopes

Surface and extracellular proteins of pathogens are considered promising candidates for chimeric vaccine development due to their immunogenic potential. T-cell epitopes, typically obtained from protein fragments of pathogenic cells, are presented on the cell surface by MHC molecules. Cytotoxic T lymphocytes (CD8 ⁺ T cells) recognize peptides bound to MHC class I, whereas helper T lymphocytes (CD4 ⁺ T cells) recognize peptides presented by MHC class II molecules. T-cell epitopes are generally more restricted than B-cell epitopes, which are frequently linked to exposed and flexible antigen surface regions. For prediction, the Immune Epitope Database (https://www.iedb.org/) was utilized. MHC class I and II epitopes have been detected using the stabilized matrix method (SMM) and neural network-prediction tools (netMHCpan-4.1 EL for MHC I and netMHCIIpan-4.1 EL for MHC II) [41]. Epitopes with strong binding affinity (IC50 ≤ 200 nM), overlapping positions, and lengths of 8–22 residues were prioritized. To achieve broad global applicability of the predicted epitopes, both MHC class I and MHC class II alleles were selected to represent major HLA distributions across global populations. The selected alleles collectively represent diverse ethnic groups like European, East and South Asian, African and West Asian populations. Although these alleles collectively provide broad global population representation, certain populations such as Indigenous groups from Oceania, Native American populations, and some Central African populations may be relatively underrepresented due to the presence of other region-specific HLA alleles that were not included in this analysis. The computational analysis performed in this study assumes comparable HLA expression and antigen presentation efficiency across alleles, which is a common assumption in immunoinformatic-based epitope prediction studies. The primary objective of this approach is to identify potential epitopes with predicted binding affinity to widely distributed HLA molecules rather than to model individual immune responses in vivo. B-cell epitope prediction was conducted using the BCpred server (https://webs.iiitd.edu.in/raghava/bcepred/). BCpred identifies linear B-cell epitopes that stimulate immunological responses and produce antibodies with a threshold value of 0.8 and 75% Specificity under default parameters [42].

### Selection of epitopes and vaccine design

Both T-cell and B-cell epitopes were incorporated in the multi-epitope vaccine construct to promote robust and effective immunogenicity. Candidate epitopes predicted by IEDB and BCpred were further screened for antigenicity and toxicity using AllerTOP v2.0 (https://www.ddg-pharmfac.net/allertop_test/), Vaxijen v2.0 (https://www.ddg-pharmfac.net/vaxijen/VaxiJen/VaxiJen.html), and ToxinPred (https://webs.iiitd.edu.in/raghava/toxinpred2/batch.html). The VaxiJen server applies auto-cross-covariance transformation to classify proteins as antigens based on their major amino acid characteristics [43]. Allertop v2.0 employs a collection of innovative models for allergy prediction using auto- and cross-covariance transformation, along with multiple machine learning techniques for classification approaches [44]. The ToxinPred program discovers toxic segments in provided peptides using the Support Vector Machine as the prediction approach [45]. Only epitopes predicted as antigenic, non-allergenic, and non-toxic were retained. For immune potentiation, 50S ribosomal protein L7/L12 was selected as an adjuvant because it has been reported to enhance immune responses by stimulating innate immune pathways and improving antigen presentation in multi-epitope vaccine designs [46]. It has been attached to the N-terminus of the construct via an EAAAK linker which prevents domain interference, maintains higher structural stability than flexible linkers and maximizes the multiple functions of fusion proteins to enhance immunogenicity [47].

Linkers in multiepitope vaccines provide appropriate spacing between epitopes, maintaining structural integrity and preventing interference. Additional linkers were employed to assemble the epitopes. The GPGPG linker was used to join helper T-cell (HTL) epitopes because it helps prevent junctional epitope formation and supports efficient antigen processing and presentation through the MHC class II pathway [48]. The AAY linker was used for cytotoxic T-lymphocyte (CTL) epitopes since it contains proteasomal cleavage sites that facilitate proper epitope processing and presentation via the MHC class I pathway [49]. The KK linker is specifically sensitive to lysosomal proteases, facilitating the presentation of B-cell epitopes for antibody production [50]. By ordering the linkers AAY → GPGPG → KK, the vaccine mimics the natural hierarchy of antigen presentation: starting with the cytoplasmic “chopping” for cellular killers (CTL), followed by the flexible presentation for helpers (HTL), and ending with the endosomal release for antibody production (B-cells). Together, these linker strategies ensure appropriate spacing, structural stability and efficient immune recognition within the multi-epitope vaccine construct. The selected linkers (AAY, GPGPG, and KK) were chosen because they are among the most extensively validated linkers in multi-epitope vaccine design and have been widely reported to facilitate appropriate epitope processing and presentation while minimizing junctional epitope formation. Likewise, the 50S ribosomal protein L7/L12 was selected as the adjuvant due to its well-documented immunostimulatory properties and ability to enhance both innate and adaptive immune responses whereas the rigid EAAAK linker was employed to provide structural separation between the adjuvant and the epitope region and minimize steric interference. The final construct was chosen based on favorable predicted immunological and physicochemical properties predicted through immunoinformatic analyses, which is a commonly adopted approach in similar vaccine design studies. Investigation of alternative construct configurations may be considered in future studies

### Immunological and physicochemical profiling

Evaluation of the physiological characteristics of the vaccine constructs is one of the most important steps in the development process to guarantee it is safe, effective, and able to function properly in the human body. The physicochemical features of the designed vaccine construct were assessed using the ExPASy ProtParam server (https://web.expasy.org/protparam/) [51]. Parameters such as molecular weight, theoretical isoelectric point, instability index, and half-life were assessed to evaluate its stability and suitability for expression. The Protein-Sol web server predicts protein solubility using experimentally determined data from *Escherichia coli* proteins expressed in a cell-free system. The prediction relies on the observed bimodal distribution of protein solubility where solubility is defined as the proportion of a protein remaining in the soluble fraction after centrifugation relative to the total protein produced, rather than as a thermodynamic measure of solubility [52]. Immunological features, including antigenicity, allergenicity, and toxicity, were also rechecked to confirm that the construct is safe and can work well with the host immune system.

### Homology-based modeling, tertiary structure prediction, and validation

After selecting the multi-epitope vaccine constructs based on their physicochemical and immunological compatibility, they were then modeled and structurally validated for further analysis. Three-dimensional (3D) models of the vaccine construct were generated using the GalaxyWEB server, followed by refinement with Galaxy Refine 2 [53]. Structural quality was evaluated using ERRAT and PROCHECK, available within the SAVES v6.0 server (https://saves.mbi.ucla.edu/), as well as ProSA-Web (https://prosa.services.came.sbg.ac.at/prosa.php) [54]. These tools helped to confirm the quality of each model, and the design with the best overall structural performance was chosen for further analysis.

### Molecular docking

Molecular docking experiments help identify binding connections between targeted ligands and proteins [55]. TLR4 plays a key role in activating the innate immune response to hMPV [56]. We chose TLR4 as the receptor and used the vaccine construct as the ligand. In the present study, docking analysis was performed only to predict the potential interaction and binding affinity between the designed vaccine construct and immune receptors, which may indicate the capacity of the construct to trigger innate immune responses although experimental validation is required to confirm these predictions. The docking study was conducted using ClusPro v2.0 web tools (https://cluspro.bu.edu/login.php) [57]. ClusPro is more accurate than previous methods for docking unbound protein structures [58]. The web tools use a process that combines energy minimization and clustering of the lowest-energy models to perform compact bond docking, which docks the protein-ligand complex. The best-docked complex was chosen based on the center along with a low energy score and cluster members [59]. Molecular docking provides a computational prediction of potential binding interactions and affinities, rather than a direct validation of biological function. While favorable docking scores suggest a strong potential for receptor engagement, these in silico findings require experimental validation to confirm actual immune activation. For this study, a direct comparison against known ligand-receptor complexes was not performed, but the methodology aligns with established practices for initial screening of vaccine candidates.

### Disulfide engineering of vaccine constructs

Disulfide engineering is an effective protein engineering approach used to enhance protein stability and modify functional properties. Computational prediction tools facilitate this process by identifying amino acid residue pairs that are likely to form stable disulfide bonds after mutation to cysteine residues [60]. To improve the structural stability of the vaccine constructs, disulfide engineering was performed for the refined three-dimensional models of all three vaccine constructs using Disulfide by Design 2 (DbD2). Potential residue pairs for cysteine substitution were identified based on geometric parameters including χ3 dihedral angle and bond energy. Residue pairs with bond energies below 2.2 kcal/mol and favorable geometric constraints were considered suitable for disulfide bond engineering. Native cysteine pairs were excluded from further analysis and only newly identified residue pairs were selected for cysteine substitution.

### Codon optimization and *in-silico* cloning procedure

To improve vaccine stability, disulfide bonds were attached to the model. The Disulfide by Design 2.12 web server was used to apply disulfide engineering, a cutting-edge technique [61]. Codon optimization and silico cloning were implemented on the best selected vaccine structure (HMPVV_2). Codon optimization was performed with the Java Codon Adaptation (JCat) tool to improve the expression of the vaccine gene in the *E. coli* expression system [62]. JCat analyzes the codon adaptation index (CAI) and GC content to assess the expression of the cloned sequence. For optimal transcription and translation efficiency, aim for a GC content of 30–70% and CAI value of 1 in the modified sequence [63]. The last step was to use Snap Gene (https://www.snapgene.com/) to virtually clone the most important vaccine design into the pET-28a (+) expression vector.

### Immune simulation

The C-ImmSim tool was used to conduct computerized immunological tests in order to assess the vaccine’s immunological activity [64]. This program uses a position-specific score matrix (PSSM) and several algorithms to predict and analyze epitope and immune-mediated interactions. Immune simulation following three sequential vaccine doses administered at time steps 1, 84, and 168 corresponding to four-week intervals between immunizations. It demonstrated robust secondary and tertiary immune responses characterized by increased antibody titers, expansion of memory B and T cells and elevated cytokine production indicating the vaccine’s potential to induce long-lasting protective immunity. For the simulation, three vaccine doses were given at time steps 1, 84, and 168, representing a four-week interval between injections. The immunological simulation assessment was carried out utilizing a thousand simulation cycles, although the overall simulation measurement setting was initially 10 cycles.

### Molecular dynamics simulation

A molecular dynamics computational technique was used to investigate the molecular behavior of the epitope and its stability in protein-protein interactions. Vaccines can be present as monomers or M-dimers, with M-dimers forming before signal transduction begins. In this study, we assessed the impact of glycosylation and the thermodynamic stability of the designed epitope vaccine using three docked vaccine complexes: HMPVV_1, HMPVV_2, and HMPVV_3. Three vaccine-receptor complexes were selected for 100 ns Molecular Dynamics (MD) simulations to evaluate the thermodynamic stability of the protein-ligand complexes. The Schrödinger “Desmond v3.6 Program” (Paid version) was used on the Linux platform to perform the molecular dynamics simulations that assessed various protein-ligand complex architectures. The TIP3P aqueous model is used in the provided approach to produce a desired volume under periodic boundary conditions. An orthorhombic structure with a division of 10 Å is included in this condition. To neutralize the system’s electrical charge, necessary ions, including 0+ and 0.15 M salt (Na+ and Cl−), were added and evenly distributed within the solvent environment during the simulation setup. A variety of agonist combinations were used to create the solvated protein structures. The system framework was then refined and became competent at utilizing OPLS3e, a protocol that makes use of force field constants and is part of the Desmond package. The NPT simulations were operated at 300 K and 1.01325 bar pressure with global Nose-Hoover temperature pairings and an isotropic method. The system included 50 ps equilibration steps with a restraint force of 1.2 kcal/mol. The Simulation Interaction Diagram (SID) from the Desmond modules of the Schrödinger Suite evaluated the quality of the molecular dynamics simulation. The images of Molecular Dynamics Simulations were generated using the Schrödinger Maestro program version 9.5. The possible simulation scenario and molecular dynamics simulation accuracy were measured through the Simulations Interaction Diagram (SID) of the Desmond modules of the Schrödinger software. We evaluated the Secondary structural element (SSE), root-mean-square deviation (RMSD), and root-mean-square fluctuation (RMSF) of vaccine-receptor complex structures using work trajectory data to evaluate their stability.

### Autoimmunity risk assessment

To evaluate potential cross-reactivity and autoimmunity risks, a comprehensive Protein BLAST (blastp) analysis of our selected HMPV vaccine candidate protein (HMPV_V2) was performed against the *Homo sapiens* protein sequences within the NCBI nr (non-redundant) database. Stringent search parameters were employed, including an E-value threshold of 0.05, the BLOSUM62 matrix, and enabled low-complexity filtering. The absence of statistically significant alignments, high-identity hits, or extended alignment regions between HMPV_V2 and human proteins was used to infer a low risk of autoimmunity arising from linear molecular mimicry.

## Results

### Construction of the pan-genome and identification of core proteins

**Fig 1:**
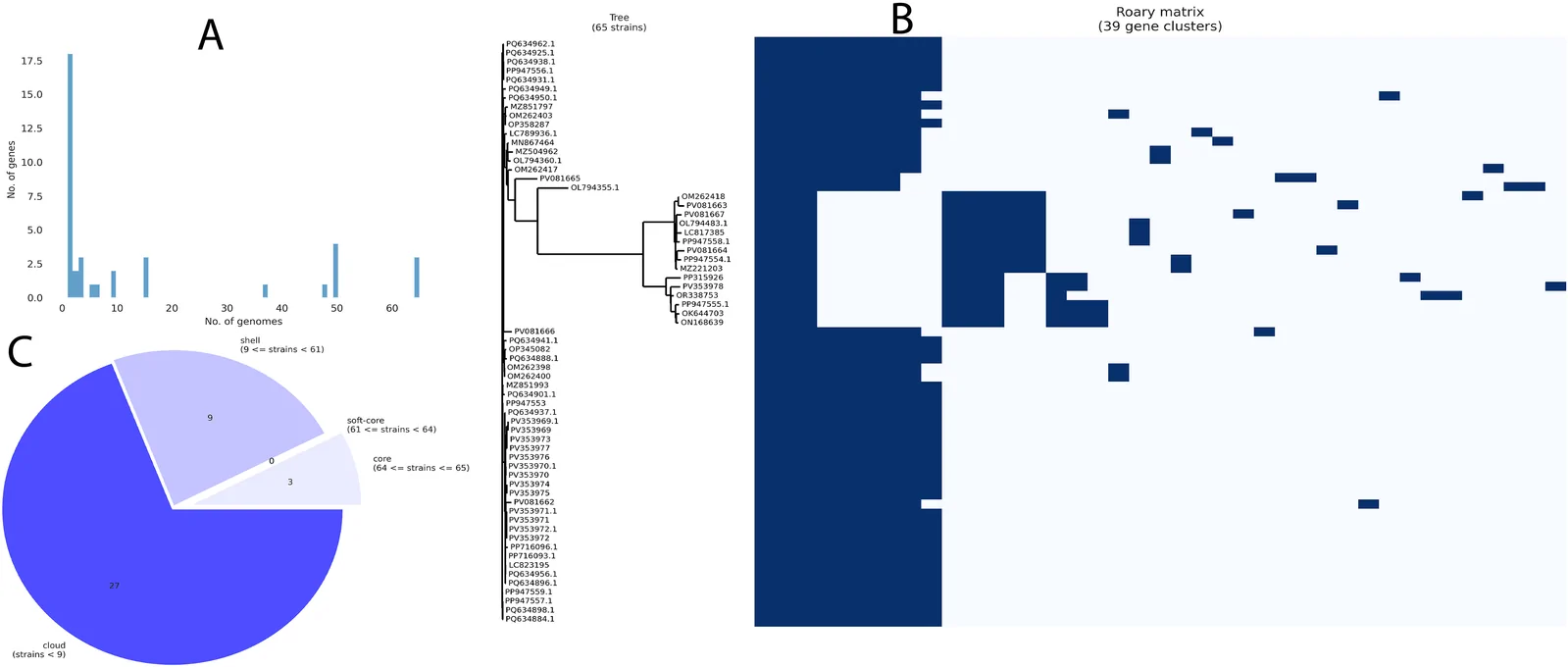
Pan-genome analysis. a) The number of genomes vs the total number of genes. b) Clustering of the genes. c) A pie chart represents several core, soft, shell, and cloud genes.

The entire pan-genome of the **65 Human Metapneumoviruses** comprised of **39 genes**. Among them, **3 genes** were selected as core genes, since they were found to be present in all HMPV genomes with a minimum similarity threshold of 90%. Fig 1a and 1b display a matrix that represents the presence and absence of core and auxiliary genes, together with a full genome phylogenetic tree. The examination of the whole pan-genome showed that there were **3 essential genes (7.69%)** present in all isolates, **9 genes (23.08%)** that were present in a subset of isolates, and **27 genes (69.23%)** that were found in fewer than 9 samples (Fig 1c). **3 core genes** underwent the vaccine target identification process that met a cut-off value of 100%. Based on antigenicity, immunogenicity, and subcellular localization, these three proteins were selected as vaccine targets: **Nucleoprotein (N; NCBI accession: LC789936.1_00001)**, **Matrix protein (M; NCBI accession: LC789936.1_00003)** and **M2-1 protein (transcription antitermination factor) (NCBI accession: LC789936.1_00005)**. The nucleoprotein (N) encapsulates the viral RNA genome and plays a crucial role in viral transcription and replication. The matrix protein (M) is responsible for virion assembly, structural organization, and viral budding. Matrix protein M2-1 functions as a transcription antitermination factor, facilitating efficient viral RNA transcription and gene expression by preventing premature termination during mRNA synthesis. Owing to their conserved nature and essential roles in the HMPV life cycle, these proteins were selected as targets for the design of multi-epitope vaccine constructs.

Furthermore, proteins located on the outer membrane were selected for further study by predicting their subcellular localization using Virus-mPLoc.

### Identification of immunodominant epitopes from antigenic proteins

The top antigenic proteins that do not induce allergic reactions or harmful consequences were carefully selected. Epitopes exhibiting antigenicity scores greater than 0.4 were chosen for further analysis. Following that, these proteins were thoroughly examined to identify potential lead epitopes for the development of a chimeric vaccine against HMPV. The epitopes were thoroughly tested for antigenicity, allergenicity, and toxicity and (Table 1) three vaccine models were developed utilizing the epitopes to guarantee the most effective vaccine. By concurrently targeting several antigenic epitopes, the design of a multi-epitope vaccination seeks to elicit a more robust and extensive immune response against a virus. This technique aims to elicit a more comprehensive immune response and stimulate the development of diverse immune cells, such as antibodies and T cells. It was demonstrated that the acquired epitopes were conserved throughout several HMPV strains. More protection against different HMPV strains is promoted by using conserved epitopes in a multi-epitope vaccine. The conservation of assigned epitopes is seen in Table 2. The conservation analysis of the predicted epitopes revealed moderate to superior conservation among the three selected HMPV proteins. This high level of conservation suggests that epitopes may provide broader strain coverage and are less likely to be affected by viral genetic variability. Nevertheless, the relatively lower conservation observed for certain epitopes suggests that the final vaccine design may benefit from combining epitopes from multiple proteins to improve coverage against diverse viral variants. Nevertheless, it should also be noted that the conservation analysis was performed using the available sequence dataset and therefore the results reflect the diversity present in the analyzed sequences rather than the complete global genetic diversity of HMPV.

**Table 1:** Epitope prediction for the target protein. The leading MHC-I and MHC-II T-cell and B-cell epitopes, together with the immunogenic characteristics that correspond to them.

Protein ID	MHC-I Epitopes	Score	Antigenicity Score	MHC-II Epitopes	Score	Antigenicity Score	B-Cell Epitopes	Score	Antigenicity Score	Antigenicity	Allergenicity	Toxicity
HMPV_V1	HFLNVSDDSQNDY	0.831	0.9653	EIGIQYISTALGSER	0.5667	1.1205	LRQSPKAGLL	0.5305	0.996	Probable Antigen (0.4629)	Probable Non-Allergen	Non-Toxin
	LNVSDDSQNDY	0.923	0.664	QEITLLCGEILYAKH	0.2491	0.7867	VSVQAELKQVTE	0.985	0.845			
	FLNVSDDSQNDY	0.937	0.5694	QQEITLLCGEILYAK	0.5971	0.7314	AYGAGQTMLRWG	0.881	0.7933			
	NVSDDSQNDY	0.936	0.5255	QKVYYRSLFIEYGKA	0.2121	0.6238	YAKSLKESNKIN	0.429	0.6608			
	QAELKQVTEVY	0.401	0.5014	VYYRSLFIEYGKALG	0.3422	0.5993	ETTVRRANRVLS	0.43	0.5472			
HMPV_V2	KTLTITTLY	0.935	0.5848	LTITTLYAASQSGPILKV	0.8902	0.5135	KNTPVTIPAFIKSVSIKESE	0.961	0.5667	Probable Antigen (0.5191)	Probable Non-Allergen	Non-Toxin
	AAMSVLPKR	0.771	1.1809	KSVSIKESESATVEAA	0.8776	0.8353	QLKTLTITTLYAASQSGP	0.851	0.4634			
	DTYQGIPYTAAV	0.487	0.6013	TIPAFIKSVSIKESES	0.8693	0.4423	CEVKTVYLTTMKPY	0.886	0.5053			
	TVCEVKTVY	0.527	0.638	IKSVSIKESESATVEAAI	0.7491	0.7872	DTYQGIPYTAAVQV	0.802	0.5775			
	SVLPKRFEV	0.900	0.8973	SKFVSSAKSVGKKTHD	0.5344	0.4992	KVNASAQGAAMSVLPKRF	0.667	0.5295			
HMPV_V3	LGSTNVVQGY	0.128	0.759	QEVEVRQARDNKLSD	0.1871	0.7925	GAGREDRTQDFVLG	0.828	1.2766	Probable Antigen (0.5190)	Probable Non-Allergen	Non-Toxin
	IIDLSAGADNDSSY	0.340	0.7109	EVEVRQARDNKLSDS	0.2643	0.7712	MSRKAPCKYEVRGK	0.789	1.0693			
	VLGSTNVVQGY	0.081	0.5812	SSYALQDGESTNQVQ	0.4757	0.7433	GAGREDRTQDFV	0.676	0.9634			
	STNVVQGY	0.256	0.5191	DSSYALQDGESTNQV	0.8009	0.7227	QLQEVEVRQARDNK	0.981	0.7139			
	LSAGADNDSSY	0.494	0.4492	NDSSYALQDGESTNQ	0.7541	0.7206	NRGSECKFNHNYWSWPDR	0.772	0.6684			

**Table 2:** Comparative conservation analysis. The epitope conservancy of the selected B- and T-cell epitopes was assessed using IEDB.

Protein	MHC-I Epitopes	Conservation	MHC-II Epitopes	Conservation	B-Cell Epitopes	Conservation
HMPV_V1	HFLNVSDDSQNDY	6.09% (54/886)	EIGIQYISTALGSER	14.11% (125/886)	LRQSPKAGLL	20.54% (182/886)
	LNVSDDSQNDY	6.09% (54/886)	QEITLLCGEILYAKH	16.59% (147/886)	VSVQAELKQVTEL	0.00% (0/886)
	FLNVSDDSQNDY	6.09% (54/886)	QQEITLLCGEILYAK	16.48% (146/886)	AYGAGQTMLRWG	47.18% (418/886)
	NVSDDSQNDY	6.09% (54/886)	QKVYYRSLFIEYGKA	47.97% (425/886)	YAKSLKESNKIN	10.84% (96/886)
	QAELKQVTEVY	32.96% (292/886)	VYYRSLFIEYGKALG	47.18% (418/886)	ETTVRRANRVLS	49.10% (435/886)
HMPV_V2	KTLTITTLY	13.53% (23/170)	LTITTLYAASQSGPILKV	24.71% (42/170)	KNTPVTIPAFIKSVSIKESE	32.94% (56/170)
	AAMSVLPKR	13.53% (23/170)	KSVSIKESESATVEAA	51.18% (87/170)	QLKTLTITTLYAASQSGP	25.88% (44/170)
	DTYQGIPYTAAV	34.12% (58/170)	TIPAFIKSVSIKESES	52.35% (89/170)	CEVKTVYLTTMKPY	43.53% (74/170)
	TVCEVKTVY	50.59% (86/170)	SKFVSSAKSVG	70.59% (120/170)	DTYQGIPYTAAVQV	34.12% (58/170)
	SVLPKRFEV	13.53% (23/170)	IKSVSIKESESATVEAAI	43.53% (74/170)	KVNASAQGAAMSVLPKRF	12.94% (22/170)
HMPV_V3	LGSTNVVQGY	96.00% (48/50)	QEVEVRQARDNKLSD	38.00% (19/50)	GAGREDRTQDFVLG	98.00% (49/50)
	IIDLSAGADNDSSY	34.00% (17/50)	EVEVRQARDNKLSDS	38.00% (19/50)	MSRKAPCKYEVRGK	92.00% (46/50)
	VLGSTNVVQGY	96.00% (48/50)	SSYALQDGESTNQVQ	0.00% (0/50)	GAGREDRTQDFV	98.00% (49/50)
	STNVVQGY	96.00% (48/50)	DSSYALQDGESTNQV	0.00% (0/50)	NRGSECKFNHNYWSWPDR	76.00% (38/50)
	LSAGADNDSSY	38.00% (19/50)	NDSSYALQDGESTNQ	0.00% (0/50)	QLQEVEVRQARDNK	46.00% (23/50)

Furthermore, the selected core proteins (Protein 1, Protein 2, and Protein 3) are known to mediate critical molecular functions in HMPV pathogenesis, such as viral replication, host cell attachment, and immune evasion. By targeting immunodominant epitopes from these essential proteins, the proposed vaccine is computationally designed to potentially hamper these vital viral functions, thereby disrupting the HMPV life cycle and mitigating disease.

### Vaccine construction

**Fig 2:**
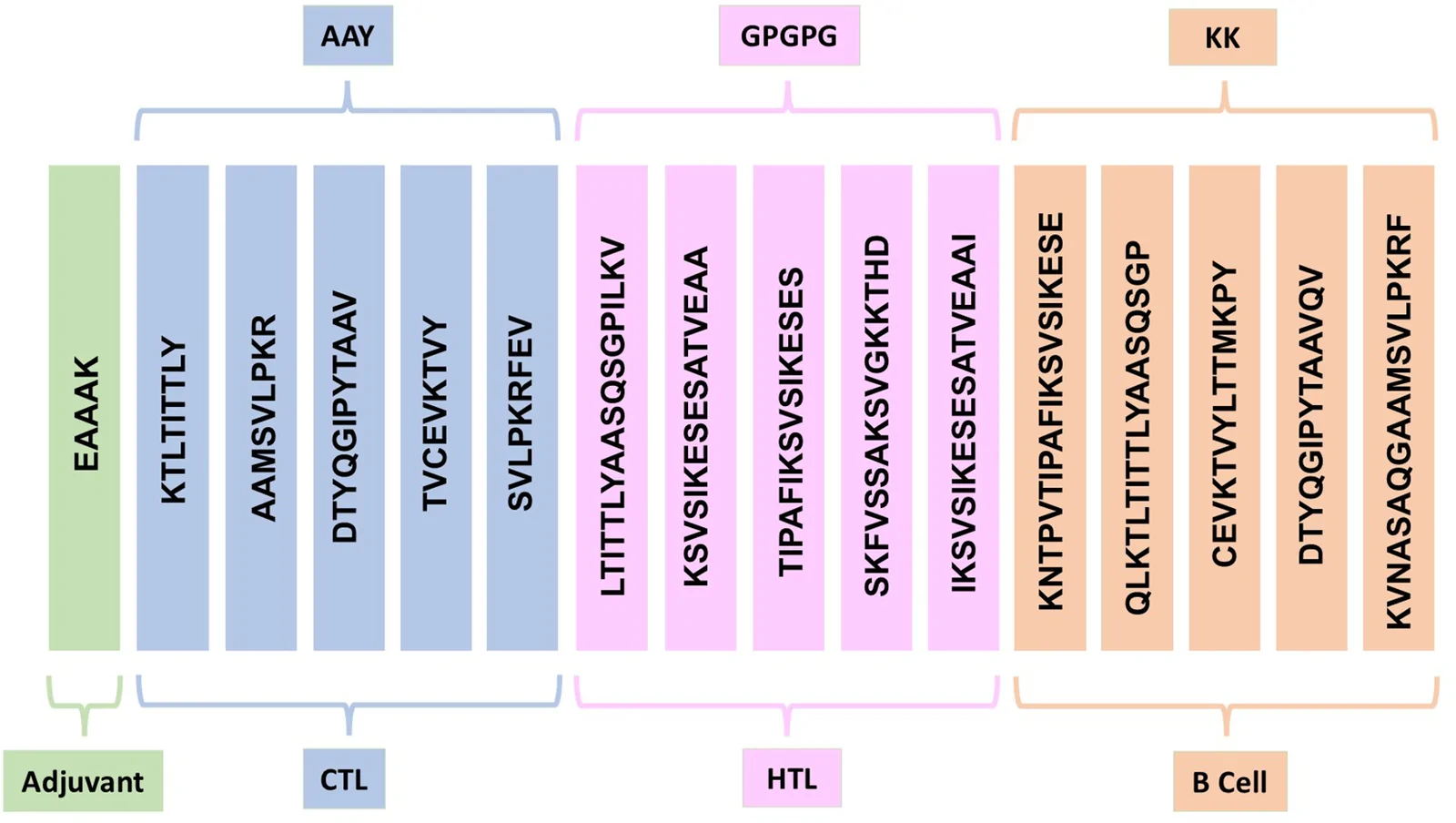
Vaccine construction. Schematic representation of the HMPV_V2 multi-epitope vaccine construct showing the arrangement of selected CTL, HTL, and B-cell epitopes linked using AAY, GPGPG, and KK linkers, respectively, along with the 50S ribosomal protein L7/L12 adjuvant attached at the N-terminus via an EAAAK linker.

The overlapping and unique epitopes shortlisted from MHC class-I, MHC class-II T-cell, and linear B-cell epitopes were selected for vaccine development. Therefore, both humoral and innate immunity could be induced by the overlapping epitopes. The chosen epitopes were amalgamated using suitable linkers and an adjuvant for optimal immune stimulation in order to create a multi-epitope vaccine. Five CTL epitopes, five HTL epitopes, and five B-cell epitopes were joined using AAY, GPGPG, and KK linkers. Furthermore, the EAAAK linker which is an immunogenicity booster was adjuvanted with the 50S ribosomal protein L7/L12 at the N-terminus to form a single construct. The construct demonstrated strong theoretical antigenicity scores and broad HLA coverage (Fig 2). The complete amino acid sequence of the final multi-epitope vaccine construct (HMPV-V2), including the adjuvant, linker sequences, and all CTL, HTL, and B-cell epitopes, is provided in S3 File to facilitate reproducibility.

### Allergenicity, antigenicity and physicochemical parameters evaluation of vaccine constructs

The physicochemical properties of the three designed vaccine constructs were analyzed to assess their stability and potential suitability for further analyses (Table 3). All constructs showed antigenicity scores above the threshold value of 0.4, indicating their predicted ability to elicit immune responses. The instability index values for HMPV_V1, HMPV_V2, and HMPV_V3 were all below 40, suggesting favorable intrinsic stability according to the ProtParam algorithm under in vitro predictive conditions. However, this parameter represents a computational estimate based on amino acid composition and should not be interpreted as direct evidence of protein stability under physiological or in vivo conditions. Experimental studies are required to confirm the stability, folding behavior, and persistence of the vaccine constructs under physiological conditions. Among the constructs, HMPV_V2 demonstrated the highest antigenicity score, the lowest instability index and the highest aliphatic index indicating comparatively better stability and potential thermostability. Additionally, the negative GRAVY values observed for all constructs suggest overall hydrophilic characteristics which may favor solubility and proper interaction within the biological environment. The scaled solubility values for HMPV_V1, HMPV_V2, and HMPV_V3 were predicted using the Protein-Sol web server: **0.64**, **0.614**, and **0.80**, respectively. All three values exceeded the Protein-Sol threshold of **0.45** indicating that the constructs are likely to be soluble upon recombinant expression. These findings are consistent with the negative GRAVY values obtained from ProtParam analysis, further supporting the overall hydrophilic nature of the vaccine constructs. However, these predictions are computational and should be interpreted cautiously until confirmed through experimental studies.

**Table 3:** Physiochemical features of the vaccine constructions applying ProtParam.

Vaccine Construct	Number of Amino Acids	Molecular weight (Daltons)	Antigenicity Score	Theoretical PI	Aliphatic Index	GRAVY	Instability index	Predicted solubility (Protein-Sol)
HMPV_V1	373	40040.34	0.4629	5.36	85.9	−0.326	35.35	0.64 (Soluble)
HMPV_V2	398	41384.89	0.5191	9.21	86.63	−0.042	26.66	0.614 (Soluble)
HMPV_V3	383	40485.7	0.519	4.98	68.36	−0.625	30.32	0.80 (Soluble)

### 3D structure prediction and validation

**Fig 3:**
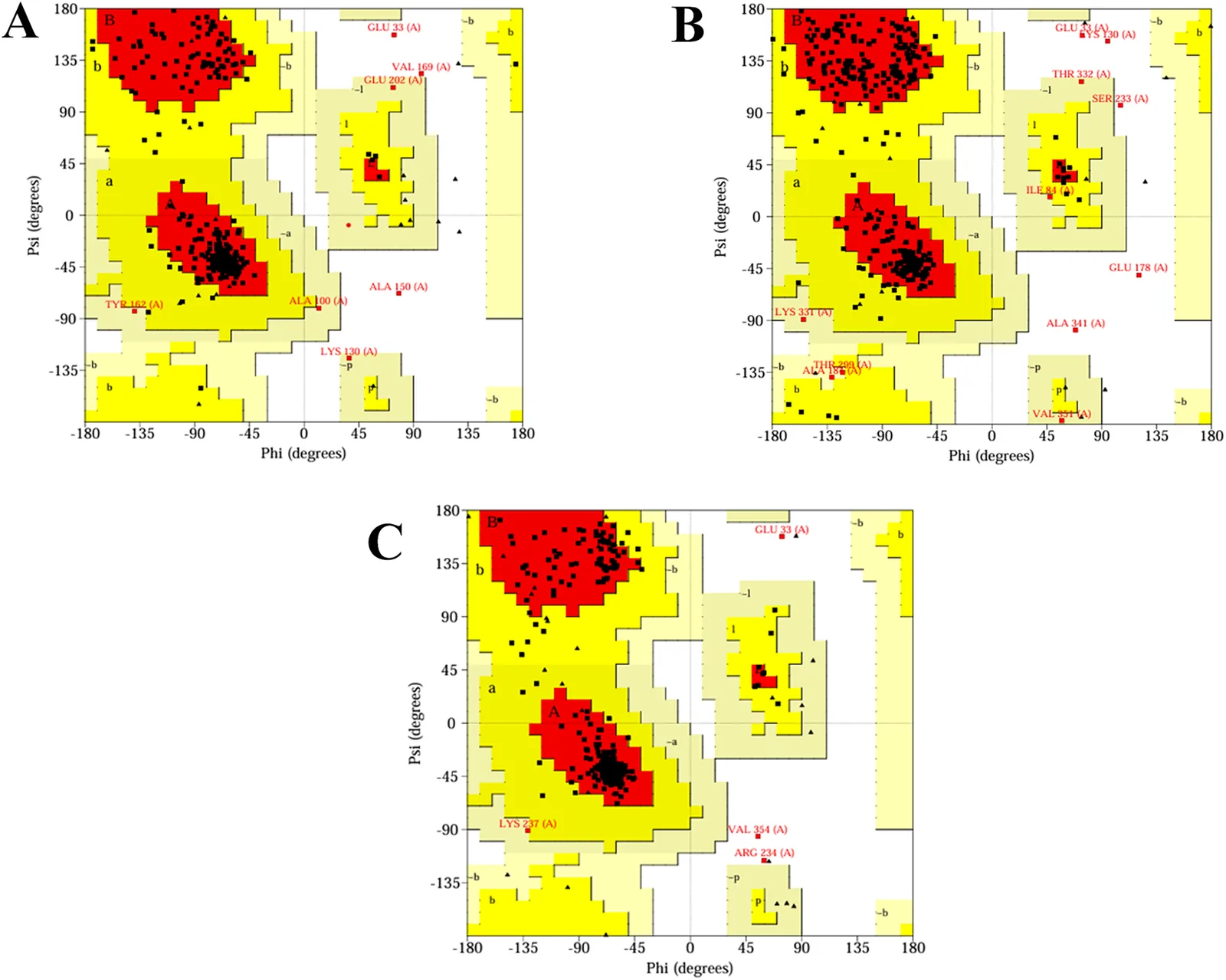
Evaluation of quality assessment for HMPV vaccines. The Ramachandran plot assessment displays **(A)**. For HMPV-V1, 89.8% of the residues are located within favored regions, 8.1% in the allowed region, and 0.9% in the disallowed region. **(B)** For HMPV-V2, 87.7% of the residues are located within favored regions, 9.1% in the allowed region, and 1.5% in the disallowed region. **(C)** For HMPV-V3, 93.5% of the residues are located within favored regions, 5.2% in the allowed region, and 0.5% in the disallowed region.

**Fig 4:**
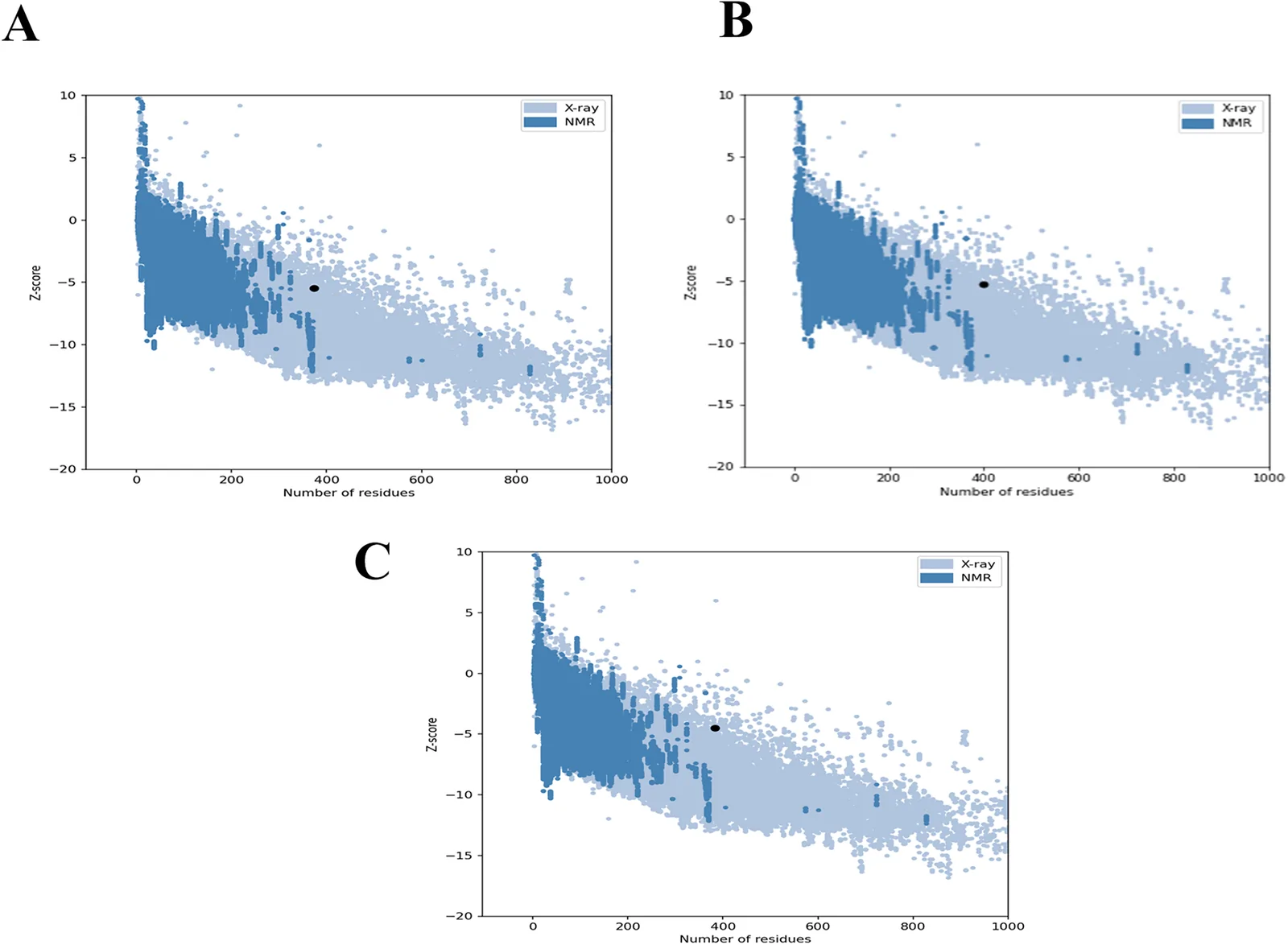
3D structure validation. According to the ProSA-web findings, the analysis of quality validation for HMPV vaccines shows a Z-score of **(A**) −5.48 for HMPV-V1. (**B**) −5.24 for HMPV-V2. (**C**) −4.51 for HMPV-V3.

The tertiary structures of the designed HMPV vaccine constructs were predicted using homology modeling. In order to investigate the molecular interactions involving the host’s immunological receptor protein, the 3D vaccine structure needs to be robust and efficient. To validate the 3D structure, the Ramachandran plots assessed the vaccine constructs’ quality. Demonstration from the Ramachandran plots plainly stated that the favored regions appeared 89.8%, 87.7% and 93.5% for HMPVV_1, HMPVV_2 and HMPVV_3, respectively (Fig 3). Validation confirmed that the majority of residues were within favorable regions, ensuring the stereochemical quality of the model. Refinement improved structural stability, with overall results from the ProSA servers and the ERRAT online tool showing that the suggested vaccination 3D structures were of higher quality (Fig 4).

The quality and reliability of the predicted tertiary structures of the vaccine constructs were evaluated using ProSA Z-score, Ramachandran plot analysis, ERRAT score and MolProbity score (Table 4). The ProSA Z-scores of the models ranged from −5.48 to −4.51 which fall within the range typically observed for experimentally determined proteins of similar size indicating acceptable overall structural quality. Ramachandran plot analysis revealed that 87.70–93.50% of residues were located in the favored regions suggesting good stereochemical quality of the predicted structures. The ERRAT scores ranged from 81.67 to 88.80 exceeding the commonly accepted threshold of 50 which indicates reliable non-bonded atomic interactions and overall model stability. Additionally, MolProbity scores between 1.83 and 2.06 further confirmed the good stereochemical quality of the models. Overall, these structural validation metrics indicate that all predicted vaccine constructs possess acceptable structural quality.

**Table 4:** Structural validation metrics of the predicted tertiary structures of the vaccine constructs.

Protein ID	Z-Score	Ramachandran Plot	ERRAT	Molprobity Score
HMPV_V1	−5.48	89.80%	81.667	1.83
HMPV_V2	−5.24	87.70%	87.57	2.06
HMPV_V3	−4.51	93.50%	88.796	1.89

### Molecular docking

The effectiveness of the vaccine constructs’ binding to the receptor was determined by molecular docking evaluation. The several dock score arrangements are shown in Table 5. Three distinct criteria were used to decide the selected docked complex: the inclusion of cluster members, the low energy score, and the central location inside the active site. The docking analysis demonstrated differences in predicted binding affinity and conformational stability among the three vaccine constructs. HMPV_V1 showed the lowest individual docking energy (−1141.9) indicating the presence of a highly favorable binding pose; however, the difference between its center (−904) and lowest energy values suggests variability among the docked conformations. In contrast, HMPV_V2 displayed identical center and lowest energy scores (−969.2) indicating that the dominant docking cluster corresponds to a stable and energetically favorable binding conformation. HMPV_V3 formed the largest cluster (35 members) but exhibited comparatively higher energy scores (center: −727.8; lowest: −865), suggesting weaker predicted receptor interaction. Taken together, the docking profile indicates that HMPV_V2 maintains a more consistent and energetically stable interaction pattern, supporting its selection for subsequent analyses.

**Table 5:** Docking score analysis of ligand-receptor complexes. Docking scores calculation between multiple HMPV vaccines and the TLR4 receptor complex.

Protein	Members	Representative	Weighted Score
HMPV_V1	26	Center	−904
		Lowest Energy	−1141.9
HMPV_V2	21	Center	−969.2
		Lowest Energy	−969.2
HMPV_V3	35	Center	−727.8
		Lowest Energy	−865

**Fig 5:**
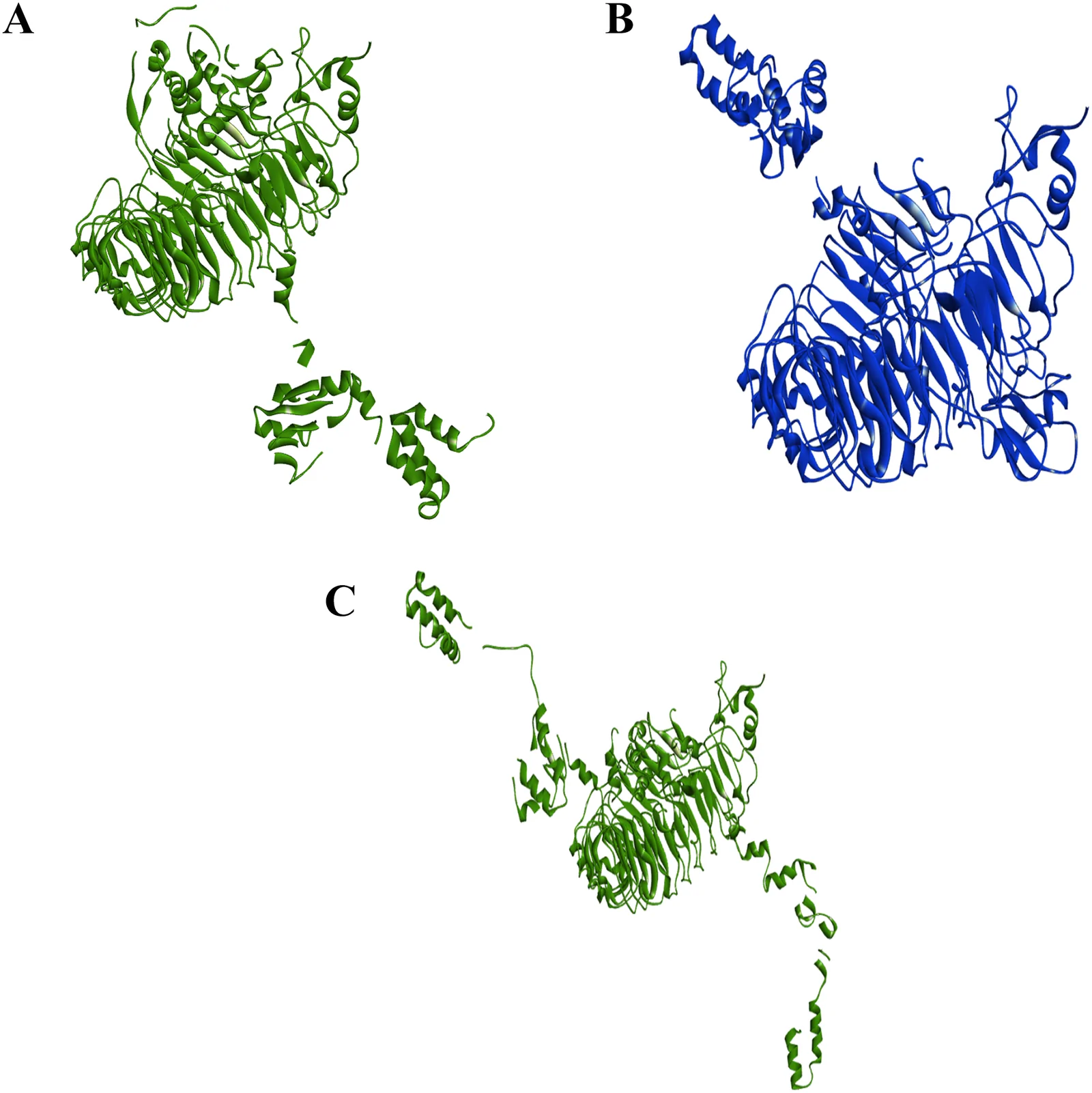
Molecular interaction of HMPV vaccines with the human immune receptor TLR4. Herein, the interactions between (A) HMPV_V1 vaccine and TLR4 receptors (B) HMPV_V2 vaccine and TLR4 receptor. (C) HMPV_V3 vaccine with TLR4 receptor.

Fig 5 also shows the complex files.

### Disulfide engineering of vaccine constructs

Disulfide engineering was performed on the refined structures of all three vaccine constructs to improve their structural stability. DbD2 identified several residue pairs with favorable geometric parameters for cysteine substitution. After excluding native cysteine pairs, vaccine construct 1 exhibited favorable residue pairs including Asn268–Asp294 (0.57 kcal/mol), Ser438–Phe463 (0.65 kcal/mol), Arg289–Ser314 (1.33 kcal/mol) and Leu350–Leu353 (1.42 kcal/mol). Vaccine construct 2 contained favorable residue pairs Asn268–Asp294 (0.44 kcal/mol), Ser438–Phe463 (0.65 kcal/mol), Glu425–Pro264 (1.05 kcal/mol) and Leu350–Leu353 (1.42 kcal/mol). Similarly, vaccine construct 3 showed suitable residue pairs Arg264–Tyr162 (0.54 kcal/mol), Asn268–Asp294 (0.55 kcal/mol), Ser438–Phe463 (0.66 kcal/mol) and Leu350–Leu353 (1.42 kcal/mol). These residue pairs satisfied the recommended geometric criteria and were selected as potential sites for cysteine substitution to improve structural stability. Among the three constructs, vaccine construct 2 retained the most favorable overall structural characteristics following refinement (Table 6).

**Table 6:** Verified candidate residue pairs (excluding native Cys–Cys pairs).

Vaccine construct	Selected residue pair	χ3 (°)	Bond energy (kcal/mol)
HMPV_V1	Asn268–Asp294	−91.13	0.57
	Ser438–Phe463	103.34	0.65
	Arg289–Ser314	−101.01	1.33
	Leu350–Leu353	102.79	1.42
HMPV_V2	Asn268–Asp294	−93.37	0.44
	Ser438–Phe463	103.31	0.65
	Glu425–Pro264	100.20	1.05
	Leu350–Leu353	102.82	1.42
HMPV_V3	Arg264–Tyr162	110.68	0.54
	Asn268–Asp294	−92.66	0.55
	Ser438–Phe463	103.38	0.66
	Leu350–Leu353	102.82	1.42

### Immune simulation

**Fig 6:**
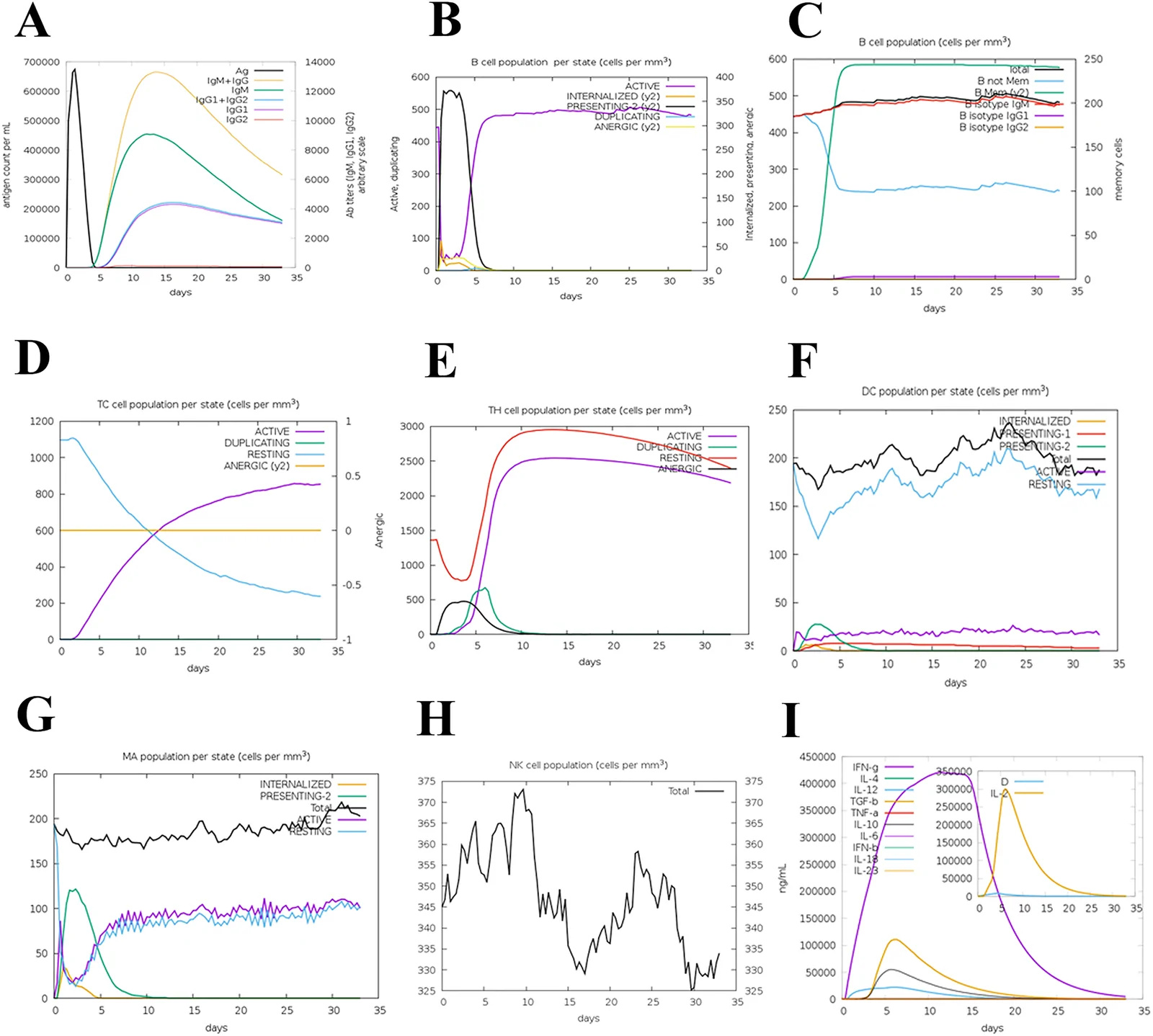
In silico production of an immune reaction using vaccine as antigen. (A) Antigen-induced production of immunoglobulins and B-cell isotypes; (B) number of engaged B-cells per state; (C) number of plasma B-lymphocytes and their isotypes per state; (D) helper T-cell population; (E) helper T-cell population condition throughout subsequent immune reactions; (F) number of cytotoxic T-cells per antigen-exposed state; (G) macrophage population activity in three subsequent immune responses; (H) Dendritic cell population by state and (I) Cytokine and interleukin production using the Simpson index of immune response.

The early peak in antigen concentration followed by a sharp decline (Fig 6A) represents Antigen Processing and Presentation. The stable activity of Dendritic Cells (Fig 6F) and Macrophages (Fig 6G) reflects the successful recognition of L7/L12 adjuvant by TLR4 receptors. This provides the “Signal 0” (innate activation) required to move the antigen from the extracellular space into the MHC pathways. The rise in TC (Fig 6D) and TH (Fig 6E) populations starting after day 5 is the direct result of the structural linkers. The activation of Cytotoxic T-cells is grounded in the AAY linkers. These are optimized for proteasomal cleavage, allowing CTL epitopes to be loaded onto MHC Class I molecules. The rise in Helper T-cells is facilitated by the GPGPG linkers, which ensure the HTL epitopes are flexible enough to be processed and presented via MHC Class II. This provides the “Signal 2” (co-stimulation) necessary for T-cell proliferation. The transition of B cells from “Active” to “Memory” and the dominance of IgG1 over IgM (Fig 6A) indicate Th2-aided B-cell maturation. The plateauing of total B cells after day 10 combined with the rise in Memory B cells (Fig 6C) proves that the vaccine successfully induced Germinal Center reactions. The IgG1 dominance is a biological hallmark of protein-based vaccines that have successfully recruited T-helper cells to trigger antibody class switching. The early peaks of IFN-γ and IL-12 confirm Th1 Polarization. IL-12 is the “instigator” that forces T-cells to produce IFN-γ. For respiratory viruses such as HMPV, effective antiviral immunity depends on coordinated Th1- and Th2-mediated immune responses, with Th1 responses supporting cellular viral clearance and Th2 responses facilitating protective antibody production.. The later rise in IL-10 (Fig 6I) is the body’s natural “brake” to prevent over-inflammation, indicating a balanced and safe immune response rather than a cytokine storm.

Despite the robust immune profile predicted, we acknowledge that *in silico* immune simulators possess inherent limitations. These models represent a simplified version of the human immune system and cannot fully account for the dynamic cellular microenvironments, systemic pharmacokinetics or the impact of post-translational modifications on epitope accessibility. Therefore, while these results provide a strong theoretical foundation for the vaccine’s potential, they should be interpreted as predictive hypotheses. Further *in vitro* assays and *in vivo* trials are essential to validate these computational findings and confirm the vaccine’s clinical safety and efficacy.

### Molecular dynamics simulation

Conformational stability of molecules and atoms was evaluated through Molecular dynamics simulation (MDS) using atomic-level system simulation. We conducted a 100- nanosecond molecular dynamics (MD) simulation to analyze the vaccine’s structural organization. This action evaluated the ligands’ ability to bind to the protein, specifically the cavity in its active site. The molecular dynamics (MD) simulation was analyzed using RMSF, RMSD, and SSE. Root Mean Square Deviation (RMSD) analysis showed stable fluctuations, indicating conformational steadiness over the simulation timeframe. Root Mean Square Fluctuation (RMSF) values suggested limited flexibility in key binding regions. Secondary Structure Elements (SSE) analysis further confirmed that the construct maintained its integrity during dynamic motion.

#### RMSD.

**Fig 7:**
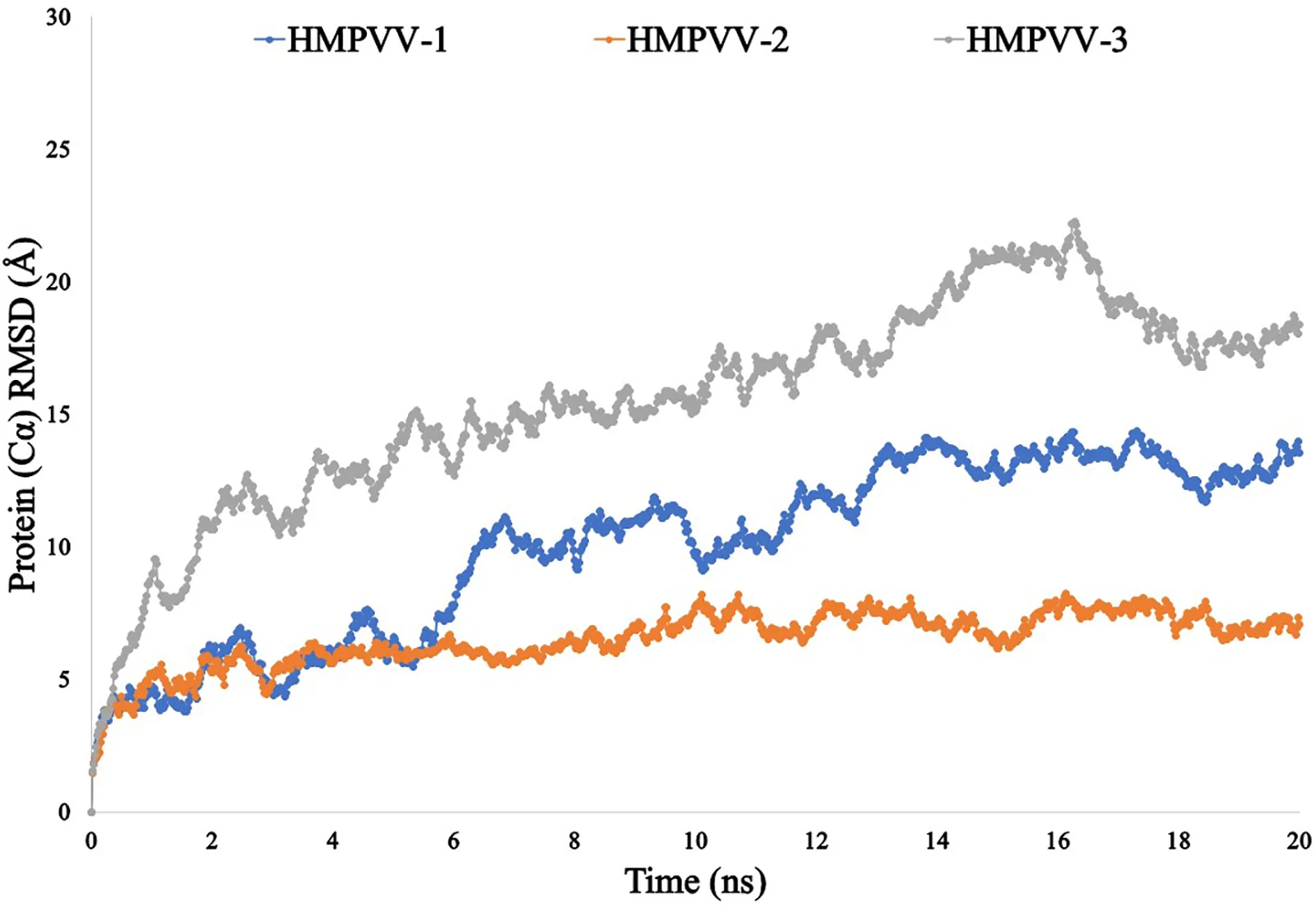
RMSD analysis from molecular dynamic (MD) simulation represents the first 20 ns window of the complete 100 ns simulation. HMPV_V1, HMPV_V2 and HMPV_V3, which are blue, orange and gray, serve as examples of RMSD.

The three vaccine complexes in this 100-nanosecond MDS generated good outcomes. The vaccine complex HMPV_V3, with the highest variation among the three, was identified. The largest significant change (22.291) occurs at 16.28 nanoseconds. HMPV_V1 outperforms HMPVV_3, with a peak deviation rate of 14.352 at 17.62 nanoseconds. Overall, the outcomes are positive. HMPV_V2 reached a stable plateau with a maximum variance of only 8.213 Å at 16.14 ns, maintaining a significantly lower and more consistent profile in Fig 7 and S1 Fig. The large fluctuations in HMPV_V3 and V1 suggest potential structural unfolding or “loop-fraying” during the simulation. In contrast, the rapid equilibration and low deviation of HMPV_V2 indicate a highly stable ligand-receptor complex. Biologically, this structural persistence is essential; a vaccine construct must remain folded within the receptor long enough to initiate the signaling cascade required for innate immune priming.

#### RMSF.

**Fig 8:**
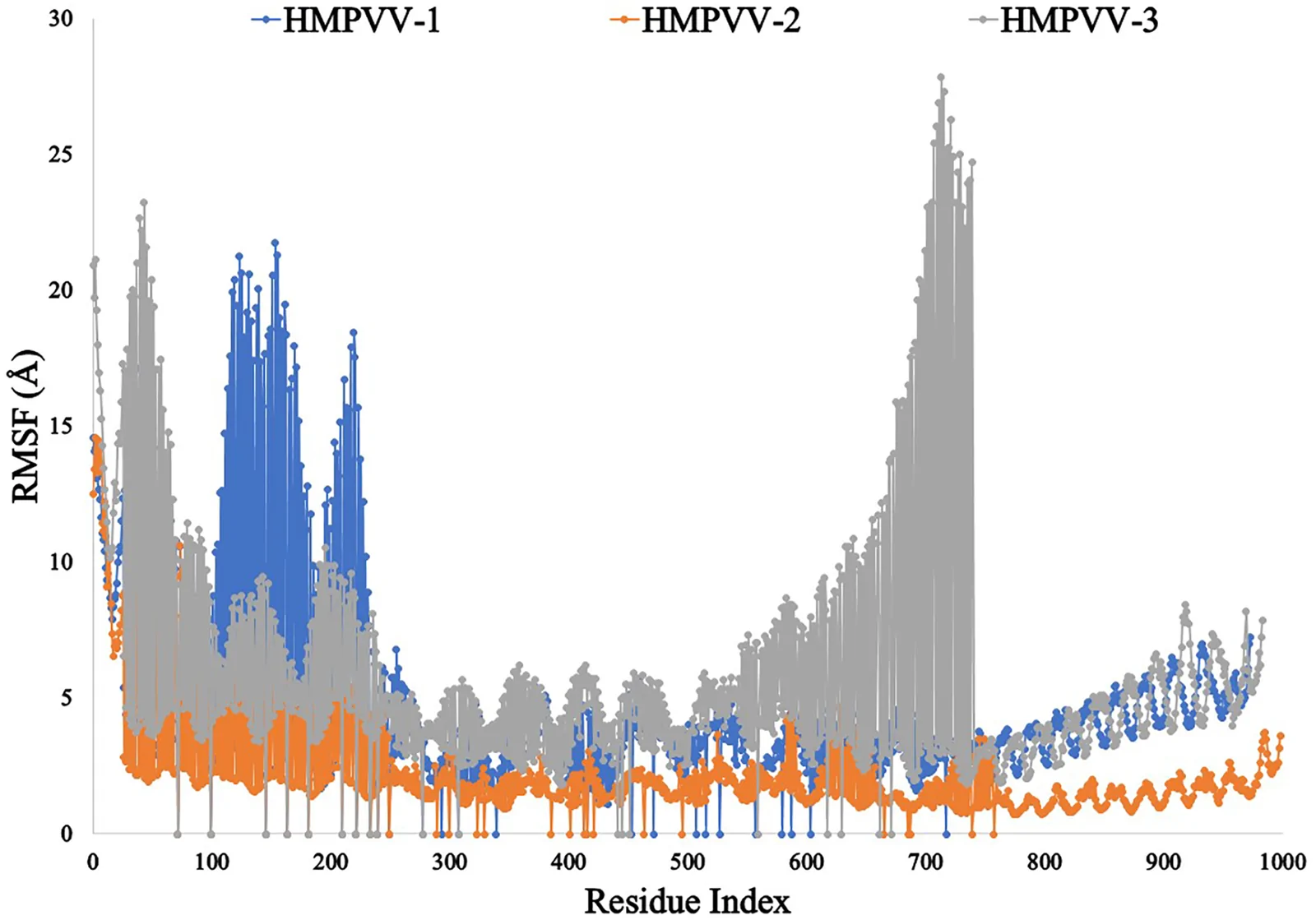
RMSF analysis from MD simulation represents the first 20 ns window of the complete 100 ns simulation. The RMSF of the top three proteins, blue, orange and gray-colored HMPV_V1, HMPV_V2 and HMPV_V3, respectively.

Out of the three proteins, HMPV_V3 shows the greatest variability, according to the MDS data. According to Fig 8 and S2 Fig, the minimum and highest RMSF values for HMPV_V3 are 1.795 and 27.862, respectively. Another protein, HMPV_V1, yields almost the same results. The highest and lowest RMSF values in this case are 21.774 and 1.116, respectively. When compared to the other two, the final protein, Protein HMPV_V2, performs exceptionally well. The graph shows the lowest and maximum RMSF values, which are 0.737 and 14.582, respectively. The low RMSF values in the epitope-containing regions of HMPV_V2 confirm that the AAY and GPGPG linkers provide sufficient structural rigidity to maintain the specific “bioactive” conformation of the epitopes. This local stability ensures that the conformational epitopes remain visible to B-cell receptors (BCRs) and that the linear epitopes are correctly oriented for processing and presentation by Antigen-Presenting Cells (APCs).

#### SSE.

**Fig 9:**
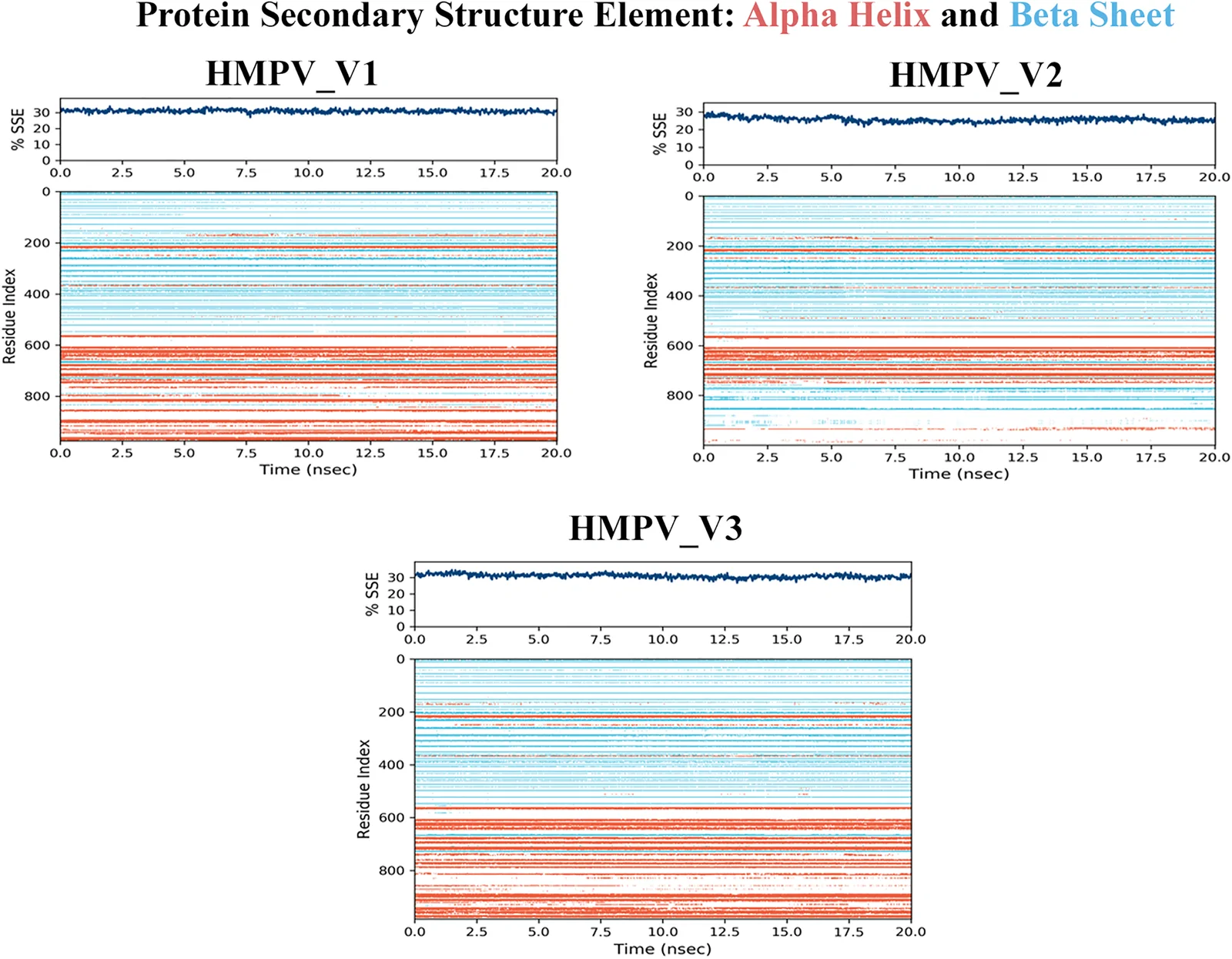
SSE Analysis from molecular dynamics simulation represents the first 20 ns window of the complete 100 ns simulation. Each of the three proteins—HMPV_V1, HMPV_V2, and HMPV_V3—has a unique secondary structure. The alpha helix is red, and the beta-sheet is blue.

Analyzing the shifts and alterations in a protein’s secondary structure during simulation can help determine its conformational stability. Fig 9 shows how β-helices (blue bars) and β-sheets (brown bars) contribute to secondary structure creation. The majority of amino acid residues in domains I and II, which form α-helices and β-sheets in the original structure, maintain secondary structural conformation throughout the simulation, accounting for nearly 100% of the simulation duration. Domain III’s β-sheet-forming amino acid residues preserve protein shape throughout the simulation duration Fig 9.

### Codon optimization and *in-silico* cloning

**Fig 10:**
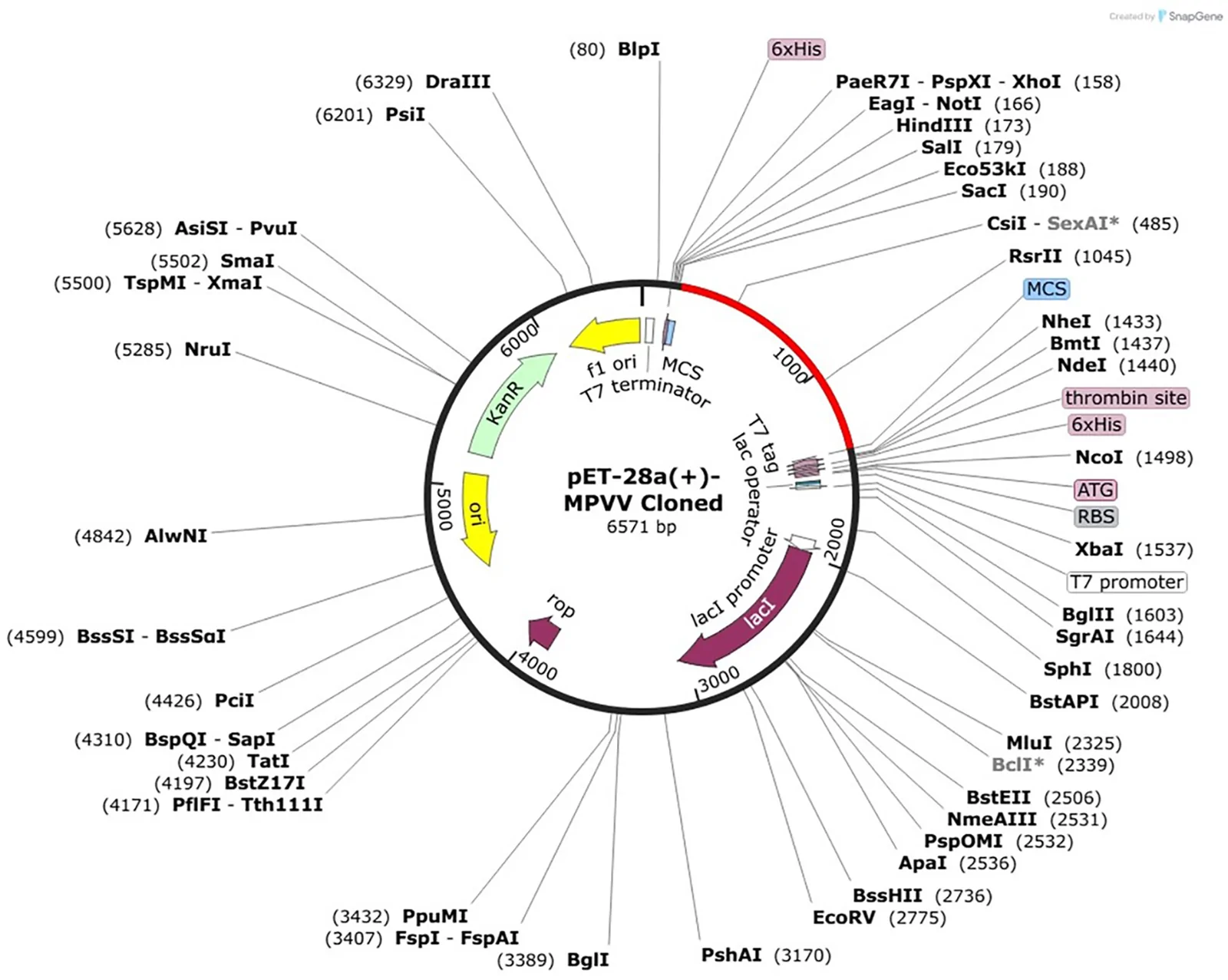
The intended vaccine construct is cloned in silico into an expression vector. The HMPV_V2 vaccine was cloned in silico into the pET28a (+) Escherichia coli expression vector.

The projected GC content and CAI values show that the level of elevated vaccine expression in the E. Coli strain K12 is sufficient. SnapGene software was used to successfully insert the final vaccine design HMPV_V2 into the pET28a (+) vector plasmid in Fig 10.

## Discussion

The integration of reverse vaccinology (RV) and immunoinformatic has emerged as a transformative computational paradigm in modern vaccinology, enabling the rational design of multi-epitope vaccines by identifying highly antigenic, non-toxic, and non-allergenic regions within a pathogen’s proteome. This methodology has been extensively documented in recent years across diverse infectious and non-infectious disease models, including filarial parasites like Wuchereria bancrofti and Brugia malayi [65–67], viral threats such as Monkeypox (Mpox) [68,69] and broad-spectrum pan-corona candidates targeting multiple coronaviruses [70]. Group A Rotavirus [71], human papillomavirus (HPV)-associated cervical cancer [72] and Chandipura Virus [73]. Furthermore, immunoinformatics extends to oncogenic pathogens, as exemplified by the targeting of Fusobacterium nucleatum Fap2 for therapeutic intervention in colorectal cancer [74]. Recent studies have also demonstrated the applicability of these approaches to Human Metapneumovirus (HMPV). For example, Ehsasatvatan et al. (2025) reported a computationally designed multi-epitope HMPV vaccine using integrated immunoinformatics analyses including epitope prediction, structural modeling, molecular docking and immune simulation [75]. Consistent with previous studies, our current study leverages these established frameworks to propose HMPV-V2, a multi-epitope candidate targeting Human Metapneumovirus (HMPV). A distinguishing feature of the present study is the integration of a pan-genome-guided reverse vaccinology strategy to identify conserved target proteins from multiple HMPV genomes before epitope selection. This approach was intended to prioritize conserved epitopes with broader coverage across circulating viral lineages. Nevertheless, similar to other computational vaccine design studies, the predicted immunogenicity, safety, and protective efficacy of HMPV-V2 require further validation through experimental in vitro and in vivo studies before clinical application.

A critical component of the predicted efficacy of HMPVV_2 lies in its interaction with Toll-like receptor 4 (TLR4), a key pattern recognition receptor that initiates innate immune signaling and promotes the activation of adaptive immune responses. Molecular docking followed by molecular dynamics (MD) simulation demonstrated that the HMPVV_2–TLR4 complex remained structurally stable throughout the simulation period suggesting a favorable and sustained interaction between the vaccine construct and the receptor. The low RMSD and RMSF fluctuations observed during the simulation indicate that the complex maintained conformational stability with minimal structural deviations, supporting the reliability of the predicted binding mode. Such structural stability is essential for preserving antigen presentation and may contribute to the induction of a robust and sustained immune response.When compared with other experimental HMPV vaccine candidates, the multi-epitope approach offers unique advantages and challenges. Current leading candidates include mRNA platforms (e.g., mRNA-1653), live-attenuated strains (e.g., Metavac), and prefusion-stabilized (Pre-F) protein subunits [76–78]. While mRNA and subunit vaccines have shown high neutralizing antibody titers and safety in Phase 1 trials [78,79], they often focus on large, single proteins like the F glycoprotein. In contrast, HMPV-V2 is designed to incorporate multiple conserved B-cell and T-cell epitopes from three different proteins, aiming for broader strain coverage and a more balanced humoral and cellular immune response. This strategy directly addresses the genetic diversity of circulating HMPV strains, a challenge that single-antigen approaches may struggle to overcome. However, it must be noted that experimental candidates like AI-guided stabilized Pre-F trimers have already demonstrated near-complete protection in cotton rat models [80], setting a high benchmark for in silico designs and underscoring the need for experimental validation of our computational predictions.

Our findings align with and extend recent immunoinformatic efforts in vaccine development, demonstrating the growing consensus on the utility of computational approaches. For example, a study by Garrido-Palazuelos et al. (2024) on a Staphylococcus aureus vaccine [81] similarly employed a multi-epitope approach, highlighting the importance of selecting highly antigenic and immunogenic epitopes. Their work, much like ours, emphasizes the computational prediction of robust immune responses, including strong T-cell activation and antibody production. The strategic use of specific linkers and adjuvants to enhance immunogenicity, as seen in our HMPV-V2 design, is a common and effective strategy also explored in other studies. For instance, a multi-epitope vaccine against Mycobacterium tuberculosis designed by Sethi et al. (2024) [82] similarly utilized linkers to optimize epitope presentation and adjuvant fusion for enhanced immune stimulation, demonstrating comparable principles in vaccine construct optimization and reinforcing the validity of our design choices.

Furthermore, the rigorous structural validation performed for HMPV-V2, including Ramachandran plot analysis, ProSA Z-scores, and ERRAT scores, is consistent with best practices in immunoinformatic. This level of structural integrity is crucial for the stability and efficacy of the vaccine, as highlighted by studies such as the multi-epitope vaccine design for Streptococcus pneumoniae by Al-Khafaji et al. (2024) [83], where structural stability was a key determinant for selecting the lead candidate. Our molecular dynamics simulations, indicating low RMSD and RMSF values for HMPV-V2, further underscore its predicted stability, a factor also emphasized in the design of a multi-epitope vaccine against Candida auris by Al-Khafaji et al. (2023) [84], where dynamic stability was critical for maintaining antigenicity and ensuring prolonged immune recognition.

The broad global applicability of our predicted epitopes, achieved by selecting MHC class I and II alleles representing major HLA distributions, is a significant strength of our design. This strategy directly addresses the genetic diversity of circulating HMPV strains, a challenge also faced in the development of universal influenza vaccines, as discussed by He et al. (2016) [85], who emphasized the critical need for broad population coverage to ensure vaccine effectiveness across diverse demographics. While we acknowledge the potential underrepresentation of certain ethnic groups, our approach maximizes coverage based on available data, a pragmatic choice also made in other broad-spectrum vaccine designs to achieve the widest possible impact. The predicted Th1-dominant cytokine profile and robust adaptive immune response observed in our immune simulations for HMPV-V2 are highly desirable outcomes, mirroring the immunogenic profiles sought in other computationally designed vaccines, such as the multi-epitope vaccine against SARS-CoV-2 by Al-Khafaji et al. (2021) [86], which also aimed for strong cellular and humoral responses to ensure comprehensive protection.

Human metapneumovirus (HMPV) remains a significant cause of acute respiratory tract infections, particularly among infants, older adults, and immunocompromised individuals. Despite its substantial global disease burden, there is currently no licensed vaccine or specific antiviral therapy available for HMPV [84,85]. Consequently, the development of safe and effective vaccines remains a global research priority. Recent advances in vaccine research have led to the development of several HMPV vaccine candidates including live-attenuated viruses, viral vector-based vaccines, protein subunit vaccines, virus-like particles and mRNA-based platforms. Many of these vaccines have demonstrated promising immunogenicity and protective efficacy in preclinical studies while some have progressed toward early clinical evaluation [14,87]. In this context, immunoinformatic-driven vaccine design offers a rapid and cost-effective strategy for identifying conserved antigenic determinants and constructing multi-epitope vaccine candidates with the potential to elicit broad immune responses. The present study contributes to these ongoing efforts by proposing a rationally designed multi-epitope vaccine candidate against HMPV supported by comprehensive computational analyses. Nevertheless, as the findings are based on in silico predictions further experimental validation through in vitro immunological assays, preclinical animal studies and well-designed clinical investigations will be essential to confirm the vaccine’s safety, immunogenicity and protective efficacy before its potential clinical application.

### Limitations and future directions

Despite the promising computational profile of HMPV-V2, several inherent limitations must be addressed. Our epitope selection for broad global applicability utilized MHC class I and II alleles representing major HLA distributions across European, East and South Asian, African, and West Asian populations. While this provides extensive coverage, we acknowledge potential underrepresentation of specific ethnic groups, such as Indigenous populations from Oceania, Native Americans, and certain Central African communities, due to the exclusion of some region-specific HLA alleles. Furthermore, the computational analysis assumes comparable HLA expression and antigen presentation efficiency across alleles. This standardization is crucial for identifying potential epitopes with predicted binding affinity to widely distributed HLA molecules, allowing for direct comparison across diverse alleles without the confounding effects of variable individual-specific expression levels in vivo. This study predicted linear B-cell epitopes using the BCPred server but discontinuous (conformational) B-cell epitopes were not evaluated using a three-dimensional structure-based prediction method. The inclusion of conformational epitope prediction could provide a more comprehensive assessment of the vaccine’s antibody-binding potential and will be considered in future investigations. In silico predictions are based on simplified mathematical models that cannot fully replicate the stochastic nature of host-pathogen interactions in vivo. For example, epitope competition—where different epitopes compete for binding to a limited number of MHC molecules—could significantly alter the actual immunogenicity of the vaccine. Furthermore, immune dominance may cause the host’s response to focus on only a few epitopes, potentially rendering others ineffective. The choice of a vaccine delivery system (e.g., viral vectors, lipid nanoparticles, or adjuvanted protein subunits) also remains a critical hurdle that cannot be fully resolved through computational means. While our use of the 50S ribosomal protein L7/L12 as an adjuvant and specific linkers (AAY, GPGPG, KK) follows established practices to enhance immunogenicity and processing, their effectiveness is ultimately dependent on the biological context and requires empirical validation.

## Supporting information

S1 Fig RMSD plot of the HMPV vaccine construct illustrating its structural stability throughout the molecular dynamics simulation. (XLSX)

S2 Fig RMSF (Root Mean Square Fluctuation) plot of the HMPV vaccine construct depicting the flexibility of individual residues during the molecular dynamics simulation. (XLSX)

S3 File Complete amino acid sequence of the HMPV-V2 vaccine construct. (DOCX)
