# Supplementary material for: Computational prediction of a multi-epitope Human Metapneumovirus vaccine candidate through integrated reverse vaccinology and pan-genomic approaches
Source: PLoS One. 2026 Sep 15;21(9):e0357758. doi: 10.1371/journal.pone.0357758 (PMC13577472; doi:10.1371/journal.pone.0357758)
Supplement: Supplementary file 1 [file pone.0357758.s003.docx]

**Complete amino acid sequence of the HMPV-V2 multi-epitope vaccine construct:**

MAKLSTDELLDAFKEMTLLELSDFVKKFEETFEVTAAAPVAVAAAGAAPAGAAVEAAEEQSEFDVILEAAGDKKIGVIKVVREIVSGLGLKEAKDLVDGAPKPLLEKVAKEAADEAKAKLEAAGATVTVKEAAAKKTLTITTLYAAYAAMSVLPKRAAYDTYQGIPYTAAVAAYTVCEVKTVYAAYSVLPKRFEVGPGPGLTITTLYAASQSGPILKVGPGPGKSVSIKESESATVEAAGPGPGTIPAFIKSVSIKESESGPGPGSKFVSSAKSVGKKTHDGPGPGIKSVSIKESESATVEAAIKKKNTPVTIPAFIKSVSIKESEKKQLKTLTITTLYAASQSGPKKCEVKTVYLTTMKPYKKDTYQGIPYTAAVQVKKKVNASAQGAAMSVLPKRF
