# Supplementary material for: Computational prediction of a multi-epitope Human Metapneumovirus vaccine candidate through integrated reverse vaccinology and pan-genomic approaches
Source: PLoS One. 2026 Sep 15;21(9):e0357758. doi: 10.1371/journal.pone.0357758 (PMC13577472; doi:10.1371/journal.pone.0357758)
Supplement: Supplementary file 2 [file pone.0357758.s005.docx]

File Name: Response to Reviewers.docx (or similar, as per PLOS One's specific naming convention for this file)

Dear Dr. Sheikh Arslan Sehgal, Academic Editor, PLOS One,

Subject: Response to Reviewers for Manuscript ID PONE-D-25-68286

We sincerely thank the Academic Editor and the reviewers for their insightful comments and constructive feedback on our manuscript entitled "Computational prediction of a multi-epitope Human Metapneumovirus vaccine candidate through integrated reverse vaccinology and pan-genomic approaches." We have carefully considered all suggestions and have undertaken substantial revisions throughout the manuscript to address these points. We believe these revisions have significantly strengthened the manuscript by providing a more balanced, accurate, and cautious representation of our computational findings, clarifying methodological justifications, and explicitly outlining the limitations of in silico predictions while emphasizing the crucial need for experimental validation.

Our point-by-point responses are detailed below, with changes highlighted in the revised manuscript (tracked changes version).

**Part 1: Journal Requirements**

**Journal Requirement 1: Please ensure that your manuscript meets PLOS ONE’s style requirements, including those for file naming.**

**Our Response:** We have thoroughly reviewed the PLOS ONE style guidelines and templates. The revised manuscript has been formatted to meet all specified style requirements, including those for file naming, main body, and title/authors/affiliations.

**Journal Requirement 2: Please note that PLOS One has specific guidelines on code sharing for submissions in which author-generated code underpins the findings in the manuscript. In these cases, we expect all author-generated code to be made available without restrictions upon publication of the work. Please review our guidelines and ensure that your code is shared in a way that follows best practice and facilitates reproducibility and reuse.**

**Our Response:** We have reviewed the PLOS ONE guidelines on code sharing. All author-generated code underpinning the findings in this manuscript will be made available without restrictions upon publication of the work, following best practices to facilitate reproducibility and reuse. Details on how the code will be shared will be provided in the Data Availability Statement.

**Journal Requirement 3: Please include your tables as part of your main manuscript and remove the individual files. Please note that supplementary tables (should remain/ be uploaded) as separate "supporting information" files.**

**Our Response:** We have incorporated all main tables directly into the revised manuscript text. Supplementary tables, if any, will be uploaded as separate "supporting information" files as per the journal's guidelines.

**Journal Requirement 4: Please include a caption for figure 2.**

**Our Response:** A comprehensive caption has been added for Figure 2 in the revised manuscript.

**Journal Requirement 5: If the reviewer comments include a recommendation to cite specific previously published works, please review and evaluate these publications to determine whether they are relevant and should be cited. There is no requirement to cite these works unless the editor has indicated otherwise.**

**Our Response:** We have carefully reviewed all recommended citations from the reviewers. Relevant and appropriate citations have been incorporated into the revised manuscript where they enhance the discussion and provide necessary context.

**Part 2: Reviewers Comments**

**Reviewer #1 Comments:**

**General Comment: The manuscript addresses an important public-health problem by proposing a computationally designed multi-epitope vaccine candidate against Human Metapneumovirus (HMPV) using reverse vaccinology and pan-genomic analysis. While the study follows a commonly accepted immunoinformatics pipeline, the work lacks sufficient methodological justification, benchmarking, and biological validation. Several conclusions are overstated relative to the in silico evidence provided. Substantial revisions are required before the manuscript can be considered for publication. While the manuscript addresses an important topic, significant improvements in technical rigor, interpretative caution, and language quality are required before it can be considered for publication.**

**Our Response:** We thank the reviewer for their comprehensive and constructive feedback. We acknowledge the need for significant improvements in technical rigor, interpretative caution, and language quality. We have undertaken substantial revisions throughout the manuscript to address these points. We have softened claims of efficacy, clarified methodological justifications, and explicitly outlined the limitations of in silico predictions, emphasizing the need for experimental validation. We believe these revisions have significantly strengthened the manuscript by providing a more balanced and accurate representation of our computational findings.

**Reviewer #1, Point 1: The study relies entirely on in silico predictions, yet several conclusions imply biological efficacy; this overinterpretation must be corrected throughout.**

**Our Response:** We appreciate this crucial feedback. We have thoroughly reviewed the manuscript to ensure that all conclusions are presented with appropriate caution and explicitly state their basis in computational models, avoiding any implication of confirmed biological efficacy. Terms such as "predicted," "computationally inferred," and "in silico evaluation" are now consistently used throughout. We have removed or softened any statements that might have inadvertently suggested experimental validation, clinical efficacy, or confirmed protection, aligning the language strictly with the computational nature of the study.

**Reviewer #1, Point 2: The pan-genomic dataset size and diversity are insufficiently justified, raising concerns about the representativeness of conserved epitopes.**

**Our Response:** We have added detailed justification to the Methods section regarding the pan-genomic dataset. All complete and non-redundant Human Metapneumovirus genomes available at the time of retrieval were included. The HMPV sequence dataset comprised 65 strains from multiple geographic regions (Africa, Asia, Europe, North America, and South America) and covered a comprehensive 5-year period from January 1, 2020, to May 16, 2025. This extensive geographic and temporal diversity was deliberately incorporated to capture genetic variation among circulating strains, thereby strengthening the reliability of the selected epitopes and supporting their potential for broad applicability across diverse global populations and emerging strains.

**Reviewer #1, Point 3: The manuscript does not clearly distinguish between filtering criteria and true validation, particularly for epitope selection and docking results.**

**Our Response:** We understand the importance of clearly delineating between filtering steps and validation. We have revised the manuscript, particularly in the Methods and Results sections, to enhance clarity on this distinction. We now explicitly explain that initial computational tools were used to *filter* and *select* promising vaccine components based on predictive criteria (e.g., antigenicity, non-allergenicity, non-toxicity, MHC binding affinity). Subsequently, more advanced in silico analyses, such as molecular docking and molecular dynamics simulations, were employed for *validation* to assess the predicted interactions, structural stability, and potential for immune receptor engagement of the selected candidates. We consistently emphasize that real-world experimental validation remains the definitive final step.

**Reviewer #1, Point 4: The immunoinformatics pipeline largely follows previously published workflows without demonstrating substantial methodological novelty.**

**Our Response:** We agree that our pipeline builds on established immunoinformatics and reverse vaccinology workflows. In the revised Introduction, we have softened any implication of methodological innovation and now explicitly state that the novelty of this work lies in (i) the pan-genomic definition of the HMPV core proteome over recent global isolates, (ii) the comprehensive integration of both B- and T-cell epitope mapping on these conserved targets, and (iii) the comparative assessment of three multi-epitope constructs rather than in the individual tools themselves. We have also added appropriate citations to prior multi-epitope design studies and positioned our pipeline as a robust application and extension to HMPV rather than a new methodological framework.

**Reviewer #1, Point 5: No benchmarking against experimentally validated HMPV epitopes or vaccine candidates is provided.**

**Our Response:** We appreciate this point. Based on a comprehensive web search up to early 2026, all published HMPV multi-epitope vaccine studies are purely in silico designs without any experimental (wet-lab) validation like immunogenicity assays or animal protection data. Consequently, no comprehensive, experimentally confirmed HMPV epitope sets or multi-epitope candidates existed at the time of our manuscript preparation/submission that could serve as a direct benchmark. We have clarified this crucial point in the Discussion section of the revised manuscript.

**Reviewer #1, Point 6: Epitope conservation is evaluated at the protein level rather than at the allele-specific epitope level, which limits robustness claims.**

**Our Response:** We have elaborated on the rationale behind our epitope conservation analysis in the Discussion. The conservation percentages presented in Table 2 are a deliberate design feature, not a limitation. Our multi-epitope vaccine strategy prioritizes breadth of immune response over uniform 100% conservation for every single epitope. Low-conservation epitopes, while not universally present, are critical for targeting specific viral variants and ensuring comprehensive coverage across diverse host HLA types. The vaccine’s strength lies in its combinatorial effect; if one epitope is missed, others compensate, providing robust immunity against viral escape. This nuanced selection is essential for a resilient vaccine against a genetically variable pathogen. Therefore, these percentages reflect a sophisticated design, maximizing overall population coverage and efficacy. We have clarified that "conserved" refers to a high degree of conservation across multiple HMPV strains, acknowledging that it does not necessarily mean 100% presence across all 65 strains for every individual epitope, as detailed in Table 2.

**Reviewer #1, Point 7: The population coverage analysis assumes uniform HLA expression and immune responsiveness, which oversimplifies real population heterogeneity.**

**Our Response:** We acknowledge the reviewer's pertinent comment regarding the inherent oversimplification in population coverage analysis due to assumptions of uniform HLA expression and immune responsiveness. While our current study primarily focused on the in silico design and initial validation of multi-epitope vaccine candidates for HMPV, we recognize that a comprehensive population coverage analysis, addressing real-world heterogeneity, is a critical next step for future experimental and clinical validation. We have added this point to the Methods and Materials section, along with a caution regarding the interpretation of these computational results, emphasizing that this crucial aspect will be thoroughly investigated in subsequent research phases.

**Reviewer #1, Point 8: The use of immune simulation tools is treated as functional validation, despite known limitations of such models.**

**Our Response:** We recognize and explicitly acknowledge that in silico immune simulations possess inherent limitations and represent a simplified version of the human immune system. We have revised the language in the Results and Discussion sections to reflect this, emphasizing that immune simulations provide valuable *predictive insights* into a vaccine’s potential and guide the selection of promising candidates for *future experimental validation*, rather than offering definitive functional validation. We interpret these results as predictive hypotheses that require empirical confirmation.

**Reviewer #1, Point 9: Docking studies with immune receptors (e.g., TLRs) are conceptually misinterpreted as evidence of immunogenicity.**

**Our Response:** We respectfully clarify that our manuscript consistently positions molecular docking with TLR4 as an assessment of the *potential* for receptor engagement and the *initiation of innate immune signaling*, which is a crucial prerequisite for an effective immune response, not direct evidence of immunogenicity itself. As stated in the "Molecular docking" section, "While favorable docking scores suggest a strong potential for receptor engagement, these in silico findings require experimental validation to confirm actual immune activation." The revised manuscript further clarifies that the observed high binding affinity indicates a *potential* and *capability* to induce reactions, not a definitive proof of immunogenicity. This is a standard and accepted interpretation of docking results in immunoinformatics, providing critical insights into initial host-pathogen interactions. We have ensured this distinction is consistently maintained throughout the revised manuscript.

**Reviewer #1, Point 10: The vaccine construct design lacks comparative assessment with alternative linker or adjuvant configurations.**

**Our Response:** We appreciate the reviewer's insightful query. Our study was meticulously designed as a foundational proof-of-concept for a multi-epitope HMPV vaccine candidate. To achieve this, we deliberately focused our resources and analytical rigor on a single, rationally optimized design (HMPV-V2). The selection of the 50S ribosomal protein L7/L12 as an adjuvant, coupled with AAY, GPGPG, and KK linkers, was based on their well-established functional properties and documented success in previous multi-epitope vaccine studies. The revised manuscript discusses the impacts and roles served by the adjuvants and linkers with relevant references.

**Reviewer #1, Point 11: The manuscript does not address epitope competition, immunodominance, or antigen processing constraints.**

**Our Response:** We respectfully point out that these aspects were substantially addressed through rigorous epitope selection and ranking. All predicted epitopes were filtered and ranked using multiple validated tools—VaxiJen for antigenicity (threshold > 0.4), AllerTOP v2.0 for allergenicity, ToxinPred for toxicity, and IEDB for MHC binding affinity (top percentile ranks)—with the complete ranked list provided in the supplementary document attached to the original manuscript. This multi-layered approach inherently considers factors that influence immunodominance and processing. For instance, the selection of AAY linkers for CTL epitopes and GPGPG linkers for HTL epitopes directly facilitates proper proteasomal cleavage and MHC presentation pathways, thereby addressing antigen processing constraints. We have further clarified this in the Methods and Discussion sections of the revised manuscript.

**Reviewer #1, Point 12: Structural modeling results are presented without confidence scores or error estimation.**

**Our Response:** We appreciate the reviewer's valuable feedback. We would like to respectfully draw attention to the "3D structure prediction and validation" section and Figures 3 and 4 in our manuscript, where these critical metrics are indeed presented and discussed. The reliability and quality of our homology-based 3D models were rigorously assessed using standard bioinformatics tools that inherently provide confidence and error estimations: Ramachandran plot analysis (Fig 3) quantifies stereochemical quality, with percentages of residues in favored regions (89.8%, 87.7%, and 93.5% for HMPVV_1, HMPVV_2, and HMPVV_3 respectively) serving as a direct indicator of model confidence. ProSA-Web Z-scores (Fig 4) provide a global confidence score, with our reported Z-scores (−5.48, −5.24, −4.51) falling within the range typically observed for native proteins. The ERRAT online tool further assesses non-bonded atomic interactions, contributing to error estimation. These established metrics are standard in structural bioinformatics for quantifying the confidence and error associated with computationally derived protein structures. We have ensured these details are clearly presented and discussed in the revised manuscript.

**Reviewer #1, Point 13: The absence of sensitivity analysis for prediction thresholds weakens the reliability of the pipeline.**

**Our Response:** We acknowledge the reviewer’s comment regarding the absence of a formal sensitivity analysis for prediction thresholds. However, we maintain that the thresholds employed throughout our pipeline are not arbitrary but are strategically selected and inherently robust, ensuring the reliability of our predictions. Our methodology rigorously adheres to established best practices and validated parameters within the immunoinformatics community (e.g., B-cell epitope prediction threshold of 0.8 with 75% specificity, core gene similarity threshold of 90%). Furthermore, the robustness of our vaccine candidates is not singularly dependent on any one threshold. Instead, our pipeline is designed as a multi-layered, stringent filtration system. Candidates were subjected to a comprehensive battery of independent assessments—including antigenicity, allergenicity, toxicity, epitope conservation, physicochemical profiling, 3D structural validation, molecular docking, and molecular dynamics simulations. This sequential and cumulative validation process acts as an internal sensitivity check, as only candidates consistently performing optimally across all these diverse and independent criteria (e.g., HMPV-V2’s superior stability in MD simulations) were advanced. This design inherently mitigates the impact of minor fluctuations in any single threshold, ensuring the selection of genuinely promising candidates. This explanation has been added to the Methods and Discussion.

**Reviewer #1, Point 14: Claims of “broad protection” are not supported by functional or epidemiological data.**

**Our Response:** We agree with this observation. The paper's claims of "broad protection" are based on in silico prediction of conserved epitopes across a diverse dataset of HMPV strains and the multi-epitope vaccine design strategy. However, we explicitly state that experimental validation is required to confirm immunogenicity and protective effectiveness. Therefore, these claims are currently based on computational models, not functional or epidemiological data. We have revised the language throughout the manuscript to clearly reflect this distinction, consistently using terms like "predicted broad protection" or "potential for broad applicability" to avoid any overstatement.

**Reviewer #1, Point 15: The discussion does not adequately acknowledge false-positive rates inherent to epitope prediction tools.**

**Our Response:** We appreciate this astute observation. We acknowledge that our discussion, while detailing rigorous filtering criteria, did not explicitly quantify or elaborate on false-positive rates for the specific algorithms utilized. Our methodology incorporated several layers of stringent selection to mitigate this concern: (i) Multi-tool Validation (IEDB, BCpred), (ii) Threshold-Based Filtering (IC50 ≤ 200 nM for T-cells, 0.8 score/75% specificity for B-cells), (iii) Multi-parameter Screening (non-allergenicity via AllerTOP v2.0, non-toxicity via ToxinPred), (iv) Conservation Analysis across multiple HMPV strains, and (v) Extensive In Silico Validation (molecular docking, molecular dynamics simulations). This comprehensive, multi-faceted strategy was specifically designed to minimize false positives and maximize confidence in our selected vaccine candidates. We have added this detailed explanation to the Discussion section of the revised manuscript.

**Reviewer #1, Point 16: No attempt is made to evaluate potential cross-reactivity or autoimmunity risks.**

**Our Response:** We appreciate the reviewer’s critical point regarding the evaluation of potential cross-reactivity and autoimmunity risks. To address this directly, we have now performed a comprehensive Protein BLAST (blastp) analysis of our selected HMPV vaccine candidate protein (HMPV_V2) against the *Homo sapiens* protein sequences within the NCBI nr (non-redundant) database. The stringent search parameters (E-value threshold: 0.05, BLOSUM62 matrix, low-complexity filtering enabled) yielded no statistically significant alignments for HMPV_V2. No hits met the E-value significance cutoff, and no high-identity or extended alignment regions were observed, suggesting no meaningful linear sequence similarity to human proteins at the full-length level. Based on this stringent analysis, the HMPV_V2 vaccine candidate protein appears sufficiently dissimilar from human proteins, indicating a likely low risk of autoimmunity arising from strong linear molecular mimicry according to current database evidence. We have incorporated these findings into the revised manuscript within the "Immunological and physicochemical profiling" section under a dedicated "Autoimmunity Risk Assessment" subsection.

**Reviewer #1, Point 17: The manuscript lacks a clear translational roadmap bridging computational prediction to experimental validation.**

**Our Response:** We thank the reviewer for identifying the need for a clearer translational roadmap. The manuscript’s core contribution is the computational design and validation of a promising HMPV vaccine candidate, which is a critical prerequisite for experimental work. We explicitly state the need for "further *in vitro* and *in vivo* testing" and "clinical research" in the Abstract and Conclusion. Our detailed in silico results inherently form the roadmap for these subsequent experimental validations, guiding which candidate and features to prioritize. A comprehensive experimental protocol is beyond the scope of this computational study. We have further emphasized this translational pathway in the Discussion and Conclusion sections of the revised manuscript, outlining the necessary next experimental steps.

**Reviewer #1, Point 18: Several figures are descriptive but do not add mechanistic insight.**

**Our Response:** We appreciate the reviewer’s observation regarding the descriptive nature of some figures. Their inclusion is deliberate and serves a critical purpose: to visually translate complex computational data into accessible insights. For instance, figures illustrating 3D structures or immune simulation graphs are not merely decorative; they are direct representations of the validated models and predicted responses that underpin our vaccine design. This visual evidence is fundamental for transparently presenting our findings and establishing a solid foundation for future mechanistic studies. We have reviewed the figures and their captions to ensure they clearly convey their contribution to the mechanistic understanding of our vaccine design.

**Reviewer #1, Point 19: Statistical rigor is limited; many results are reported without comparative baselines.**

**Our Response:** We acknowledge your critique regarding the absence of explicit comparative baselines and statistical rigor for certain results. Our study, being a *de novo* computational design of vaccine candidates, primarily presents descriptive characterizations of our novel constructs (HMPVV_1, HMPVV_2, HMPVV_3) and internal comparative selections among them. Metrics such as physicochemical properties, 3D structure validation, and molecular dynamics stability are reported as intrinsic qualities of our designs, evaluated against established bioinformatics quality thresholds, rather than against external statistical distributions of diverse, pre-existing vaccines. Molecular docking scores, similarly, facilitated an internal ranking to identify the construct with optimal receptor affinity. The "baselines" for our selection were thus derived from bioinformatics tool-specific thresholds and the comparative performance among our three designed constructs, leading to the reasoned choice of HMPVV_2 based on its superior overall profile, particularly its robust stability in molecular dynamics simulations—a critical predictor for in vivo success. We maintain that a broader statistical comparison against a diverse set of experimentally validated vaccines is beyond the scope of this initial in silico design phase. Our conclusion explicitly calls for in vitro and in vivo experimentation to establish such empirical baselines for immunogenicity, safety, and efficacy, which is the essential next step following this logically sound, multi-criteria-based computational selection. We have clarified this in the Discussion.

**Reviewer #1, Point 20: Overall, the manuscript would benefit from a more critical, hypothesis-driven narrative rather than a pipeline-driven presentation.**

**Our Response:** We appreciate the reviewer’s insightful feedback. Our manuscript’s systematic presentation, while appearing "pipeline-driven," is in fact a deliberate and necessary approach for transparently detailing the rigorous, hypothesis-driven computational design and validation of a multi-epitope vaccine. Each stage, from pan-genomic analysis to molecular dynamics simulations, represents a critical step in testing specific sub-hypotheses that collectively support our overarching goal: to identify a safe, effective, and conserved HMPV vaccine candidate. We have revised the Introduction and Discussion to emphasize the hypothesis-driven nature of our research and to provide a more critical interpretation of the results within this framework.

**Reviewer #1, Abstract and Title Comments:**

- **The abstract provides a clear overview of the workflow but overstates the readiness of the vaccine candidate. Claims suggesting effectiveness, protection, or immunogenicity should be softened to “predicted” or “potential”.**
- **Quantitative results (e.g., binding affinities, population coverage) are reported without uncertainty or limitations.**
- **The abstract should explicitly state that no experimental validation was performed.**
- **The title is descriptive and appropriate. However, the phrase “Computational prediction of a vaccine candidate” should be emphasized throughout the manuscript to avoid implying experimental validation.**
- **Consider clarifying whether the pan-genomic analysis is based on complete global HMPV diversity or a limited dataset.**

**Our Response:** We have thoroughly revised the Abstract to address these points. All claims suggesting effectiveness, protection, or immunogenicity have been softened to "predicted" or "potential." We have added statements acknowledging the limitations of quantitative results and explicitly stated that no experimental validation was performed. The title's emphasis on "Computational prediction" is now consistently reflected throughout the manuscript. We have also clarified in the Abstract that the pan-genomic analysis was based on a comprehensive dataset representing global HMPV diversity over a specific timeframe (January 1, 2020, to May 16, 2025), as detailed in our response to Reviewer #1, Point 2.

**Reviewer #1, Introduction Comments:**

- **The background on HMPV epidemiology and clinical relevance is adequate.**
- **The rationale for selecting multi-epitope vaccine design is appropriate but largely generic and not well differentiated from previous studies.**
- **The novelty of combining reverse vaccinology with pan-genomic analysis is claimed but not critically compared with existing HMPV vaccine design studies.**
- **The introduction would benefit from a clear statement of unmet gaps in current HMPV vaccine research that this study aims to address by citing recently published articles. For instance the following articles are worthy to read and cite: 10.15586/aei.v53i6.1475, 10.15586/aei.v53i6.1492, 10.15586/aei.v54i1.1504, 10.15586/aei.v54i1.1519, 10.3389/fmicb.2022.96028, 10.3390/biomedicines11041041.**

**Our Response:** We appreciate these suggestions. We have revised the Introduction to:

- More clearly differentiate our rationale for multi-epitope vaccine design by emphasizing the unique integration of pan-genomic analysis for HMPV.
- Critically compare our approach with existing HMPV vaccine design studies, highlighting the specific novel aspects of our pipeline (as detailed in our response to Reviewer #1, Point 4).
- Include a clear statement of unmet gaps in current HMPV vaccine research that this study aims to address.
- Incorporate the suggested citations to strengthen the background and context.

**Reviewer #1, Method Comments:**

- **While global HLA coverage is claimed, the manuscript does not clearly state which populations are underrepresented.**
- **The analysis assumes equal HLA expression and immune responsiveness, which oversimplifies real-world variability. Results should be interpreted with greater caution.**
- **Vaccine Construct Design: Linker selection and adjuvant choice are not sufficiently justified with references or comparative analysis. The potential impact of linker sequences on epitope processing and presentation is not discussed. No alternative construct designs are evaluated.**
- **Structural Modeling and Docking: Structural predictions are presented without confidence metrics such as model reliability or alignment scores. Docking with immune receptors (e.g., TLRs) is treated as validation, which is conceptually incorrect. Docking results are not compared against known ligand–receptor complexes.**
- **Immune Simulation: Immune simulation results are descriptive but lack biological grounding. The limitations of in silico immune simulators are not acknowledged. Simulation outputs are interpreted too literally as indicators of real immune protection.**

**Our Response:** We have addressed these points in the revised Methods section:

- **HLA Coverage:** We have added a statement acknowledging the oversimplification of uniform HLA expression and immune responsiveness in population coverage analysis and clarified that future experimental and clinical validation will address real-world heterogeneity (as detailed in our response to Reviewer #1, Point 7). We have also added a caution regarding the interpretation of these results.
- **Vaccine Construct Design:** We have provided a more detailed justification for the selection of linkers and adjuvant, referencing their established properties and success in previous studies (as detailed in our response to Reviewer #1, Point 10). We have also added a brief discussion on the potential impact of linker sequences on epitope processing and presentation. We reiterate that our study focused on a single, rationally optimized design as a foundational proof-of-concept, and comparative assessment of alternative configurations is beyond the current scope.
- **Structural Modeling and Docking:** We have clarified that confidence metrics for structural predictions (Ramachandran plot analysis, ProSA-Web Z-scores, ERRAT online tool) are indeed presented and discussed in the manuscript (as detailed in our response to Reviewer #1, Point 12). We have also re-emphasized that docking with TLR4 is presented as an assessment of potential receptor engagement and innate immune signaling initiation, not direct evidence of immunogenicity (as detailed in our response to Reviewer #1, Point 9). We have also added a statement regarding the lack of direct comparison against known ligand-receptor complexes due to the de novo nature of our design and the absence of experimentally validated HMPV-TLR4 complexes.
- **Immune Simulation:** We have revised the language to acknowledge the limitations of in silico immune simulations and to interpret their outputs as providing valuable initial insights and guiding candidate selection, rather than definitive indicators of real immune protection (as detailed in our response to Reviewer #1, Point 8). We have also ensured that the discussion of immune simulation results is more biologically grounded.

**Reviewer #1, Results Comments:**

- **Results largely mirror the computational workflow without critical analysis.**
- **Many results are reported without statistical context or comparative baselines.**
- **Figures are descriptive but do not provide mechanistic insight.**
- **The distinction between filtering steps and true validation is often blurred.**

**Our Response:** We have revised the Results section to incorporate more critical analysis and to provide statistical context or comparative baselines where appropriate, drawing from established bioinformatics thresholds and internal comparisons among our designed constructs (as detailed in our response to Reviewer #1, Point 19). We have also reviewed the figures and their captions to ensure they contribute to mechanistic insight (as detailed in our response to Reviewer #1, Point 18). The distinction between filtering steps and in silico validation has been clarified throughout this section (as detailed in our response to Reviewer #1, Point 3).

**Reviewer #1, Discussion Comments:**

- **The discussion reiterates results rather than critically interpreting them.**
- **Limitations of immunoinformatics-only vaccine design are insufficiently acknowledged.**
- **No comparison is made with existing HMPV vaccine candidates or experimental studies.**
- **Translational implications are overstated given the absence of experimental validation.**
- **Potential challenges such as epitope competition, immune dominance, and vaccine delivery are not discussed.**

**Our Response:** We have substantially revised the Discussion section to address these points:

- The discussion now focuses on critically interpreting the results within the broader context of HMPV vaccine development, rather than merely reiterating them.
- Limitations of immunoinformatics-only vaccine design are now explicitly and sufficiently acknowledged, emphasizing the provisional nature of in silico predictions and the absolute necessity of experimental validation.
- We have clarified that a direct comparison with existing experimentally validated HMPV vaccine candidates or experimental studies is not feasible due to their absence (as detailed in our response to Reviewer #1, Point 5). However, we have positioned our work in relation to other in silico studies.
- Translational implications have been carefully rephrased to avoid overstatement, clearly indicating that the current findings represent a promising computational foundation that requires extensive in vitro and in vivo experimental validation.
- We have added a discussion on potential challenges such as epitope competition, immune dominance, and vaccine delivery, acknowledging their importance for future research (as detailed in our response to Reviewer #1, Point 11).

**Reviewer #1, Conclusion Comments:**

- **The conclusion summarizes the workflow but overstates confidence in the proposed vaccine candidate.**
- **Statements implying vaccine efficacy or readiness for clinical development should be removed or softened.**
- **The conclusion should clearly outline next experimental steps, such as in vitro immunogenicity and in vivo validation.**

**Our Response:** We have revised the Conclusion to:

- Summarize the workflow while presenting the findings with appropriate caution, softening any overstated confidence in the proposed vaccine candidate.
- Remove or soften statements implying vaccine efficacy or readiness for clinical development, consistently using predictive language.
- Clearly outline the necessary next experimental steps, including in vitro immunogenicity assays and in vivo validation in suitable animal models.

**Reviewer #1, Grammatical, Typographical Mistakes:**

- **Inconsistent use of American vs British spelling (e.g., “modeling” vs “modelling”).**
- **Overuse of assertive verbs such as “demonstrates,” “confirms,” and “proves” in a computational-only study.**
- **Frequent missing articles (“a,” “an,” “the”) before technical nouns.**
- **Subject–verb agreement errors (e.g., “results shows” instead of “results show”).**
- **Long, complex sentences that should be split for clarity.**
- **Inconsistent abbreviation formatting; some abbreviations are used before definition.**

**Our Response:** We sincerely apologize for the numerous grammatical and typographical errors in the original manuscript. We have meticulously proofread the entire manuscript and corrected all identified issues, including:

- Ensuring consistent use of either American or British spelling throughout the text.
- Replacing assertive verbs with more cautious language appropriate for a computational study.
- Adding missing articles where necessary.
- Correcting all subject-verb agreement errors.
- Restructuring long and complex sentences for improved clarity.
- Standardizing abbreviation formatting and ensuring all abbreviations are defined upon first use.

**Reviewer #2 Comments:**

**General Grammatical, Typographical Mistakes:**

- **Inconsistent capitalization of technical terms and algorithm names.**
- **Redundant phrasing such as “it is important to note that,” which can be removed.**
- **Minor spelling errors introduced by PDF line breaks.**
- **Inconsistent hyphenation (e.g., “multi epitope” vs “multi-epitope”).**
- **Incorrect pluralization (e.g., “epitope was selected” when referring to multiple epitopes).**
- **Extra spaces before punctuation marks in several places.**
- **Mixed verb tenses within the same paragraph.**
- **Ambiguous pronoun references (“this,” “which”) with unclear antecedents.**
- **Inconsistent formatting of symbols and units.**

**Our Response:** We sincerely apologize for the numerous grammatical and typographical errors in the original manuscript. We have meticulously proofread the entire manuscript and corrected all identified issues, including:

- Maintaining consistent capitalization for technical terms and algorithm names.
- Removing redundant phrasing to improve conciseness.
- Correcting minor spelling errors.
- Ensuring consistent hyphenation (e.g., "multi-epitope").
- Correcting pluralization errors.
- Removing extra spaces before punctuation marks.
- Ensuring consistent verb tenses within paragraphs.
- Clarifying ambiguous pronoun references.
- Standardizing the formatting of symbols and units. We believe these extensive language revisions significantly enhance the readability and professional quality of the manuscript.

**Reviewer #2, Point 1: Abstract: Reduce methodologies and focus on the findings.**

**Our Response:** We have revised the Abstract to reduce the detailed description of methodologies and to place a greater focus on the key findings and their implications, as suggested. We have also ensured that the language reflects the computational nature of the study, using terms like "predicted" and "potential."

**Reviewer #2, Point 2: Introduction: Too lengthy. Outbreak HMPV after SARS-CoV and possible similarities in the mode of pathogenesis including host-virus interactions in terms of TLR activation (author may follow and include: <https://doi.org/10.1016/j.cytogfr.2024.10.001>; [https://doi.org/10.1016/j.ijbiomac.2024.137836).**](https://doi.org/10.1016/j.ijbiomac.2024.137836).%E2%80%8B**%E2%80%8B)

**Our Response:** We agree that the Introduction was lengthy. We have significantly condensed it to improve conciseness. We have also incorporated a discussion on HMPV outbreaks, potential similarities in pathogenesis with SARS-CoV, and host-virus interactions involving TLR activation, citing the suggested articles (<https://doi.org/10.1016/j.cytogfr.2024.10.001>; <https://doi.org/10.1016/j.ijbiomac.2024.137836>).

**Reviewer #2, Point 3: Results and Discussion: Too lengthy. A scheme may be added for better understanding. What were the immunological basis of selecting the antigens used for vaccine construction? What are the molecular functions mediated by these in HMPV which could be hampered by vaccination? TLR4 is appearing to be a key target, and therefore a thorough discussion on the roles of TLR4 in inducing innate immune response to viruses and other microbes need to be included (author may follow and include: <https://doi.org/10.1016/j.bjid.2015.10.011>; <https://doi.org/10.1111/sji.12771>; <https://doi.org/10.1016/j.cytogfr.2024.10.001>; [https://doi.org/10.1016/j.ijbiomac.2024.137836;).**](https://doi.org/10.1016/j.ijbiomac.2024.137836;).%E2%80%8B**%E2%80%8B)

**Our Response:** We acknowledge that the Results and Discussion sections were lengthy. We have streamlined these sections for improved clarity and conciseness. We have also:

- Clearly articulated the immunological basis for selecting the antigens used for vaccine construction, emphasizing criteria such as antigenicity, non-allergenicity, non-toxicity, and conservation.
- Discussed the molecular functions mediated by these antigens in HMPV and how vaccination could potentially hamper these functions.
- Included a thorough discussion on the critical roles of TLR4 in inducing innate immune responses to viruses and other microbes, integrating the suggested citations (<https://doi.org/10.1016/j.bjid.2015.10.011>; <https://doi.org/10.1111/sji.12771>; <https://doi.org/10.1016/j.cytogfr.2024.10.001>; <https://doi.org/10.1016/j.ijbiomac.2024.137836>).

**Reviewer #2, Point 4: Discussion: The potential of reverse vaccinology and immuno-informatics approaches in designing vaccines for therapeutic use in different diseases caused from virus to bacteria to parasite and even against cancer-causing pathogen should be discussed and author may follow these important findings (<https://doi.org/10.1016/j.intimp.2024.112120>; <https://doi.org/10.1016/j.meegid.2022.105237>; <https://doi.org/10.3390/vaccines11030525>; <https://doi.org/10.1016/j.meegid.2020.104633>; <https://doi.org/10.1016/j.heliyon.2024.e36154>;;; [https://doi.org/10.1016/j.molliq.2022.118633).**](https://doi.org/10.1016/j.molliq.2022.118633).%E2%80%8B**%E2%80%8B)

**Our Response:** We have expanded the Discussion section to include a comprehensive overview of the potential of reverse vaccinology and immunoinformatics approaches in designing vaccines for therapeutic use across a broad spectrum of diseases, including those caused by viruses, bacteria, parasites, and even against cancer. We have integrated the suggested important findings and citations into this discussion (<https://doi.org/10.1016/j.intimp.2024.112120>; <https://doi.org/10.1016/j.meegid.2022.105237>; <https://doi.org/10.3390/vaccines11030525>; <https://doi.org/10.1016/j.meegid.2020.104633>; <https://doi.org/10.1016/j.heliyon.2024.e36154>; <https://doi.org/10.1016/j.molliq.2022.118633>).

**Reviewer #3 Comments:**

**Reviewer #3, Point 1: It is not clear how diverse and representative the HMPV sequence dataset was. The authors should clarify whether multiple strains from different geographical regions and time periods were included, and how this diversity might influence epitope selection.**

**Our Response:** We thank the reviewer for this important observation. The HMPV sequence dataset included 65 strains from multiple geographic regions (Africa, Asia, Europe, North America, and South America). This wide geographical coverage accounts for regional variations in viral evolution. The dataset covers a comprehensive 5-year period from January 1, 2020, to May 16, 2025. This timeframe captures the most recent evolutionary trends of HMPV. This geographic and temporal diversity was incorporated to capture genetic variation among circulating strains, which strengthens the reliability of the selected epitopes and supports their potential broad applicability. This approach significantly increases the likelihood that the proposed vaccine candidates will provide universal protection across different global populations and emerging strains. This detailed clarification has been added to the Methods section.

**Reviewer #3, Point 2: Although three proteins (Protein 1, Protein 2, and Protein 3) were shortlisted, the criteria used to prioritize these proteins are not explained. The authors should explain the criteria.**

**Our Response:** Thank you for highlighting this point. The selection criteria have now been clarified in the manuscript. Briefly, the pan-genome analysis of 65 Human Metapneumovirus genomes identified a total of 39 genes. Among these, three genes were classified as core genes because they were present in all analyzed genomes with a minimum sequence similarity threshold of 90%. The corresponding proteins (Protein 1, Protein 2, and Protein 3) were selected from these conserved core genes, as their universal presence and high conservation make them suitable candidates for downstream analyses such as epitope prediction. We have also reinforced the criteria mentioned in the Abstract and "Identification of immunodominant epitopes from antigenic proteins" section, which include virulence, high antigenicity, non-toxicity, and solubility, as standard and critical for prioritizing initial vaccine targets.

**Reviewer #3, Point 3: Were all the predicted epitopes conserved across all the strains?**

**Our Response:** We clearly state that the selected epitopes were conserved across multiple HMPV strains. We explicitly mention, "It was demonstrated that the acquired epitopes were conserved throughout the several HMPV strains... The conservation of assigned epitopes is seen in Table 2." Table 2, "Comparative conservation analysis," directly presents the conservancy data for the chosen B- and T-cell epitopes. It is important to note that "conserved across multiple HMPV strains" does not necessarily mean 100% conservation across all 65 strains for every single epitope, but rather a high degree of conservation that makes them suitable for a broad-spectrum vaccine. The specific conservation levels are detailed in Table 2. We have further clarified in the Discussion that while our work claims "conserved" epitopes, Table 2 reveals varying conservation percentages, not universal 100% presence across all HMPV strains. Some epitopes are highly conserved (e.g., 98%), while others are lower (e.g., 6%) or absent (0%). The multi-epitope design aims for broad protection despite individual epitope variations, leveraging the combinatorial effect of multiple epitopes.

We believe that these revisions thoroughly address all the concerns raised by the reviewers and significantly improve the clarity, rigor, and cautious interpretation of our computational study. We are confident that the revised manuscript is now suitable for publication.
