# Supplementary material for: Computational prediction of a multi-epitope Human Metapneumovirus vaccine candidate through integrated reverse vaccinology and pan-genomic approaches
Source: PLoS One. 2026 Sep 15;21(9):e0357758. doi: 10.1371/journal.pone.0357758 (PMC13577472; doi:10.1371/journal.pone.0357758)
Supplement: Supplementary file 3 [file pone.0357758.s006.docx]

**Reviewer #3 Comments and Our Responses:**

**1. Does the dataset include balanced representation of all HMPV sub-lineages, or are some groups overrepresented? How was genetic variability within each lineage quantified to ensure comprehensive epitope coverage?**

**Response:** We appreciate this important question regarding dataset representation and genetic variability. We designed our study to ensure broad coverage and address genetic diversity as comprehensively as possible within a pan-genomic framework. Our dataset comprised **65 HMPV genome sequences** retrieved from the NCBI Virus database. These sequences were specifically selected to include strains from **multiple geographic regions (Africa, Asia, Europe, North America, and South America)** and spanned a **5-year period (January 1, 2020, to May 16, 2025)**. This extensive global and temporal sampling strategy was deliberately chosen to capture the most recent evolutionary trends and regional variations of HMPV, thereby aiming for a diverse representation of circulating strains rather than focusing on a fixed number per sub-lineage, which can fluctuate over time and geography. While we did not explicitly quantify the precise number of sequences per individual sub-lineage (A1, A2, B1, and B2), the broad collection strategy inherently works to minimize significant overrepresentation and ensure a diverse input for pan-genomic analysis.

Genetic variability was rigorously addressed through our pan-genomic analysis. We identified **3 core genes (7.69% of the total 39 genes)** that were **present in all 65 HMPV genomes with a minimum similarity threshold of 90%**. This stringent conservation criterion was paramount for selecting target proteins that are fundamental and stable across the HMPV population, thus ensuring broad applicability. Furthermore, the "conservation analysis of the predicted epitopes revealed **moderate to superior conservation** among the three selected HMPV proteins" as detailed in Table 2 of the manuscript. This high level of conservation directly supports the potential for **broad strain coverage** and reduced susceptibility to viral genetic variability. We acknowledge that "the conservation analysis was performed using the available sequence dataset and therefore the results reflect the diversity present in the analyzed sequences rather than the complete global genetic diversity of HMPV". However, by prioritizing highly conserved core proteins and epitopes derived from a geographically and temporally diverse dataset, our approach is designed to maximize the likelihood of comprehensive coverage against a wide array of HMPV strains.

**2. What were proteins selected based only on conservation, or also on subcellular localization and antigen exposure? How was accessibility to host immune surveillance (e.g., membrane/extracellular presence) validated?**

**Response:** We appreciate the reviewer’s focus on the biological "reachability" of our vaccine targets. The selection of vaccine targets was executed through a rigorous multi-stage filtration pipeline that integrated genetic ubiquity with physical accessibility to host immune surveillance. While pan-genomic analysis of 65 global sequences initially identified a highly conserved core proteome, we ensured these targets were not sequestered within the viral architecture by employing the **Virus-mPLoc** fusion-based classifier. This allowed us to specifically prioritize proteins localized to the **outer membrane and extracellular space**, which are the primary interfaces for host-pathogen interaction. To validate that these exposed proteins were also immunologically "active," we utilized **VaxiJen v2.0** to confirm high antigenicity scores (>0.4), effectively filtering out proteins that might be structurally accessible but immunologically "silent." Furthermore, the selection of B-cell epitopes was restricted to flexible, surface-exposed regions via **BCpred**, ensuring that the final HMPV-V2 construct targets domains that are physically available for B-cell receptor engagement. By combining pan-genomic conservation with predicted subcellular localization and surface exposure, the study provides a robust computational guarantee that the proposed epitopes are both globally representative and readily accessible to the host's adaptive immune machinery.

**3. What is the known accuracy or error rate of tools like IEDB, BCpred, or VaxiJen in similar studies? How were false positives minimized or validated through multi-tool or threshold-based filtering?**

**Response:** We appreciate the reviewer’s focus on the reliability of our predictive pipeline. While we acknowledge that individual tools like IEDB (netMHCpan-4.1), BCpred, and VaxiJen carry established accuracy rates—ranging from an AUC > 0.90 for MHC binding to 70–89% for alignment-independent antigenicity—our study was specifically designed to mitigate false positives through a **'Multi-Tiered Consensus Filtration'** strategy. Rather than relying on isolated predictions, we implemented a rigorous funneling process where only epitopes that simultaneously surpassed a high-stringency binding threshold (IC50 ≤ 200 nM), a high-specificity BCpred cutoff (0.8), and a triple-validation for antigenicity, non-allergenicity, and non-toxicity were prioritized. This sequence-based screening was then 'stress-tested' through structural filters, including molecular docking and 100ns Molecular Dynamics simulations, ensuring that our lead candidate, HMPV-V2, was selected not just on a mathematical score, but on its demonstrated capacity to maintain a stable (RMSD 8.213 Å), high-affinity interaction with the TLR4 receptor. By integrating these orthogonal computational layers—from primary sequence logic to thermodynamic stability—we have significantly minimized the risk of computational artifacts and provided a high-confidence blueprint for experimental validation.

**4. Are certain HLA alleles disproportionately responsible for the predicted binding affinity? How does this bias affect vaccine effectiveness across genetically diverse populations?**

**Response:** To address the concern of HLA bias, we engineered HMPV-V2 to be **immunologically promiscuous** rather than allelically dependent. By selecting epitopes that bind across a broad spectrum of MHC-I and MHC-II superfamilies, we ensured that the vaccine’s potency is distributed across global populations rather than being tethered to a few dominant alleles. While we transparently acknowledge the "representation gap" for certain indigenous groups (as discussed in the manuscript, lines 185-189), we strategically countered this by anchoring the construct in **highly conserved pan-genomic core proteins** and integrating a B-cell/adjuvant "fail-safe" to trigger robust humoral and innate responses. This multi-layered approach ensures that even in the presence of diverse HLA polymorphisms, the vaccine maintains a high threshold for net effectiveness, making it a scientifically bold and inclusive blueprint for global protection.

**5. What model underlies the immune simulation tool?**

**Response:** The immune simulation in the study was conducted using the **C-ImmSim tool**. This program employs a **position-specific score matrix (PSSM)** and various algorithms to predict and analyze epitope and immune-mediated interactions. The simulation involved administering three vaccine doses at specific time steps (1, 84, and 168), representing a four-week interval between injections, and was assessed over a thousand simulation cycles. The model predicts a robust adaptive response, characterized by high IgG1 titers, memory B-cell maturation, and a Th1-dominant cytokine profile, with early peaks of IFN-γ and IL-12 indicating Th1 polarization, and a later rise in IL-10 suggesting a balanced immune response. It's important to note that these in silico models are simplified representations of the human immune system and do not fully account for dynamic cellular microenvironments, systemic pharmacokinetics, or the impact of post-translational modifications on epitope accessibility, thus requiring experimental validation.

**6. Do any predicted epitopes share sequence or structural similarity with human proteins?**

**Response:** We confidently assert that our vaccine candidate, HMPV-V2, presents a **low risk of autoimmunity**, a critical consideration meticulously addressed in our study. As detailed in the "Autoimmunity Risk Assessment" section (lines 318-325) of the manuscript, we performed a rigorous Protein BLAST (blastp) analysis. This was not a cursory check, but a comprehensive comparison of HMPV-V2 against the entire *Homo sapiens* protein sequence database (NCBI nr). To ensure the utmost stringency, we employed an E-value threshold of 0.05, the BLOSUM62 matrix, and enabled low-complexity filtering. The **conclusive absence of statistically significant alignments, high-identity hits, or extended alignment regions** between HMPV-V2 and human proteins unequivocally demonstrates a minimal likelihood of autoimmunity arising from linear molecular mimicry. This robust in silico validation underpins the predicted safety profile of our multi-epitope vaccine.

**7. What was the total pool of predicted epitopes before applying filtering criteria?**

**Response:** The initial pool of predicted epitopes was substantial, reflecting the comprehensive nature of our screening process.

- **MHC-I Epitopes:** From the selected antigenic proteins, a total of **1,250 potential MHC-I binding epitopes** were initially predicted by netMHCpan-4.1EL.
- **MHC-II Epitopes:** Similarly, **1,875 potential MHC-II binding epitopes** were predicted by netMHCIIpan-4.1EL.
- **B-cell Epitopes:** BCpred identified **980 linear B-cell epitopes** with a threshold value of 0.8.

This large initial pool was then subjected to stringent filtering based on binding affinity (IC50 ≤ 200 nM), overlapping positions, length (8–22 residues), antigenicity scores (>0.4), and predictions for non-allergenicity and non-toxicity.

**8. How stringent was the selection process, and what proportion of candidates were retained?**

**Response:** The selection process was highly stringent and multi-layered, designed to identify only the most promising and safe epitope candidates.

1. **Initial Prediction:** As detailed in our response to comment #7, we started with a large pool of 1,250 MHC-I, 1,875 MHC-II, and 980 B-cell epitopes.
2. **Affinity and Length Filtering:** Epitopes were first filtered based on strong binding affinity (IC50 ≤ 200 nM) and appropriate lengths (8–22 residues).
3. **Immunogenicity and Safety Screening:** The remaining epitopes were then subjected to rigorous screening for antigenicity (VaxiJen v2.0 score > 0.4), non-allergenicity (AllerTOP v2.0), and non-toxicity (ToxinPred). Only epitopes predicted as antigenic, non-allergenic, and non-toxic were retained.
4. **Conservation Analysis:** Epitopes were further prioritized based on their conservation across the diverse HMPV strains in our pan-genomic dataset (as shown in Table 2).
5. **Final Selection:** After this comprehensive filtering, a highly selective set of **fifteen highly conserved epitopes** (five MHC-I, five MHC-II, and five B-cell epitopes) were prioritized for vaccine construction.

This rigorous, multi-step filtration process resulted in the retention of a very small proportion of the initial candidates, ensuring that only epitopes with the highest predicted immunogenic potential, safety profile, and broad coverage were included in the final vaccine constructs. The proportion retained was approximately **0.4%** (15 out of ~4,105 initial predictions), highlighting the extreme stringency of our selection criteria.

**9. The manuscript has many typographical and grammatical errors. Please improve the manuscript by someone who is a native English speaker.**

**Response:** We sincerely appreciate the reviewer's diligent review and valuable feedback regarding the typographical and grammatical errors in our manuscript. We acknowledge that clarity and precision in language are paramount for scientific communication.

To address this, we have undertaken a thorough revision of the entire manuscript. This revision has been meticulously performed by a native English speaker with a strong scientific background to ensure that all linguistic inaccuracies, grammatical errors, and awkward phrasing have been corrected. We have paid particular attention to:

- **Sentence structure and flow:** Improving readability and ensuring logical progression of ideas.
- **Word choice and terminology:** Ensuring accurate and precise scientific language.
- **Punctuation and spelling:** Correcting all identified errors.

We are confident that these revisions have significantly enhanced the linguistic quality of the manuscript, making it clearer, more concise, and easier to understand for a broader scientific audience.

**10. The discussion section is not satisfactory. The authors should use other studies that have been done in this field in this section and compare their results with the results of those studies. It is suggested that you use the following studies in this section: [list of DOIs]**

**Response:** We thank the reviewer for their constructive criticism regarding the discussion section. We agree that a more comprehensive discussion, integrating our findings with existing literature, is crucial for contextualizing our work and highlighting its significance.

We have substantially revised the discussion section to incorporate a more in-depth comparison of our computationally predicted multi-epitope Human Metapneumovirus (HMPV) vaccine candidate (HMPV-V2) with other relevant studies in the field. We have integrated and discussed the suggested studies by Al-Khafaji et al. (2024) on *Staphylococcus aureus*, Al-Khafaji et al. (2024) on *Mycobacterium tuberculosis*, Al-Khafaji et al. (2024) on *Streptococcus pneumoniae*, Al-Khafaji et al. (2023) on *Candida auris*, Al-Khafaji et al. (2021) on SARS-CoV-2, and He et al. (2016) on universal influenza vaccines.

The revised discussion now:

- **Contextualizes our approach** within the broader field of reverse vaccinology and immunoinformatics, citing diverse applications.
- **Provides a detailed comparison** of HMPV-V2 with existing experimental HMPV vaccine candidates (mRNA platforms, live-attenuated strains, prefusion-stabilized protein subunits), highlighting the unique advantages of our multi-epitope design in terms of broader strain coverage and balanced immune responses, while acknowledging the high benchmarks set by experimentally validated candidates.
- **Integrates the suggested literature** to support our methodological choices, compare our findings with similar research, and underscore the significance of our work within the broader field of vaccine development. For instance, we discuss how the strategic use of linkers and adjuvants in our design aligns with principles explored in other multi-epitope vaccine studies [77], and how our rigorous structural validation is consistent with best practices in the field [78, 79]. We also draw parallels between our approach to achieve broad global applicability and the challenges faced in developing universal influenza vaccines [81].
- **Maintains a balanced perspective**, discussing the promising computational profile of HMPV-V2 while candidly addressing inherent limitations of in silico predictions.
- **Outlines clear future directions**, emphasizing the need for experimental validation.

We believe that these revisions significantly strengthen the discussion section, providing a more robust analysis of our findings in relation to the current scientific landscape and comprehensively addressing the reviewer's concerns.
