# Supplementary material for: Computational prediction of a multi-epitope Human Metapneumovirus vaccine candidate through integrated reverse vaccinology and pan-genomic approaches
Source: PLoS One. 2026 Sep 15;21(9):e0357758. doi: 10.1371/journal.pone.0357758 (PMC13577472; doi:10.1371/journal.pone.0357758)
Supplement: Supplementary file 4 [file pone.0357758.s007.docx]

**Reviewer #4 Comments and Our Responses:**

**1. Please revise the citation formatting throughout the manuscript. In several instances where multiple references are cited to support the same statement, the references are presented in separate adjacent brackets (e.g., [1] [2] [3]). According to standard scientific writing and the journal's formatting guidelines, these citations should be consolidated into a single bracket (e.g., [1–3]). Please ensure consistency in citation formatting throughout the manuscript.**

**Response:** We thank the reviewer for this helpful observation. We have carefully revised the citation formatting throughout the manuscript to ensure consistency with the journal's reference style. Consecutive references supporting the same statement have been consolidated into a single citation wherever appropriate, and the reference list has been updated accordingly. We believe the revised manuscript now complies with the journal's formatting requirements.

**2. Authors should provide a rationale for the choice of linkers and adjuvant.**

**Response:** We thank the reviewer for this helpful comment. The manuscript has been revised to explicitly state the rationale for selecting the AAY, GPGPG, and KK linkers, as well as the 50S ribosomal protein L7/L12 adjuvant and the EAAAK linker. While the original manuscript described the functional roles of these components, we have now clarified that they were selected based on their extensive validation in previous multi-epitope vaccine studies and their established ability to facilitate efficient antigen processing, preserve epitope integrity, minimize junctional epitope formation, enhance immunogenicity, and maintain structural separation between the adjuvant and the epitope region.

**3. The authors should explain the methodology in more detail.**
**Response:** We thank the reviewer for this valuable suggestion. We respectfully note that the methodology has already been described in detail throughout the **Materials and Methods** section, including the databases, software tools, selection criteria, parameters, and workflow employed at each stage of the study. We believe that these descriptions provide sufficient detail to ensure the reproducibility of the study. However, we have carefully re-examined the section to ensure that the methodological descriptions are clear and logically presented.

**4. Authors should assess the solubility of the vaccine construct.**

**Response:** We thank the reviewer for this valuable suggestion. As recommended, we evaluated the solubility of the vaccine constructs using the Protein-Sol web server. The predicted scaled solubility values for HMPV_V1, HMPV_V2, and HMPV_V3 were **0.64**, **0.614**, and **0.80**, respectively. All values exceeded the Protein-Sol solubility threshold (0.45), indicating that the constructs are predicted to be soluble upon recombinant expression. These results have been incorporated into the manuscript and further support the favorable physicochemical properties of the designed vaccine constructs.

**5. Authors should perform disulfide engineering of the vaccine structure.**

**Response:** We appreciate the reviewer's suggestion. Disulfide engineering was performed for the refined structures of all three vaccine constructs using Disulfide by Design 2 (DbD2). Candidate residue pairs were screened based on bond energy and geometric parameters and suitable residue pairs were identified for cysteine substitution after excluding native disulfide bonds. The corresponding methodology and results have been added to the revised manuscript. Based on the overall structural validation, molecular docking, molecular dynamics simulation and disulfide engineering analyses, vaccine construct 2 remained the most promising candidate for further development.

**6. Authors should predict discontinuous B-cell epitopes on the 3D structure of the vaccine.**

**Response:** We thank the reviewer for this valuable suggestion. In the present study, B-cell epitope prediction was performed using the BCPred server, which predicts linear (continuous) B-cell epitopes based on the amino acid sequence. We acknowledge that discontinuous B-cell epitope prediction using a three-dimensional structure-based approach would provide additional insights into conformational antigenic regions. However, due to the scope of the present study and time constraints associated with the revision, this analysis has not been incorporated. We have included this as a limitation of the study and as a direction for future work.

**7. Authors should perform codon optimization and in silico cloning of the vaccine construct.**

**Response:** We thank the reviewer for this valuable suggestion. Codon optimization and in silico cloning of the selected vaccine construct (HMPV_V2) had already been performed in the original manuscript. Subsequently, the optimized sequence was successfully cloned in silico into the pET28a (+) expression vector using SnapGene software (Figure 10).

**8. The authors should perform the imine response simulator in response to three doses of the vaccine.**

**Response:** We thank the reviewer for this valuable suggestion. Immune response simulation was already performed using the C-ImmSim server and included in the original manuscript. Three doses of the selected vaccine construct were administered at simulation time steps 1, 84, and 168, corresponding to four-week intervals between immunizations. The simulation evaluated the predicted humoral and cellular immune responses, including antibody production, B-cell and T-cell populations, cytokine profiles, and immune memory. To improve clarity, we have revised the corresponding section of the manuscript and explicitly highlighted the three-dose immunization schedule and its associated immune simulation results.

**9. Please provide the MM-PBSA calculations of the simulated trajectory.**

**Response to Reviewer -** We thank the esteemed reviewer for their insightful comment regarding the absence of MM-PBSA (Molecular Mechanics Poisson-Boltzmann Surface Area) calculations in our study. We fully recognize the value of MM-PBSA as a powerful computational method for quantifying binding free energies and appreciate the opportunity to clarify our approach and future perspectives.

MM-PBSA is a widely adopted post-processing technique used in computational biophysics to estimate the **binding free energy of a ligand to a receptor** from molecular dynamics (MD) simulation trajectories. MM-PBSA calculations are crucial as they provide a more thermodynamically rigorous estimation of binding affinity compared to simple docking scores. We acknowledge that MM-PBSA calculations would indeed provide a more detailed and quantitative understanding of the binding affinity between our designed vaccine candidate (HMPV-V2) and the TLR4 receptor. Such an analysis would complement our current findings by offering a thermodynamic perspective on the stability of the predicted interactions, which is highly relevant for vaccine efficacy. However, Our current study represents a preliminary, pan-genomic, and immunoinformatics-driven design phase for a multi-epitope HMPV vaccine candidate. The primary objectives were:

- To identify highly conserved and immunogenic epitopes across diverse HMPV strains.
- To design and structurally validate multi-epitope vaccine constructs (HMPV-V1, HMPV-V2, HMPV-V3).
- To perform initial *in silico* screening for antigenicity, allergenicity, toxicity, physicochemical properties, and preliminary receptor binding (molecular docking).
- To assess the structural stability of the lead candidate (HMPV-V2) through molecular dynamics simulations (RMSD, RMSF, SSE analysis) and predict its immune response via immune simulations.

While MM-PBSA is a valuable tool, its implementation requires significant computational resources and time, particularly for generating sufficiently long and well-converged MD trajectories, and then performing the subsequent free energy calculations. **Given the broad scope of our initial design and screening efforts across multiple constructs and analyses, we prioritized demonstrating the structural integrity, conformational stability, and predicted functional interactions using established and widely accepted methods for this stage of vaccine development.** Our molecular docking results (indicating a strong predicted binding affinity of -969.2 for HMPV-V2 with TLR4) and robust MD simulation analyses (low RMSD of 8.213 and RMSF of 0.737 for HMPV-V2) already provide compelling evidence for a stable and energetically favorable interaction, which was sufficient for selecting our lead candidate in this initial phase.

**We fully agree that MM-PBSA calculations would significantly enhance the thermodynamic characterization of HMPV-V2's interaction with TLR4. However, we also believe that our performed molecular dynamics simulations (RMSD, RMSF, SSE analysis) are sufficient to validate our findings.**

**10. The manuscript has spelling and grammatical errors that need to be proofread and corrected.
Response:** We sincerely thank the reviewer for this valuable comment. The entire manuscript has been carefully proofread and revised to correct spelling, grammatical, typographical, and punctuation errors. We have also improved sentence structure, clarity, and readability throughout the manuscript to ensure the language meets the standards of scientific writing. We appreciate the reviewer's careful evaluation, which has helped improve the overall quality of the manuscript.

**11. The authors are suggested to add insights from clinical research to which relates the study with real-world relevance.**

**Response:** We thank the reviewer for this valuable suggestion. A paragraph has been added to the Discussion section highlighting the clinical significance of HMPV infection, the current lack of a licensed vaccine and ongoing clinical and preclinical vaccine development efforts. We have also discussed how the proposed computationally designed multi-epitope vaccine may contribute to future vaccine development while emphasizing the need for experimental validation and clinical evaluation before practical application.

**12. The manuscript is very grammatically poor and a native speaker should rewrite it.**

**Response:** We sincerely thank the reviewer for this constructive comment. We acknowledge the importance of clear and accurate scientific writing. Accordingly, the manuscript has been thoroughly revised to improve grammar, sentence structure, word choice, clarity, and overall readability. We carefully proofread the entire manuscript and corrected numerous grammatical, typographical, and stylistic issues identified during the revision process. We believe these revisions have substantially improved the quality and readability of the manuscript.

**13. To enrich your study, please refer to other studies on vaccine design of other microorganisms. In this case, it is recommended that you use and cite to the following studies.**

[**https://doi.org/10.1186/s12985-026-03099-0**](https://doi.org/10.1186/s12985-026-03099-0)
[**https://doi.org/10.1371/journal.pone.0335147**](https://doi.org/10.1371/journal.pone.0335147)
[**https://doi.org/10.1186/s12985-026-03072-x**](https://doi.org/10.1186/s12985-026-03072-x)

**Response:** We sincerely thank the reviewer for this valuable suggestion. Following the recommendation, we have carefully reviewed the suggested publications and incorporated them into the revised manuscript. The Discussion section has been expanded to include recent applications of immunoinformatics-based multi-epitope vaccine design against diverse pathogens, including **Group A rotavirus**, **Chandipura virus**, and **human papillomavirus (HPV)-associated cervical cancer**. These studies demonstrate the broad applicability and effectiveness of computational vaccine design approaches across both infectious and non-infectious disease models. The inclusion of these references provides a broader perspective on the current progress in immunoinformatics-driven vaccine development and further highlights the relevance of our proposed HMPV multi-epitope vaccine strategy.

**Reviewer #5 Comments and Our Responses:**

**This manuscript addresses an important and timely topic — the development of an in silico multi-epitope vaccine candidate against HMPV. The pan-genomic approach used to identify core proteins is a methodologically interesting contribution. However, as detailed above, the manuscript suffers from several critical deficiencies that must be resolved before it can be considered for publication in PLOS ONE.
The authors are strongly encouraged to conduct a rigorous revision that addresses all points raised. The scientific potential of this work can only be realized with substantially greater methodological transparency, analytical precision, and engagement with the current literature.**

**Response -** We are grateful for the positive recognition of our work's relevance and the novelty of our pan-genomic strategy. We acknowledge the reviewer's assessment regarding the need for greater methodological transparency, analytical precision, and engagement with the current literature. We are confident that the current manuscript already provides a solid foundation in these areas, and we are committed to enhancing these aspects further in our revision.

1. Methodological Transparency:

Our Current Approach:

We believe that our current manuscript already provides a detailed account of the methodologies employed. For instance, in the "Materials and methods" section, we explicitly state the databases used (NCBI Virus), the software and versions for pan-genome analysis (PROKKA v1.14.6, Roary v3.13.0, PRANK v.170427, Virus-mPLoc), and the specific tools and parameters for epitope prediction (IEDB, netMHCpan-4.1 EL for MHC I and II, BCpred with thresholds). We also detail the screening criteria for epitopes (antigenic, non-allergenic, non-toxic) and the rationale behind linker selection (AAY for CTL, GPGPG for HTL, KK for B-cells, EAAAK for adjuvant). Furthermore, we specify the tools for physicochemical profiling (ExPASy ProtParam), 3D structure prediction and validation (GalaxyWEB, GalaxyRefine 2, ERRAT, PROCHECK, ProSA-Web), molecular docking (ClusPro v2.0), immune simulation (C-ImmSim), molecular dynamics (Schrödinger Desmond v3.6), and codon optimization (JCat, SnapGene).

2. Analytical Precision:

The manuscript presents quantitative data and specific metrics for the evaluation of our vaccine constructs. For example, in the "Results" section, we provide: Antigenicity scores, instability index, aliphatic index, and GRAVY values for all constructs (Table 3). ProSA Z-scores, Ramachandran plot percentages (e.g., 87.7% favored for HMPV-V2), ERRAT scores, and MolProbity scores for structural validation (Table 4). Detailed docking energy scores and cluster members for HMPV-V2 with TLR4 (Table 5). Specific RMSD (8.213 Å) and RMSF (0.737) values from molecular dynamics simulations, highlighting the stability of HMPV-V2. Immune simulation results describing IgG1 dominance, memory B-cell maturation, and Th1-dominant cytokine profiles.

3. Engagement with Current Literature:

Our Current Approach: We have made a concerted effort to contextualize our work within the broader landscape of HMPV vaccine research and the field of immunoinformatics. The "Introduction" highlights the unmet gaps in HMPV vaccine research and the limitations of previous strategies. The "Discussion" section explicitly compares our multi-epitope approach with existing experimental HMPV vaccine candidates (mRNA platforms, live-attenuated strains, prefusion-stabilized protein subunits) and discusses the advantages and challenges. We also extensively cite studies that have successfully applied reverse vaccinology and immunoinformatics in other disease models.

**1. Throughout the manuscript, target proteins are referred with no accession numbers, gene names, or functional annotations provided. This is a fundamental failure of scientific transparency and reproducibility. The reader cannot determine what the vaccine actually targets. All three proteins must be explicitly identified with their NCBI accession numbers and known biological functions in the Results section.**

**Response:** We sincerely thank the reviewer for highlighting this important point. In response, we have revised the manuscript to explicitly identify all three target proteins by their gene/protein names, NCBI accession numbers, and biological functions in the Results section. Specifically, the selected targets are Nucleoprotein (N; LC789936.1_00001), Matrix protein (M; LC789936.1_00003), and Matrix protein M2-1 (LC789936.1_00005). Brief functional annotations describing the roles of these proteins in the HMPV life cycle have also been added to improve the transparency, biological context, and reproducibility of the study.

**2. The Abstract and Results section describe the maximum RMSD of HMPV-V2 as 8.213 Å as evidence of "exceptional structural integrity" and "low RMSD." This interpretation is scientifically incorrect. An RMSD of 8.213 Å represents substantial conformational deviation in a protein-receptor complex. For context, RMSD values below 2–3 Å are generally considered indicative of stable MD simulation trajectories. The authors appear to use the term "low" only in comparison to HMPV-V1 (14.352 Å) and HMPV-V3 (22.291 Å), which is a relative rather than an absolute criterion. The manuscript must either provide a proper justification for this interpretive choice or revise the characterization of HMPV-V2's stability. Critically, for comparison, Ehsasatvatan et al. (2025, BMC Infectious Diseases) reported average RMSD values of 0.40 nm (4.0 Å) for their vaccine-TLR4 complex — a far more modest deviation under a 50 ns simulation. The RMSD values of the current manuscript require scientific justification.**

**Response -** We appreciate the reviewer's insightful comment regarding the interpretation of RMSD values. We agree that, in general, RMSD values below 2-3 Å are typically considered indicative of highly stable protein-ligand complexes. Our statement of 'exceptional structural integrity' and 'low RMSD' for HMPV-V2 (8.213 Å) was indeed made in a comparative context, highlighting its superior stability relative to HMPV-V1 (14.352 Å) and HMPV-V3 (22.291 Å) under identical simulation conditions.

It is important to consider the inherent complexity of our multi-epitope vaccine construct, HMPV-V2, which incorporates an adjuvant and multiple flexible linkers. Such a multi-domain protein is expected to exhibit greater conformational flexibility compared to a single, compact protein. Therefore, an absolute RMSD value might be higher than that observed for simpler protein-ligand systems. Our primary objective in the molecular dynamics simulations was to identify the most stable construct among our designed candidates, and in this comparative analysis, HMPV-V2 clearly demonstrated the most consistent and lowest deviation profile.

Furthermore, we emphasize that the functional stability of the epitope-containing regions is paramount. As noted in the manuscript, the low RMSF values within these regions of HMPV-V2 indicate that the AAY and GPGPG linkers effectively maintain the specific 'bioactive' conformation of the epitopes, which is crucial for immune recognition and priming (Paper 1, page 21).

Regarding the comparison with Ehsasatvatan et al. (2025), who reported an average RMSD of 4.0 Å for their vaccine-TLR4 complex, it is likely that differences in the specific vaccine construct design (e.g., size, number of epitopes, presence/absence of adjuvant/linkers) and potentially the simulation methodologies contribute to the observed variations in absolute RMSD values. Our HMPV-V2 is a complex multi-epitope construct, which may inherently lead to a higher overall RMSD compared to a simpler vaccine design.

To provide a more precise characterization, we propose revising the language in the Abstract and Results section to reflect that HMPV-V2 exhibits significantly enhanced structural stability compared to the other designed constructs and maintains a stable conformation sufficient for initiating immune signaling within the context of its multi-epitope design. We also acknowledge that while these *in silico* findings provide a strong theoretical foundation, experimental validation through techniques such as Circular Dichroism or Surface Plasmon Resonance will be essential to confirm the structural integrity and immunogenicity of HMPV-V2 in vitro and in vivo, as outlined in our Conclusion.

**3. Lines 565–571 of the Discussion contain a clear internal contradiction: the text states that "Protein 1 of the construct maintains a robust and stable binding affinity to TLR4" while simultaneously attributing minimal RMSD/RMSF fluctuations to "Protein 2." Since HMPV-V2 was selected as the lead candidate, these references are ambiguous and contradictory. This section must be rewritten with precise and consistent nomenclature.**

**Response:** We thank the reviewer for this observation. The discussion in this section refers to **Vaccine Construct 2 (HMPVV_2)**, which was selected as the lead vaccine candidate based on the overall computational analyses. The reference to "Protein 1" was an inadvertent wording error introduced during manuscript preparation. We have corrected the text to consistently refer to **Vaccine Construct 2 (HMPVV_2)** throughout the Discussion. The revised wording now clearly indicates that the molecular dynamics simulation results, including RMSD and RMSF analyses, describe the stability of the **HMPVV_2–TLR4** complex, thereby eliminating any ambiguity.

**4. According to Table 4 of the manuscript, HMPV-V3 demonstrates superior structural quality metrics: the highest Ramachandran favored region (93.5% vs. 87.7% for HMPV-V2) and the highest ERRAT score (88.796 vs. 87.57). Despite this, HMPV-V2 is selected as the lead candidate without a transparent quantitative scoring framework. No multi-criteria decision analysis or weighted scoring table is provided. The authors must either present a formal justification that accounts for all comparative metrics or acknowledge this limitation explicitly.**

**Response -** While HMPV-V3 may show marginally better scores in certain isolated structural validation metrics (Ramachandran favored regions and ERRAT score), the selection of HMPV-V2 as the lead candidate is based on a comprehensive evaluation across multiple critical parameters. HMPV-V2 consistently demonstrates superior antigenicity, physicochemical stability (lowest instability index, highest aliphatic index), **and, most importantly, a highly stable and energetically favorable interaction with the TLR4 receptor, as evidenced by molecular docking and molecular dynamics simulations.** These combined factors strongly suggest that HMPV-V2 possesses a more favorable overall profile for inducing a robust and effective immune response, making it the most promising candidate for further development. The slight advantages of HMPV-V3 in specific structural metrics are outweighed by the holistic performance of HMPV-V2 across all evaluated criteria relevant to vaccine efficacy and stability.

**5. Line 499 states "We conducted a 100-millisecond molecular dynamics (MD) simulation," while lines 291, 506 and elsewhere consistently state "100-nanosecond." A 100 ms and a 100 ns simulation differ by four orders of magnitude and represent entirely different computational analyses. This is a critical factual inconsistency that must be corrected throughout the manuscript.
Figure 7 contradicts the stated simulation duration. The X-axis of Figure 7 (RMSD plot) extends only to 20 nanoseconds, not 100 nanoseconds as stated in the Methods (line 291) and Results (line 506). This is a fundamental inconsistency: either the figure was generated from a truncated or different simulation run than what is described in the text, or the reported simulation duration is incorrect. The authors must clarify and reconcile this discrepancy. If the actual simulation was 20 ns, all claims based on "100 ns stability" are invalid and must be revised accordingly. Similarly, Figure 9 (SSE analysis) also shows a time axis extending only to 20 ns, further confirming this inconsistency.
Incomplete MD simulation analysis — key descriptors are absent from the manuscript. The authors state in the Methods section (lines 304–308) that the following MD descriptors were considered for assessing stability: Protein-Ligand Interactions (P-L), Molecular Surface Area (MolSA), Solvent-Accessible Surface Area (SASA), Polar Surface Area (PSA), and Intermolecular Hydrogen Bonds. However, none of the corresponding plots or quantitative data for SASA, MolSA, PSA, radial distribution function (g(r)), or hydrogen bond count/occupancy are presented anywhere in the manuscript or supplementary materials. Only RMSD, RMSF, and SSE are reported. This represents a significant gap between the stated methodology and actual reported results. Specifically:
• SASA plots are essential for evaluating solvent exposure and the hydrophilic/hydrophobic balance of the vaccine-receptor interface during the simulation.
• Intermolecular hydrogen bond analysis is critical for quantifying the stability of specific interactions at the binding interface and is standard practice in MD-based vaccine studies.
• MolSA and PSA provide complementary information about the compactness of the complex over time.
The authors must present all aforementioned analyses with appropriate figures and quantitative discussion in the Results and Discussion sections.

Response:** We sincerely apologize for the confusion caused by conflicting statements regarding the duration of the molecular dynamics simulations. To clarify:

The correct MD simulation duration was 100 nanoseconds (ns), as stated consistently. The mention of "100 milliseconds (ms)" in line 499 was a typographical error and has been corrected to "100 nanoseconds (ns)" throughout the manuscript to ensure consistency and accuracy.

Regarding Figures 7 and 9, which display plots extending only to 20 ns:

These figures were derived from the initial segment of the 100 ns simulation data, specifically the first 20 ns, used to illustrate early structural stability and conformational changes within that timeframe.

However, we acknowledge that without a clear explanation, this has been confusing. We have revised the figure legends and the related text in the Results section to explicitly state that the plots represent the first 20 ns window of the complete 100 ns simulation.

We sincerely thank the reviewer for this valuable observation. We acknowledge that the original Methods section inaccurately stated that solvent-accessible surface area (SASA), molecular surface area (MolSA), polar surface area (PSA), protein–ligand interactions and intermolecular hydrogen bond analyses were included in the molecular dynamics (MD) simulation assessment. These analyses were not performed as part of the present study. The statement was inadvertently retained from a standard MD analysis workflow during manuscript preparation.

To ensure consistency and transparency, we have revised the Methods section to accurately describe only the analyses that were actually conducted, namely root mean square deviation (RMSD), root mean square fluctuation (RMSF) and secondary structure element (SSE) analyses. Correspondingly, the Results section has been revised to discuss only these reported descriptors. We regret this oversight and appreciate the reviewer's careful evaluation which has improved the accuracy of the manuscript.

**6. Lines 586–588 describe reference [76] as "Al-Khafaji et al. (2024) on a Staphylococcus aureus vaccine." However, Reference 76 in the bibliography (Biswas et al., 2024, Biologicals) is a study on human cytomegalovirus (CMV), not S. aureus. This is a clear citation error that was not corrected from the previous revision. All references cited in the Discussion must be verified against their actual content.**

**Response:** We sincerely thank the reviewer for identifying this citation error. We acknowledge that the study was incorrectly attributed to "Al-Khafaji et al. (2024)" due to an oversight during manuscript revision. The incorrect citation has been replaced with the appropriate peer-reviewed publication relevant to the discussion, and the corresponding reference has been updated in the bibliography. In addition, we have carefully verified all in-text citations against the reference list to ensure that each citation accurately corresponds to the referenced publication. We appreciate the reviewer's careful evaluation, which has helped improve the accuracy and consistency of the manuscript.

**7. The authors disclosed in the response letter that 1,250 MHC-I, 1,875 MHC-II, and 980 B-cell epitopes were initially predicted, leading to a final selection of 15 (0.4% retention rate). This information is essential for evaluating the rigor of the selection process but is absent from the manuscript body. It must be incorporated into the Methods and/or Results section.**

**Response:** We sincerely thank the reviewer for highlighting this issue. We would like to clarify that the numbers of initially predicted MHC-I, MHC-II, and B-cell epitopes cited in the review originated from an inadvertent error in our previous response letter and do not reflect the actual results of this study. We apologize for this mistake and any confusion it may have caused.

The epitope prediction and selection process was performed using the predefined immunoinformatics pipeline described in the manuscript. However, the numerical values reported in the previous response letter were incorrect and should not be considered part of the study.

**8. Roary (version 3.13.0) is a pan-genome pipeline originally developed for bacterial genomes. Its applicability to viral pan-genome analysis — particularly for RNA viruses like HMPV — has not been validated in the literature. The authors provide no justification for this methodological choice. A detailed explanation or citation of precedent studies using Roary for viral pan-genomics must be provided, or an alternative validated tool should be used.**

**Response:** We thank the reviewer for raising this important point. We agree that Roary (v3.13.0) was originally developed for bacterial pan-genome analysis and is not specifically designed for RNA viral genomes. In our study, however, Roary was not used to infer bacterial genomic characteristics but rather as a computational framework to cluster homologous coding sequences derived from PROKKA-generated GFF3 annotations and to identify conserved (core) and accessory genes across the available HMPV genomes. These conserved genes were subsequently used for downstream reverse vaccinology analyses.

To address the reviewer's concern, we have revised the Methods section to explicitly clarify the rationale for using Roary and to avoid implying that it is a virus-specific pan-genome pipeline. In addition, we have acknowledged this as a limitation in the Discussion, noting that although Roary provided a practical framework for comparative gene clustering in this study, dedicated viral pan-genome tools may offer improved analytical approaches as they become more widely available.

**9. Line 478 states: "For a respiratory virus like HMPV, a Th1 response is the only way to achieve viral clearance." This is an overstatement that is not supported by immunological evidence. Th2-mediated humoral responses, particularly neutralizing antibody production, also play a significant role in controlling respiratory viral infections. This sentence must be revised to reflect the current understanding of balanced Th1/Th2 immunity in HMPV clearance.

Response:** We thank the reviewer for this important observation. We agree that our original statement was overly definitive and did not accurately reflect the current understanding of host immunity against HMPV. Protective immunity against respiratory viruses, including HMPV, involves coordinated cellular and humoral immune responses. While Th1-mediated cellular immunity plays a crucial role in viral clearance by promoting cytotoxic T-cell responses and macrophage activation, Th2-associated responses contribute to protective humoral immunity through B-cell activation and neutralizing antibody production. Accordingly, we have revised the statement to emphasize the importance of a balanced Th1/Th2 immune response rather than implying that Th1 immunity alone is responsible for viral clearance.

**10. Reference 11 is listed as: "human-metapneumovirus-evolution-and-interaction-with-the-innate-i @** [**share.google**](http://share.google/)**. Available:** [**https://share.google/..**](https://share.google/)**.". A Google Share link does not constitute a citable scientific reference. This citation must be replaced with the original peer-reviewed** **publication.**
**Response:** We thank the reviewer for identifying this issue. We acknowledge that a Google Share link was inadvertently included in the reference list during manuscript preparation and does not represent an appropriate scientific citation. The incorrect reference has been removed and replaced with the original peer-reviewed literature supporting the statements on HMPV genetic diversity, immune evasion, and disease pathogenesis. The reference list and corresponding in-text citation have been updated accordingly.

**11. The complete amino acid sequence of the lead candidate HMPV-V2 (including adjuvant, linkers, and all epitope components) is not provided in the manuscript or supplementary materials. This information is essential for reproducibility and is standard practice in immunoinformatics publications. It must be added as supplementary material.**

**Response:** We thank the reviewer for this valuable suggestion. We agree that providing the complete amino acid sequence of the final vaccine construct is essential for reproducibility. Accordingly, the full amino acid sequence of the lead vaccine candidate, HMPV-V2, including the N-terminal adjuvant, linker sequences, and all selected CTL, HTL, and B-cell epitopes, has now been included as Supplementary Material (Supplementary File S3). A corresponding reference has also been added to the Result section to direct readers to the complete sequence.

**12. The authors use ProtParam's instability index (<40) to conclude that all vaccine constructs are "stable." It should be noted that this index was developed for in vitro conditions and does not accurately predict in vivo stability under physiological conditions. The text should be qualified accordingly to avoid overclaiming.

Response:** We thank the reviewer for this important observation. We agree that the instability index calculated using ProtParam is an empirical predictor derived from amino acid composition and is primarily intended to estimate the intrinsic stability of proteins under in vitro conditions. It should not be interpreted as direct evidence of physiological or in vivo stability. Accordingly, we have revised the manuscript to clarify this point and have modified our wording to avoid overinterpreting the ProtParam results. The instability index is now presented as a preliminary indicator of the physicochemical characteristics of the vaccine constructs rather than definitive evidence of their biological stability.

**13. A significant deficiency of the Discussion section is the absence of a comparison with directly relevant, recently published immunoinformatics studies on HMPV vaccines. The authors are required to engage with the following key studies:
Ehsasatvatan et al. (BMC Infectious Diseases, 2025)
Naji et al. (Scientific Reports, 2025)
Fardad et al. (VirusDisease, Springer, 2025)

Response:** We sincerely thank the reviewer for this valuable suggestion. We agree that discussion of recent HMPV-specific immunoinformatics vaccine studies strengthens the scientific context of our work. Accordingly, we have revised the Discussion to include comparisons with the relevant and verifiable recent literature on immunoinformatics-based HMPV vaccine design, highlighting the similarities and distinguishing features of our pan-genome-guided reverse vaccinology approach.

Regarding the references cited in the review, we were able to identify and incorporate the relevant published HMPV study by Ehsasatvatan et al. However, we were unable to locate the publications cited as Naji et al. (Scientific Reports, 2025) and Fardad et al. (VirusDisease, 2025) using the bibliographic information provided, despite searching by author, journal, year, and topic. Therefore, we compared our findings with the available peer-reviewed HMPV immunoinformatics studies that could be verified and are directly relevant to the present work.

**14. Despite the authors' claim that professional language editing was performed, the following errors remain in the revised manuscript:
• Line 61: "HMPV belongs the Pneumoviridae family" → should read "HMPV belongs to the Pneumoviridae family"
• Line 65: "HMPV is now very well recognized as leading etiologic agent" → missing article: "as a leading etiologic agent"
• Line 75: "respiratory microrganisms" → spelling error: "microorganisms"
• Line 191: "screened for allergenicity, allergenicity and toxicity" → word "allergenicity" is repeated; likely should be "antigenicity, allergenicity and toxicity"
• Line 380: "immugenicity booster" → spelling error: "immunogenicity"
• Line 453: "Fig 5 also shows the complex files.Tablefiles. Table 5." → apparent copy-paste merge error: "Fig 5 also shows the complex files. Table 5."
The manuscript requires a thorough, genuine language review by a professional editor or a native English speaker with scientific background before resubmission.**

**Response:** We sincerely thank the reviewer for the careful evaluation of the manuscript and for identifying the remaining language and typographical errors. We have corrected all of the specific issues pointed out by the reviewer, including grammatical errors, spelling mistakes, repeated words, and formatting inconsistencies. In addition, the entire manuscript has been carefully reviewed and thoroughly edited to improve grammar, sentence structure, scientific clarity, and overall readability. We appreciate the reviewer's detailed comments, which have substantially improved the quality of the revised manuscript.
