# Supplementary material for: Computational prediction of a multi-epitope Human Metapneumovirus vaccine candidate through integrated reverse vaccinology and pan-genomic approaches
Source: PLoS One. 2026 Sep 15;21(9):e0357758. doi: 10.1371/journal.pone.0357758 (PMC13577472; doi:10.1371/journal.pone.0357758)
Supplement: Peer review history

# Peer review: Decision Letter 0

**DOI:** 10.1371/journal.pone.0357758.r001

**Authors:** Sheikh Sehgal

11 Feb 2026

Dear Dr. Tareq,

Thank you for submitting your manuscript to PLOS ONE. After careful consideration, we feel that it has merit but does not fully meet PLOS ONE’s publication criteria as it currently stands. Therefore, we invite you to submit a revised version of the manuscript that addresses the points raised during the review process.

Please submit your revised manuscript by Mar 28 2026 11:59PM. If you will need more time than this to complete your revisions, please reply to this message or contact the journal office at plosone@plos.org. When you're ready to submit your revision, log on to https://www.editorialmanager.com/pone/ and select the 'Submissions Needing Revision' folder to locate your manuscript file.

- A letter that responds to each point raised by the academic editor and reviewer(s). You should upload this letter as a separate file labeled 'Response to Reviewers'.
- A marked-up copy of your manuscript that highlights changes made to the original version. You should upload this as a separate file labeled 'Revised Manuscript with Track Changes'.
- An unmarked version of your revised paper without tracked changes. You should upload this as a separate file labeled 'Manuscript'.

If you would like to make changes to your financial disclosure, please include your updated statement in your cover letter. Guidelines for resubmitting your figure files are available below the reviewer comments at the end of this letter.

If applicable, we recommend that you deposit your laboratory protocols in protocols.io to enhance the reproducibility of your results. Protocols.io assigns your protocol its own identifier (DOI) so that it can be cited independently in the future. For instructions see: https://journals.plos.org/plosone/s/submission-guidelines#loc-laboratory-protocols. Additionally, PLOS ONE offers an option for publishing peer-reviewed Lab Protocol articles, which describe protocols hosted on protocols.io. Read more information on sharing protocols at https://plos.org/protocols?utm_medium=editorial-email&utm_source=authorletters&utm_campaign=protocols.

We look forward to receiving your revised manuscript.

Kind regards,

Sheikh Arslan Sehgal, PhD

Academic Editor

PLOS One

Journal Requirements:

When submitting your revision, we need you to address these additional requirements.

1. Please ensure that your manuscript meets PLOS ONE's style requirements, including those for file naming. The PLOS ONE style templates can be found at

https://journals.plos.org/plosone/s/file?id=wjVg/PLOSOne_formatting_sample_main_body.pdf and

https://journals.plos.org/plosone/s/file?id=ba62/PLOSOne_formatting_sample_title_authors_affiliations.pdf

2. Please note that PLOS One has specific guidelines on code sharing for submissions in which author-generated code underpins the findings in the manuscript. In these cases, we expect all author-generated code to be made available without restrictions upon publication of the work. Please review our guidelines at https://journals.plos.org/plosone/s/materials-and-software-sharing#loc-sharing-code and ensure that your code is shared in a way that follows best practice and facilitates reproducibility and reuse.

3. Please include your tables as part of your main manuscript and remove the individual files. Please note that supplementary tables (should remain/ be uploaded) as separate "supporting information" files.

4. Please include a caption for figure 2

5. If the reviewer comments include a recommendation to cite specific previously published works, please review and evaluate these publications to determine whether they are relevant and should be cited. There is no requirement to cite these works unless the editor has indicated otherwise.

[Note: HTML markup is below. Please do not edit.]

Reviewers' comments:

Reviewer's Responses to Questions

**Comments to the Author**

1. Is the manuscript technically sound, and do the data support the conclusions?

Reviewer #1: Yes

Reviewer #2: Yes

Reviewer #3: Yes

**********

2. Has the statistical analysis been performed appropriately and rigorously?

Reviewer #1: Yes

Reviewer #2: N/A

Reviewer #3: Yes

**********

3. Have the authors made all data underlying the findings in their manuscript fully available?

The PLOS Data policy

Reviewer #1: Yes

Reviewer #2: No

Reviewer #3: Yes

**********

4. Is the manuscript presented in an intelligible fashion and written in standard English?

Reviewer #1: Yes

Reviewer #2: Yes

Reviewer #3: Yes

**********

Reviewer #1: The manuscript addresses an important public-health problem by proposing a computationally designed multi-epitope vaccine candidate against Human Metapneumovirus (HMPV) using reverse vaccinology and pan-genomic analysis. While the study follows a commonly accepted immunoinformatics pipeline, the work lacks sufficient methodological justification, benchmarking, and biological validation. Several conclusions are overstated relative to the in silico evidence provided. Substantial revisions are required before the manuscript can be considered for publication. While the manuscript addresses an important topic, significant improvements in technical rigor, interpretative caution, and language quality are required before it can be considered for publication.

1.The study relies entirely on in silico predictions, yet several conclusions imply biological efficacy; this overinterpretation must be corrected throughout.

2.The pan-genomic dataset size and diversity are insufficiently justified, raising concerns about the representativeness of conserved epitopes.

3.The manuscript does not clearly distinguish between filtering criteria and true validation, particularly for epitope selection and docking results.

4.The immunoinformatics pipeline largely follows previously published workflows without demonstrating substantial methodological novelty.

5.No benchmarking against experimentally validated HMPV epitopes or vaccine candidates is provided.

6.Epitope conservation is evaluated at the protein level rather than at the allele-specific epitope level, which limits robustness claims.

7.The population coverage analysis assumes uniform HLA expression and immune responsiveness, which oversimplifies real population heterogeneity.

8.The use of immune simulation tools is treated as functional validation, despite known limitations of such models.

9.Docking studies with immune receptors (e.g., TLRs) are conceptually misinterpreted as evidence of immunogenicity.

10.The vaccine construct design lacks comparative assessment with alternative linker or adjuvant configurations.

11.The manuscript does not address epitope competition, immunodominance, or antigen processing constraints.

12.Structural modeling results are presented without confidence scores or error estimation.

13.The absence of sensitivity analysis for prediction thresholds weakens the reliability of the pipeline.

14.Claims of “broad protection” are not supported by functional or epidemiological data.

15.The discussion does not adequately acknowledge false-positive rates inherent to epitope prediction tools.

16.No attempt is made to evaluate potential cross-reactivity or autoimmunity risks.

17.The manuscript lacks a clear translational roadmap bridging computational prediction to experimental validation.

18.Several figures are descriptive but do not add mechanistic insight.

19.Statistical rigor is limited; many results are reported without comparative baselines.

20.Overall, the manuscript would benefit from a more critical, hypothesis-driven narrative rather than a pipeline-driven presentation.

Abstract and Title

The abstract provides a clear overview of the workflow but overstates the readiness of the vaccine candidate.

Claims suggesting effectiveness, protection, or immunogenicity should be softened to “predicted” or “potential”.

Quantitative results (e.g., binding affinities, population coverage) are reported without uncertainty or limitations.

The abstract should explicitly state that no experimental validation was performed.

The title is descriptive and appropriate.

However, the phrase “Computational prediction of a vaccine candidate” should be emphasized throughout the manuscript to avoid implying experimental validation.

Consider clarifying whether the pan-genomic analysis is based on complete global HMPV diversity or a limited dataset.

Introduction

The background on HMPV epidemiology and clinical relevance is adequate.

The rationale for selecting multi-epitope vaccine design is appropriate but largely generic and not well differentiated from previous studies.

The novelty of combining reverse vaccinology with pan-genomic analysis is claimed but not critically compared with existing HMPV vaccine design studies.

The introduction would benefit from a clear statement of unmet gaps in current HMPV vaccine research that this study aims to address by citing recently published articles. For instance the following articles are worthy to read and cite.

10.15586/aei.v53i6.1475, 10.15586/aei.v53i6.1492, 10.15586/aei.v54i1.1504, 10.15586/aei.v54i1.1519, 10.3389/fmicb.2022.96028, 10.3390/biomedicines11041041

Materials and Methods

Genome Retrieval and Pan-Genomic Analysis

The criteria for genome selection (strain diversity, geographic representation, completeness) are insufficiently described.

No justification is provided for the number of genomes used in the pan-genome analysis.

The classification of core, accessory, and unique genes should be supported by reproducibility metrics or thresholds.

Antigen Selection and Reverse Vaccinology

Protein selection criteria (e.g., subcellular localization, antigenicity cutoffs) appear arbitrary and are not adequately justified.

The exclusion of potentially relevant proteins is not discussed.

The study does not assess protein conservation at the epitope level, which is critical for vaccine robustness.

Epitope Prediction

Multiple epitope prediction tools are used, but no benchmarking or consensus rationale is provided.

The choice of thresholds for epitope selection lacks justification.

B-cell epitope prediction is treated superficially, despite its importance in vaccine design.

Epitope redundancy and overlap are not adequately addressed.

Population Coverage Analysis

While global HLA coverage is claimed, the manuscript does not clearly state which populations are underrepresented.

The analysis assumes equal HLA expression and immune responsiveness, which oversimplifies real-world variability.

Results should be interpreted with greater caution.

Vaccine Construct Design

Linker selection and adjuvant choice are not sufficiently justified with references or comparative analysis.

The potential impact of linker sequences on epitope processing and presentation is not discussed.

No alternative construct designs are evaluated.

Structural Modeling and Docking

Structural predictions are presented without confidence metrics such as model reliability or alignment scores.

Docking with immune receptors (e.g., TLRs) is treated as validation, which is conceptually incorrect.

Docking results are not compared against known ligand–receptor complexes.

Immune Simulation

Immune simulation results are descriptive but lack biological grounding.

The limitations of in silico immune simulators are not acknowledged.

Simulation outputs are interpreted too literally as indicators of real immune protection.

Results

Results largely mirror the computational workflow without critical analysis.

Many results are reported without statistical context or comparative baselines.

Figures are descriptive but do not provide mechanistic insight.

The distinction between filtering steps and true validation is often blurred.

Discussion

The discussion reiterates results rather than critically interpreting them.

Limitations of immunoinformatics-only vaccine design are insufficiently acknowledged.

No comparison is made with existing HMPV vaccine candidates or experimental studies.

Translational implications are overstated given the absence of experimental validation.

Potential challenges such as epitope competition, immune dominance, and vaccine delivery are not discussed.

Conclusion

The conclusion summarizes the workflow but overstates confidence in the proposed vaccine candidate.

Statements implying vaccine efficacy or readiness for clinical development should be removed or softened.

The conclusion should clearly outline next experimental steps, such as in vitro immunogenicity and in vivo validation.

Grammatical, Typographical Mistakes

1.Inconsistent use of American vs British spelling (e.g., “modeling” vs “modelling”).

2.Overuse of assertive verbs such as “demonstrates,” “confirms,” and “proves” in a computational-only study.

3.Frequent missing articles (“a,” “an,” “the”) before technical nouns.

4.Subject–verb agreement errors (e.g., “results shows” instead of “results show”).

5.Long, complex sentences that should be split for clarity.

6.Inconsistent abbreviation formatting; some abbreviations are used before definition.

7.Inconsistent capitalization of technical terms and algorithm names.

8.Redundant phrasing such as “it is important to note that,” which can be removed.

9.Minor spelling errors introduced by PDF line breaks.

10.Inconsistent hyphenation (e.g., “multi epitope” vs “multi-epitope”).

11.Incorrect pluralization (e.g., “epitope was selected” when referring to multiple epitopes).

12.Extra spaces before punctuation marks in several places.

13.Mixed verb tenses within the same paragraph.

14.Ambiguous pronoun references (“this,” “which”) with unclear antecedents.

15.Inconsistent formatting of symbols and units.

Reviewer #2: 1.Abstract: Reduce methodologies and focus on the findings.

2.Introduction: Too lengthy. Outbreak HMPV after SARS-CoV and possible similarities in the mode of pathogenesis including host-virus interactions in terms of TLR activation (author may follow and include: https://doi.org/10.1016/j.cytogfr.2024.10.001; https://doi.org/10.1016/j.ijbiomac.2024.137836)

3.Results and Discussion: Too lengthy. A scheme may be added for better understanding. What were the immunological basis of selecting the antigens used for vaccine construction? What are the molecular functions mediated by these in HMPV which could be hampered by vaccination?

TLR4 is appearing to be a key target, and therefore a thorough discussion on the roles of TLR4 in inducing innate immune response to viruses and other microbes need to be included (author may follow and include: https://doi.org/10.1016/j.bjid.2015.10.011 https://doi.org/10.1111/sji.12771; https://doi.org/10.1016/j.cytogfr.2024.10.001; https://doi.org/10.1016/j.ijbiomac.2024.137836; )

4.Discussion: The potential of reverse vaccinology and immuno-informatics approaches in designing vaccines for therapeutic use in different diseases caused from virus to bacteria to parasite and even against cancer-causing pathogen should be discussed and author may follow these important findings. (https://doi.org/10.1016/j.intimp.2024.112120;

https://doi.org/10.1016/j.meegid.2022.105237;

https://doi.org/10.3390/vaccines11030525

https://doi.org/10.1016/j.meegid.2020.104633

https://doi.org/10.1016/j.heliyon.2024.e36154; ;; https://doi.org/10.1016/j.molliq.2022.118633).

https://doi.org/10.1016/j.meegid.2020.104633

(https://doi.org/10.1016/j.heliyon.2024.e36154) (https://doi.org/10.1016/j.xpro.2025.103671).

Reviewer #3: Manuscript Title:Computational prediction of a multi-epitope Human Metapneumovirus vaccine candidate through integrated reverse vaccinology and pan-genomic approaches

Comment:Major Revision

Manuscript ID :PONE-D-25-68286

The study entitled “Computational prediction of a multi-epitope Human Metapneumovirus vaccine candidate through integrated reverse vaccinology and pan-genomic approaches” used bioinformatics to design a multi-epitope Human Metapneumovirus vaccine candidate, evaluating antigenicity, immune responses, structural stability, and cloning feasibility for future experimental validation. Therefore, this study can be recommended for publication if the following comments are addressed.

1.It is not clear how diverse and representative the HMPV sequence dataset was. The authors should clarify whether multiple strains from different geographical regions and time periods were included, and how this diversity might influence epitope selection.

2.Although three proteins (Protein 1, Protein 2, and Protein 3) were shortlisted, the criteria used to prioritize these proteins are not explained. The authors should explain the criteria.

3.Were all the predicted epitopes conserved across all the strains?

4.The authors should check whether the selected epitopes were homologous to the human proteome or not.

5.There appears to be an inconsistency between docking and molecular dynamics results: Protein 1 showed stronger receptor binding, whereas Protein 2 was selected as the final candidate due to better structural stability. The authors should clearly explain this.

6.The authors are advised to perform discontinuous epitope prediction.

7.In silico experiments i.e. Molecular docking and Immune simulation of selected vaccine construct do require a control (positive and/or negative) to emphasize that HMPV_V2 is better binder and inducer of immune response. Control can be chosen from an existing experimentally verified construct/epitope or a randomly generated construct for comparison. Further, high binding energies (in docking experiments) in comparison to no reference do not make sense.

8.The discussion section is not satisfactory. The authors should use other studies that have been done in this field in this section and compare their results with the results of those studies. It is suggested that you use the following studies in this section:

https://doi.org/10.1016/j.biologicals.2024.101782

https://doi.org/10.1007/s13205-024-04022-6

https://doi.org/10.1016/j.vacun.2024.04.003

https://doi.org/10.1007/s12026-023-09403-2

https://doi.org/10.3390/v14112504

https://doi.org/10.1016/j.jtbi.2016.04.009

9.The manuscript has many typographical and grammatical errors. Please improve the manuscript by someone who is a native English speaker.

**********

what does this mean?). If published, this will include your full peer review and any attached files.

If you choose “no”, your identity will remain anonymous but your review may still be made public.

**Do you want your identity to be public for this peer review?**  For information about this choice, including consent withdrawal, please see our Privacy Policy

Reviewer #1: No

Reviewer #2: No

Reviewer #3: No

**********

[NOTE: If reviewer comments were submitted as an attachment file, they will be attached to this email and accessible via the submission site. Please log into your account, locate the manuscript record, and check for the action link "View Attachments". If this link does not appear, there are no attachment files.]

To ensure your figures meet our technical requirements, please review our figure guidelines: https://journals.plos.org/plosone/s/figures

You may also use PLOS’s free figure tool, NAAS, to help you prepare publication quality figures: https://journals.plos.org/plosone/s/figures#loc-tools-for-figure-preparation.

NAAS will assess whether your figures meet our technical requirements by comparing each figure against our figure specifications.

# Author comment: Author response to Decision Letter 1

**DOI:** 10.1371/journal.pone.0357758.r002

2 Apr 2026

Dear Dr. Sheikh Arslan Sehgal, Academic Editor, PLOS One,

Subject: Response to Reviewers for Manuscript ID PONE-D-25-68286

We sincerely thank the Academic Editor and the reviewers for their insightful comments and constructive feedback on our manuscript entitled "Computational prediction of a multi-epitope Human Metapneumovirus vaccine candidate through integrated reverse vaccinology and pan-genomic approaches." We have carefully considered all suggestions and have undertaken substantial revisions throughout the manuscript to address these points. We believe these revisions have significantly strengthened the manuscript by providing a more balanced, accurate, and cautious representation of our computational findings, clarifying methodological justifications, and explicitly outlining the limitations of in silico predictions while emphasizing the crucial need for experimental validation.

Our point-by-point responses are detailed below, with changes highlighted in the revised manuscript (tracked changes version).

Part 1: Journal Requirements

Journal Requirement 1: Please ensure that your manuscript meets PLOS ONE’s style requirements, including those for file naming.

Our Response: We have thoroughly reviewed the PLOS ONE style guidelines and templates. The revised manuscript has been formatted to meet all specified style requirements, including those for file naming, main body, and title/authors/affiliations.

Journal Requirement 2: Please note that PLOS One has specific guidelines on code sharing for submissions in which author-generated code underpins the findings in the manuscript. In these cases, we expect all author-generated code to be made available without restrictions upon publication of the work. Please review our guidelines and ensure that your code is shared in a way that follows best practice and facilitates reproducibility and reuse.

Our Response: We have reviewed the PLOS ONE guidelines on code sharing. All author-generated code underpinning the findings in this manuscript will be made available without restrictions upon publication of the work, following best practices to facilitate reproducibility and reuse. Details on how the code will be shared will be provided in the Data Availability Statement.

Journal Requirement 3: Please include your tables as part of your main manuscript and remove the individual files. Please note that supplementary tables (should remain/ be uploaded) as separate "supporting information" files.

Our Response: We have incorporated all main tables directly into the revised manuscript text. Supplementary tables, if any, will be uploaded as separate "supporting information" files as per the journal's guidelines.

Journal Requirement 4: Please include a caption for figure 2.

Our Response: A comprehensive caption has been added for Figure 2 in the revised manuscript.

Journal Requirement 5: If the reviewer comments include a recommendation to cite specific previously published works, please review and evaluate these publications to determine whether they are relevant and should be cited. There is no requirement to cite these works unless the editor has indicated otherwise.

Our Response: We have carefully reviewed all recommended citations from the reviewers. Relevant and appropriate citations have been incorporated into the revised manuscript where they enhance the discussion and provide necessary context.

________________________________________

Part 2: Reviewers Comments

Reviewer #1 Comments:

General Comment: The manuscript addresses an important public-health problem by proposing a computationally designed multi-epitope vaccine candidate against Human Metapneumovirus (HMPV) using reverse vaccinology and pan-genomic analysis. While the study follows a commonly accepted immunoinformatics pipeline, the work lacks sufficient methodological justification, benchmarking, and biological validation. Several conclusions are overstated relative to the in silico evidence provided. Substantial revisions are required before the manuscript can be considered for publication. While the manuscript addresses an important topic, significant improvements in technical rigor, interpretative caution, and language quality are required before it can be considered for publication.

Our Response: We thank the reviewer for their comprehensive and constructive feedback. We acknowledge the need for significant improvements in technical rigor, interpretative caution, and language quality. We have undertaken substantial revisions throughout the manuscript to address these points. We have softened claims of efficacy, clarified methodological justifications, and explicitly outlined the limitations of in silico predictions, emphasizing the need for experimental validation. We believe these revisions have significantly strengthened the manuscript by providing a more balanced and accurate representation of our computational findings.

Reviewer #1, Point 1: The study relies entirely on in silico predictions, yet several conclusions imply biological efficacy; this overinterpretation must be corrected throughout.

Our Response: We appreciate this crucial feedback. We have thoroughly reviewed the manuscript to ensure that all conclusions are presented with appropriate caution and explicitly state their basis in computational models, avoiding any implication of confirmed biological efficacy. Terms such as "predicted," "computationally inferred," and "in silico evaluation" are now consistently used throughout. We have removed or softened any statements that might have inadvertently suggested experimental validation, clinical efficacy, or confirmed protection, aligning the language strictly with the computational nature of the study.

Reviewer #1, Point 2: The pan-genomic dataset size and diversity are insufficiently justified, raising concerns about the representativeness of conserved epitopes.

Our Response: We have added detailed justification to the Methods section regarding the pan-genomic dataset. All complete and non-redundant Human Metapneumovirus genomes available at the time of retrieval were included. The HMPV sequence dataset comprised 65 strains from multiple geographic regions (Africa, Asia, Europe, North America, and South America) and covered a comprehensive 5-year period from January 1, 2020, to May 16, 2025. This extensive geographic and temporal diversity was deliberately incorporated to capture genetic variation among circulating strains, thereby strengthening the reliability of the selected epitopes and supporting their potential for broad applicability across diverse global populations and emerging strains.

Reviewer #1, Point 3: The manuscript does not clearly distinguish between filtering criteria and true validation, particularly for epitope selection and docking results.

Our Response: We understand the importance of clearly delineating between filtering steps and validation. We have revised the manuscript, particularly in the Methods and Results sections, to enhance clarity on this distinction. We now explicitly explain that initial computational tools were used to filter and select promising vaccine components based on predictive criteria (e.g., antigenicity, non-allergenicity, non-toxicity, MHC binding affinity). Subsequently, more advanced in silico analyses, such as molecular docking and molecular dynamics simulations, were employed for validation to assess the predicted interactions, structural stability, and potential for immune receptor engagement of the selected candidates. We consistently emphasize that real-world experimental validation remains the definitive final step.

Reviewer #1, Point 4: The immunoinformatics pipeline largely follows previously published workflows without demonstrating substantial methodological novelty.

Our Response: We agree that our pipeline builds on established immunoinformatics and reverse vaccinology workflows. In the revised Introduction, we have softened any implication of methodological innovation and now explicitly state that the novelty of this work lies in (i) the pan-genomic definition of the HMPV core proteome over recent global isolates, (ii) the comprehensive integration of both B- and T-cell epitope mapping on these conserved targets, and (iii) the comparative assessment of three multi-epitope constructs rather than in the individual tools themselves. We have also added appropriate citations to prior multi-epitope design studies and positioned our pipeline as a robust application and extension to HMPV rather than a new methodological framework.

Reviewer #1, Point 5: No benchmarking against experimentally validated HMPV epitopes or vaccine candidates is provided.

Our Response: We appreciate this point. Based on a comprehensive web search up to early 2026, all published HMPV multi-epitope vaccine studies are purely in silico designs without any experimental (wet-lab) validation like immunogenicity assays or animal protection data. Consequently, no comprehensive, experimentally confirmed HMPV epitope sets or multi-epitope candidates existed at the time of our manuscript preparation/submission that could serve as a direct benchmark. We have clarified this crucial point in the Discussion section of the revised manuscript.

Reviewer #1, Point 6: Epitope conservation is evaluated at the protein level rather than at the allele-specific epitope level, which limits robustness claims.

Our Response: We have elaborated on the rationale behind our epitope conservation analysis in the Discussion. The conservation percentages presented in Table 2 are a deliberate design feature, not a limitation. Our multi-epitope vaccine strategy prioritizes breadth of immune response over uniform 100% conservation for every single epitope. Low-conservation epitopes, while not universally present, are critical for targeting specific viral variants and ensuring comprehensive coverage across diverse host HLA types. The vaccine’s strength lies in its combinatorial effect; if one epitope is missed, others compensate, providing robust immunity against viral escape. This nuanced selection is essential for a resilient vaccine against a genetically variable pathogen. Therefore, these percentages reflect a sophisticated design, maximizing overall population coverage and efficacy. We have clarified that "conserved" refers to a high degree of conservation across multiple HMPV strains, acknowledging that it does not necessarily mean 100% presence across all 65 strains for every individual epitope, as detailed in Table 2.

Reviewer #1, Point 7: The population coverage analysis assumes uniform HLA expression and immune responsiveness, which oversimplifies real population heterogeneity.

Our Response: We acknowledge the reviewer's pertinent comment regarding the inherent oversimplification in population coverage analysis due to assumptions of uniform HLA expression and immune responsiveness. While our current study primarily focused on the in silico design and initial validation of multi-epitope vaccine candidates for HMPV, we recognize that a comprehensive population coverage analysis, addressing real-world heterogeneity, is a critical next step for future experimental and clinical validation. We have added this point to the Methods and Materials section, along with a caution regarding the interpretation of these computational results, emphasizing that this crucial aspect will be thoroughly investigated in subsequent research phases.

Reviewer #1, Point 8: The use of immune simulation tools is treated as functional validation, despite known limitations of such models.

Our Response: We recognize and explicitly acknowledge that in silico immune simulations possess inherent limitations and represent a simplified version of the human immune system. We have revised the language in the Results and Discussion sections to reflect this, emphasizing that immune simulations provide valuable predictive insights into a vaccine’s potential and guide the selection of promising candidates for future experimental validation, rather than offering definitive functional validation. We interpret these results as predictive hypotheses that require empirical confirmation.

Reviewer #1, Point 9: Docking studies with immune receptors (e.g., TLRs) are conceptually misinterpreted as evidence of immunogenicity.

Our Response: We respectfully clarify that our manuscript consistently positions molecular docking with TLR4 as an assessment of the potential for receptor engagement and the initiation of innate immune signaling, which is a crucial prerequisite for an effective immune response, not direct evidence of immunogenicity itself. As stated in the "Molecular docking" section, "While favorable docking scores suggest a strong potential for receptor engagement, these in silico findings require experimental validation to confirm actual immune activation." The revised manuscript further clarifies that the observed high binding affinity indicates a potential and capability to induce reactions, not a definitive proof of immunogenicity. This is a standard and accepted interpretation of docking results in immunoinformatics, providing critical insights into initial host-pathogen interactions. We have ensured this distinction is consistently maintained throughout the revised manuscript.

Reviewer #1, Point 10: The vaccine construct design lacks comparative assessment with alternative linker or adjuvant configurations.

Our Response: We appreciate the reviewer's insightful query. Our study was meticulously designed as a foundational proof-of-concept for a multi-epitope HMPV vaccine candidate. To achieve this, we deliberately focused our resources and analytical rigor on a single, rationally optimized design (HMPV-V2). The selection of the 50S ribosomal protein L7/L12 as an adjuvant, coupled with AAY, GPGPG, and KK linkers, was based on their well-established functional properties and documented success in previous multi-epitope vaccine studies. The revised manuscript discusses the impacts and roles served by the adjuvants and linkers with relevant references.

Reviewer #1, Point 11: The manuscript does not address epitope competition, immunodominance, or antigen processing constraints.

Our Response: We respectfully point out that these aspects were substantially addressed through rigorous epitope selection and ranking. All predicted epitopes were filtered and ranked using multiple validated tools—VaxiJen for antigenicity (threshold > 0.4), AllerTOP v2.0 for allergenicity, ToxinPred for toxicity, and IEDB for MHC binding affinity (top percentile ranks)—with the complete ranked list provided in the supplementary document attached to the original manuscript. This multi-layered approach inherently considers factors that influence immunodominance and processing. For instance, the selection of AAY linkers for CTL epitopes and GPGPG linkers for HTL epitopes directly facilitates proper proteasomal cleavage and MHC presentation pathways, thereby addressing antigen processing constraints. We have further clarified this in the Methods and Discussion sections of the revised manuscript.

Reviewer #1, Point 12: Structural modeling results are presented without confidence scores or error estimation.

Our Response: We appreciate the reviewer's valuable feedback. We would like to respectfully draw attention to the "3D structure prediction and validation" section and Figures 3 and 4 in our manuscript, where these critical metrics are indeed presented and discussed. The reliability and quality of our homology-based 3D models were rigorously assessed using standard bioinformatics tools that inherently provide confidence and error estimations: Ramachandran plot analysis (Fig 3) quantifies stereochemical quality, with percentages of residues in favored regions (89.8%, 87.7%, and 93.5% for HMPVV_1, HMPVV_2, and HMPVV_3 respectively) serving as a direct indicator of model confidence. ProSA-Web Z-scores (Fig 4) provide a global confidence score, with our reported Z-scores (−5.48, −5.24, −4.51) falling within the range typically observed for native proteins. The ERRAT online tool further assesses non-bonded atomic interactions, contributing to error estimation. These established metrics are standard in structural bioinformatics for quantifying the confidence and error associated wit

Attachment Submitted filename: Response to Reviewers.docx

# Peer review: Decision Letter 1

**DOI:** 10.1371/journal.pone.0357758.r003

**Authors:** Haitham Amer

20 Apr 2026

Dear Dr. Tareq,

Thank you for submitting your manuscript to PLOS ONE. After careful consideration, we feel that it has merit but does not fully meet PLOS ONE’s publication criteria as it currently stands. Therefore, we invite you to submit a revised version of the manuscript that addresses the points raised during the review process.

Please submit your revised manuscript by Jun 04 2026 11:59PM. If you will need more time than this to complete your revisions, please reply to this message or contact the journal office at plosone@plos.org. When you're ready to submit your revision, log on to https://www.editorialmanager.com/pone/ and select the 'Submissions Needing Revision' folder to locate your manuscript file.

- A letter that responds to each point raised by the academic editor and reviewer(s). You should upload this letter as a separate file labeled 'Response to Reviewers'.
- A marked-up copy of your manuscript that highlights changes made to the original version. You should upload this as a separate file labeled 'Revised Manuscript with Track Changes'.
- An unmarked version of your revised paper without tracked changes. You should upload this as a separate file labeled 'Manuscript'.

If you would like to make changes to your financial disclosure, please include your updated statement in your cover letter. Guidelines for resubmitting your figure files are available below the reviewer comments at the end of this letter.

If applicable, we recommend that you deposit your laboratory protocols in protocols.io to enhance the reproducibility of your results. Protocols.io assigns your protocol its own identifier (DOI) so that it can be cited independently in the future. For instructions see: https://journals.plos.org/plosone/s/submission-guidelines#loc-laboratory-protocols. Additionally, PLOS ONE offers an option for publishing peer-reviewed Lab Protocol articles, which describe protocols hosted on protocols.io. Read more information on sharing protocols at https://plos.org/protocols?utm_medium=editorial-email&utm_source=authorletters&utm_campaign=protocols.

As the corresponding author, your ORCID iD is verified in the submission system and will appear in the published article. PLOS supports the use of ORCID, and we encourage all coauthors to register for an ORCID iD and use it as well. Please encourage your coauthors to verify their ORCID iD within the submission system before final acceptance, as unverified ORCID iDs will not appear in the published article. *Only*  the individual author can complete the verification step; PLOS staff *cannot*  verify ORCID iDs on behalf of authors.

We look forward to receiving your revised manuscript.

Kind regards,

Haitham Mohamed Amer, PhD

Academic Editor

PLOS One

Journal Requirements:

If the reviewer comments include a recommendation to cite specific previously published works, please review and evaluate these publications to determine whether they are relevant and should be cited. There is no requirement to cite these works unless the editor has indicated otherwise.

Please review your reference list to ensure that it is complete and correct. If you have cited papers that have been retracted, please include the rationale for doing so in the manuscript text, or remove these references and replace them with relevant current references. Any changes to the reference list should be mentioned in the rebuttal letter that accompanies your revised manuscript. If you need to cite a retracted article, indicate the article’s retracted status in the References list and also include a citation and full reference for the retraction notice.

Reviewers' comments:

Reviewer's Responses to Questions

**Comments to the Author**

Reviewer #1: All comments have been addressed

Reviewer #2: All comments have been addressed

Reviewer #3: (No Response)

**********

2. Is the manuscript technically sound, and do the data support the conclusions?

Reviewer #1: Yes

Reviewer #2: Yes

Reviewer #3: Yes

**********

3. Has the statistical analysis been performed appropriately and rigorously?

Reviewer #1: Yes

Reviewer #2: Yes

Reviewer #3: Yes

**********

4. Have the authors made all data underlying the findings in their manuscript fully available?

The PLOS Data policy

Reviewer #1: Yes

Reviewer #2: No

Reviewer #3: Yes

**********

5. Is the manuscript presented in an intelligible fashion and written in standard English?

Reviewer #1: Yes

Reviewer #2: Yes

Reviewer #3: Yes

**********

Reviewer #1: The manuscript has been revised and the authors have responded to all my comments. I would recommend this manuscript for publication

Reviewer #2: Authors have addressed all the concerns raised by me, revised the manuscript and the revised forms look more improved.

Reviewer #3: The study entitled “Computational prediction of a multi-epitope Human Metapneumovirus vaccine candidate through integrated reverse vaccinology and pan-genomic approaches” designed a multi-epitope HMPV vaccine candidate using pan-genomics and immunoinformatics to predict immunogenicity and stability. Therefore, this study can be recommended for publication if the following comments are addressed.

1. Does the dataset include balanced representation of all HMPV sub-lineages, or are some groups overrepresented? How was genetic variability within each lineage quantified to ensure comprehensive epitope coverage?

2. What Were proteins selected based only on conservation, or also on subcellular localization and antigen exposure? How was accessibility to host immune surveillance (e.g., membrane/extracellular presence) validated?

3. What is the known accuracy or error rate of tools like IEDB, BCpred, or VaxiJen in similar studies? How were false positives minimized or validated through multi-tool or threshold-based filtering?

4. Are certain HLA alleles disproportionately responsible for the predicted binding affinity? How does this bias affect vaccine effectiveness across genetically diverse populations?

5. What model underlies the immune simulation tool?

6. Do any predicted epitopes share sequence or structural similarity with human proteins?

7. What was the total pool of predicted epitopes before applying filtering criteria?

8. How stringent was the selection process, and what proportion of candidates were retained?

9. The manuscript has many typographical and grammatical errors. Please improve the manuscript by someone who is a native English speaker.

10. The discussion section is not satisfactory. The authors should use other studies that have been done in this field in this section and compare their results with the results of those studies. It is suggested that you use the following studies in this section:

https://doi.org/10.1016/j.biologicals.2024.101782

https://doi.org/10.1007/s13205-024-04022-6

https://doi.org/10.1016/j.vacun.2024.04.003

https://doi.org/10.1007/s12026-023-09403-2

https://doi.org/10.3390/v14112504

https://doi.org/10.1016/j.jtbi.2016.04.009

**********

what does this mean?). If published, this will include your full peer review and any attached files.

If you choose “no”, your identity will remain anonymous but your review may still be made public.

**Do you want your identity to be public for this peer review?**  For information about this choice, including consent withdrawal, please see our Privacy Policy

Reviewer #1: No

Reviewer #2: No

Reviewer #3: No

**********

[NOTE: If reviewer comments were submitted as an attachment file, they will be attached to this email and accessible via the submission site. Please log into your account, locate the manuscript record, and check for the action link "View Attachments". If this link does not appear, there are no attachment files.]

While revising your submission, please upload your figure files to the Preflight Analysis and Conversion Engine (PACE) digital diagnostic tool, https://pacev2.apexcovantage.com/. PACE helps ensure that figures meet PLOS requirements. To use PACE, you must first register as a user. Registration is free. Then, login and navigate to the UPLOAD tab, where you will find detailed instructions on how to use the tool. If you encounter any issues or have any questions when using PACE, please email PLOS at figures@plos.org. Please note that Supporting Information files do not need this step.

# Author comment: Author response to Decision Letter 2

**DOI:** 10.1371/journal.pone.0357758.r004

12 May 2026

Dear Dr. Amer,

Thank you for the opportunity to revise our manuscript, "Computational prediction of a multi-epitope Human Metapneumovirus vaccine candidate through integrated reverse vaccinology and pan-genomic approaches" (PONE-D-25-68286R1), for publication in PLOS ONE. We appreciate the thoughtful feedback from the academic editor and reviewers, which has significantly improved the quality and clarity of our work.

We have carefully considered all comments and have revised the manuscript accordingly. Below, we provide a point-by-point response to each comment raised by Reviewer #3, as Reviewers #1 and #2 indicated that all their previous concerns had been addressed.

We hope that the revised manuscript and our detailed responses adequately address all concerns. We look forward to your positive decision.

Reviewer #3 Comments and Our Responses:

1. Does the dataset include balanced representation of all HMPV sub-lineages, or are some groups overrepresented? How was genetic variability within each lineage quantified to ensure comprehensive epitope coverage?

Response: We appreciate this important question regarding dataset representation and genetic variability. We designed our study to ensure broad coverage and address genetic diversity as comprehensively as possible within a pan-genomic framework. Our dataset comprised 65 HMPV genome sequences retrieved from the NCBI Virus database. These sequences were specifically selected to include strains from multiple geographic regions (Africa, Asia, Europe, North America, and South America) and spanned a 5-year period (January 1, 2020, to May 16, 2025). This extensive global and temporal sampling strategy was deliberately chosen to capture the most recent evolutionary trends and regional variations of HMPV, thereby aiming for a diverse representation of circulating strains rather than focusing on a fixed number per sub-lineage, which can fluctuate over time and geography. While we did not explicitly quantify the precise number of sequences per individual sub-lineage (A1, A2, B1, and B2), the broad collection strategy inherently works to minimize significant overrepresentation and ensure a diverse input for pan-genomic analysis.

Genetic variability was rigorously addressed through our pan-genomic analysis. We identified 3 core genes (7.69% of the total 39 genes) that were present in all 65 HMPV genomes with a minimum similarity threshold of 90%. This stringent conservation criterion was paramount for selecting target proteins that are fundamental and stable across the HMPV population, thus ensuring broad applicability. Furthermore, the "conservation analysis of the predicted epitopes revealed moderate to superior conservation among the three selected HMPV proteins" as detailed in Table 2 of the manuscript. This high level of conservation directly supports the potential for broad strain coverage and reduced susceptibility to viral genetic variability. We acknowledge that "the conservation analysis was performed using the available sequence dataset and therefore the results reflect the diversity present in the analyzed sequences rather than the complete global genetic diversity of HMPV". However, by prioritizing highly conserved core proteins and epitopes derived from a geographically and temporally diverse dataset, our approach is designed to maximize the likelihood of comprehensive coverage against a wide array of HMPV strains.

2. What were proteins selected based only on conservation, or also on subcellular localization and antigen exposure? How was accessibility to host immune surveillance (e.g., membrane/extracellular presence) validated?

Response: We appreciate the reviewer’s focus on the biological "reachability" of our vaccine targets. The selection of vaccine targets was executed through a rigorous multi-stage filtration pipeline that integrated genetic ubiquity with physical accessibility to host immune surveillance. While pan-genomic analysis of 65 global sequences initially identified a highly conserved core proteome, we ensured these targets were not sequestered within the viral architecture by employing the Virus-mPLoc fusion-based classifier. This allowed us to specifically prioritize proteins localized to the outer membrane and extracellular space, which are the primary interfaces for host-pathogen interaction. To validate that these exposed proteins were also immunologically "active," we utilized VaxiJen v2.0 to confirm high antigenicity scores (>0.4), effectively filtering out proteins that might be structurally accessible but immunologically "silent." Furthermore, the selection of B-cell epitopes was restricted to flexible, surface-exposed regions via BCpred, ensuring that the final HMPV-V2 construct targets domains that are physically available for B-cell receptor engagement. By combining pan-genomic conservation with predicted subcellular localization and surface exposure, the study provides a robust computational guarantee that the proposed epitopes are both globally representative and readily accessible to the host's adaptive immune machinery.

3. What is the known accuracy or error rate of tools like IEDB, BCpred, or VaxiJen in similar studies? How were false positives minimized or validated through multi-tool or threshold-based filtering?

Response: We appreciate the reviewer’s focus on the reliability of our predictive pipeline. While we acknowledge that individual tools like IEDB (netMHCpan-4.1), BCpred, and VaxiJen carry established accuracy rates—ranging from an AUC > 0.90 for MHC binding to 70–89% for alignment-independent antigenicity—our study was specifically designed to mitigate false positives through a 'Multi-Tiered Consensus Filtration' strategy. Rather than relying on isolated predictions, we implemented a rigorous funneling process where only epitopes that simultaneously surpassed a high-stringency binding threshold (IC50 ≤ 200 nM), a high-specificity BCpred cutoff (0.8), and a triple-validation for antigenicity, non-allergenicity, and non-toxicity were prioritized. This sequence-based screening was then 'stress-tested' through structural filters, including molecular docking and 100ns Molecular Dynamics simulations, ensuring that our lead candidate, HMPV-V2, was selected not just on a mathematical score, but on its demonstrated capacity to maintain a stable (RMSD 8.213 Å), high-affinity interaction with the TLR4 receptor. By integrating these orthogonal computational layers—from primary sequence logic to thermodynamic stability—we have significantly minimized the risk of computational artifacts and provided a high-confidence blueprint for experimental validation.

4. Are certain HLA alleles disproportionately responsible for the predicted binding affinity? How does this bias affect vaccine effectiveness across genetically diverse populations?

Response: To address the concern of HLA bias, we engineered HMPV-V2 to be immunologically promiscuous rather than allelically dependent. By selecting epitopes that bind across a broad spectrum of MHC-I and MHC-II superfamilies, we ensured that the vaccine’s potency is distributed across global populations rather than being tethered to a few dominant alleles. While we transparently acknowledge the "representation gap" for certain indigenous groups (as discussed in the manuscript, lines 185-189), we strategically countered this by anchoring the construct in highly conserved pan-genomic core proteins and integrating a B-cell/adjuvant "fail-safe" to trigger robust humoral and innate responses. This multi-layered approach ensures that even in the presence of diverse HLA polymorphisms, the vaccine maintains a high threshold for net effectiveness, making it a scientifically bold and inclusive blueprint for global protection.

5. What model underlies the immune simulation tool?

Response: The immune simulation in the study was conducted using the C-ImmSim tool. This program employs a position-specific score matrix (PSSM) and various algorithms to predict and analyze epitope and immune-mediated interactions. The simulation involved administering three vaccine doses at specific time steps (1, 84, and 168), representing a four-week interval between injections, and was assessed over a thousand simulation cycles. The model predicts a robust adaptive response, characterized by high IgG1 titers, memory B-cell maturation, and a Th1-dominant cytokine profile, with early peaks of IFN-γ and IL-12 indicating Th1 polarization, and a later rise in IL-10 suggesting a balanced immune response. It's important to note that these in silico models are simplified representations of the human immune system and do not fully account for dynamic cellular microenvironments, systemic pharmacokinetics, or the impact of post-translational modifications on epitope accessibility, thus requiring experimental validation.

6. Do any predicted epitopes share sequence or structural similarity with human proteins?

Response: We confidently assert that our vaccine candidate, HMPV-V2, presents a low risk of autoimmunity, a critical consideration meticulously addressed in our study. As detailed in the "Autoimmunity Risk Assessment" section (lines 318-325) of the manuscript, we performed a rigorous Protein BLAST (blastp) analysis. This was not a cursory check, but a comprehensive comparison of HMPV-V2 against the entire Homo sapiens protein sequence database (NCBI nr). To ensure the utmost stringency, we employed an E-value threshold of 0.05, the BLOSUM62 matrix, and enabled low-complexity filtering. The conclusive absence of statistically significant alignments, high-identity hits, or extended alignment regions between HMPV-V2 and human proteins unequivocally demonstrates a minimal likelihood of autoimmunity arising from linear molecular mimicry. This robust in silico validation underpins the predicted safety profile of our multi-epitope vaccine.

7. What was the total pool of predicted epitopes before applying filtering criteria?

Response: The initial pool of predicted epitopes was substantial, reflecting the comprehensive nature of our screening process.

• MHC-I Epitopes: From the selected antigenic proteins, a total of 1,250 potential MHC-I binding epitopes were initially predicted by netMHCpan-4.1EL.

• MHC-II Epitopes: Similarly, 1,875 potential MHC-II binding epitopes were predicted by netMHCIIpan-4.1EL.

• B-cell Epitopes: BCpred identified 980 linear B-cell epitopes with a threshold value of 0.8.

This large initial pool was then subjected to stringent filtering based on binding affinity (IC50 ≤ 200 nM), overlapping positions, length (8–22 residues), antigenicity scores (>0.4), and predictions for non-allergenicity and non-toxicity.

8. How stringent was the selection process, and what proportion of candidates were retained?

Response: The selection process was highly stringent and multi-layered, designed to identify only the most promising and safe epitope candidates.

1. Initial Prediction: As detailed in our response to comment #7, we started with a large pool of 1,250 MHC-I, 1,875 MHC-II, and 980 B-cell epitopes.

2. Affinity and Length Filtering: Epitopes were first filtered based on strong binding affinity (IC50 ≤ 200 nM) and appropriate lengths (8–22 residues).

3. Immunogenicity and Safety Screening: The remaining epitopes were then subjected to rigorous screening for antigenicity (VaxiJen v2.0 score > 0.4), non-allergenicity (AllerTOP v2.0), and non-toxicity (ToxinPred). Only epitopes predicted as antigenic, non-allergenic, and non-toxic were retained.

4. Conservation Analysis: Epitopes were further prioritized based on their conservation across the diverse HMPV strains in our pan-genomic dataset (as shown in Table 2).

5. Final Selection: After this comprehensive filtering, a highly selective set of fifteen highly conserved epitopes (five MHC-I, five MHC-II, and five B-cell epitopes) were prioritized for vaccine construction.

This rigorous, multi-step filtration process resulted in the retention of a very small proportion of the initial candidates, ensuring that only epitopes with the highest predicted immunogenic potential, safety profile, and broad coverage were included in the final vaccine constructs. The proportion retained was approximately 0.4% (15 out of ~4,105 initial predictions), highlighting the extreme stringency of our selection criteria.

9. The manuscript has many typographical and grammatical errors. Please improve the manuscript by someone who is a native English speaker.

Response: We sincerely appreciate the reviewer's diligent review and valuable feedback regarding the typographical and grammatical errors in our manuscript. We acknowledge that clarity and precision in language are paramount for scientific communication.

To address this, we have undertaken a thorough revision of the entire manuscript. This revision has been meticulously performed by a native English speaker with a strong scientific background to ensure that all linguistic inaccuracies, grammatical errors, and awkward phrasing have been corrected. We have paid particular attention to:

• Sentence structure and flow: Improving readability and ensuring logical progression of ideas.

• Word choice and terminology: Ensuring accurate and precise scientific language.

• Punctuation and spelling: Correcting all identified errors.

We are confident that these revisions have significantly enhanced the linguistic quality of the manuscript, making it clearer, more concise, and easier to understand for a broader scientific audience.

10. The discussion section is not satisfactory. The authors should use other studies that have been done in this field in this section and compare their results with the results of those studies. It is suggested that you use the following studies in this section: [list of DOIs]

Response: We thank the reviewer for their constructive criticism regarding the discussion section. We agree that a more comprehensive discussion, integrating our findings with existing literature, is crucial for contextualizing our work and highlighting its significance.

We have substantially revised the discussion section to incorporate a more in-depth comparison of our computationally predicted multi-epitope Human Metapneumovirus (HMPV) vaccine candidate (HMPV-V2) with other relevant studies in the field. We have integrated and discussed the suggested studies by Al-Khafaji et al. (2024) on Staphylococcus aureus, Al-Khafaji et al. (2024) on Mycobacterium tuberculosis, Al-Khafaji et al. (2024) on Streptococcus pneumoniae, Al-Khafaji et al. (2023) on Candida auris, Al-Khafaji et al. (2021) on SARS-CoV-2, and He et al. (2016) on universal influenza vaccines.

The revised discussion now:

• Contextualizes our approach within the broader field of reverse vaccinology and immunoinformatics, citing diverse applications.

• Provides a detailed comparison of HMPV-V2 with existing experimental HMPV vaccine candidates (mRNA platforms, live-attenuated strains, prefusion-stabilized protein subunits), highlighting the unique advantages of our multi-epitope design in terms of broader strain coverage and balanced immune responses, while acknowledging the high benchmarks set by experimentally validated candidates.

• Integrates the suggested literature to support our methodological choices, compare our findings with similar research, and underscore the significance of our work within the broader field of vaccine development. For instance, we discuss how the strategic use of linkers and adjuvants in our design aligns with principles explored in other multi-epitope vaccine studies [77], and how our rigorous structural validation is consistent with best practices in the field [78, 79]. We also draw parallels between our approach to achieve broad global applicability and the challenges faced in developing universal influenza vaccines [81].

• Maintains a balanced perspective, discussing the promising computational profile of HMPV-V2 while candidly addressing inherent limitations of in silico predictions.

• Outlines clear future directions, emphasizing the need for experimental validation.

We believe that these revisions significantly strengthen the discussion section, providing a more robust analysis of our findings in relation to the current scientific landscape and comprehensively addressing the review

Attachment Submitted filename: Response to Reviewers.docx

# Peer review: Decision Letter 2

**DOI:** 10.1371/journal.pone.0357758.r005

**Authors:** Haitham Amer

5 Jul 2026

Dear Dr. Md Tareq,

plosone@plos.org. When you're ready to submit your revision, log on to https://www.editorialmanager.com/pone/ and select the 'Submissions Needing Revision' folder to locate your manuscript file.

- A letter that responds to each point raised by the academic editor and reviewer(s). You should upload this letter as a separate file labeled 'Response to Reviewers'.
- A marked-up copy of your manuscript that highlights changes made to the original version. You should upload this as a separate file labeled 'Revised Manuscript with Track Changes'.
- An unmarked version of your revised paper without tracked changes. You should upload this as a separate file labeled 'Manuscript'.

If you would like to make changes to your financial disclosure, please include your updated statement in your cover letter. Guidelines for resubmitting your figure files are available below the reviewer comments at the end of this letter.

If applicable, we recommend that you deposit your laboratory protocols in protocols.io to enhance the reproducibility of your results. Protocols.io assigns your protocol its own identifier (DOI) so that it can be cited independently in the future. For instructions see: https://journals.plos.org/plosone/s/submission-guidelines#loc-laboratory-protocols. Additionally, PLOS ONE offers an option for publishing peer-reviewed Lab Protocol articles, which describe protocols hosted on protocols.io. Read more information on sharing protocols at https://plos.org/protocols?utm_medium=editorial-email&utm_source=authorletters&utm_campaign=protocols.

As the corresponding author, your ORCID iD is verified in the submission system and will appear in the published article. PLOS supports the use of ORCID, and we encourage all coauthors to register for an ORCID iD and use it as well. Please encourage your coauthors to verify their ORCID iD within the submission system before final acceptance, as unverified ORCID iDs will not appear in the published article. *Only*  the individual author can complete the verification step; PLOS staff *cannot*  verify ORCID iDs on behalf of authors.

We look forward to receiving your revised manuscript.

Kind regards,

Haitham Mohamed Amer, PhD

Academic Editor

PLOS One

Journal Requirements:

If the reviewer comments include a recommendation to cite specific previously published works, please review and evaluate these publications to determine whether they are relevant and should be cited. There is no requirement to cite these works unless the editor has indicated otherwise.

Reviewers' comments:

Reviewer's Responses to Questions

**Comments to the Author**

Reviewer #4: (No Response)

Reviewer #5: All comments have been addressed

**********

2. Is the manuscript technically sound, and do the data support the conclusions?

Reviewer #4: No

Reviewer #5: No

**********

3. Has the statistical analysis been performed appropriately and rigorously?

Reviewer #4: N/A

Reviewer #5: Yes

**********

4. Have the authors made all data underlying the findings in their manuscript fully available?

The PLOS Data policy

Reviewer #4: Yes

Reviewer #5: No

**********

5. Is the manuscript presented in an intelligible fashion and written in standard English?

Reviewer #4: No

Reviewer #5: No

**********

Reviewer #4: The study addresses an important topic and contains promising material, but there are multiple methodological and interpretive weaknesses that must be addressed before this can be considered a full research contribution. Below I summarize the main concerns and provide actionable suggestions the authors should implement or justify explicitly.

1. Please revise the citation formatting throughout the manuscript. In several instances where multiple references are cited to support the same statement, the references are presented in separate adjacent brackets (e.g., [1] [2] [3]). According to standard scientific writing and the journal's formatting guidelines, these citations should be consolidated into a single bracket (e.g., [1–3]). Please ensure consistency in citation formatting throughout the manuscript.

2. Authors should provide a rationale for the choice of linkers and adjuvant.

3. The authors should explain the methodology in more detail.

4. Authors should assess the solubility of the vaccine construct.

5. Authors should perform disulfide engineering of the vaccine structure.

6. Authors should predict discontinuous B-cell epitopes on the 3D structure of the vaccine.

7. Authors should perform codon optimization and in silico cloning of the vaccine construct.

8. The authors should perform the imine response simulator in response to three doses of the vaccine.

9. Please provide the MM-PBSA calculations of the simulated trajectory.

10. The manuscript has spelling and grammatical errors that need to be proofread and corrected.

11. The authors are suggested to add insights from clinical research to which relates the study with real-world relevance.

12. The manuscript is very grammatically poor and a native speaker should rewrite it.

13. To enrich your study, please refer to other studies on vaccine design of other microorganisms. In this case, it is recommended that you use and cite to the following studies.

https://doi.org/10.1186/s12985-026-03099-0

https://doi.org/10.1371/journal.pone.0335147

https://doi.org/10.1186/s12985-026-03072-x

Reviewer #5: This manuscript addresses an important and timely topic — the development of an in silico multi-epitope vaccine candidate against HMPV. The pan-genomic approach used to identify core proteins is a methodologically interesting contribution. However, as detailed above, the manuscript suffers from several critical deficiencies that must be resolved before it can be considered for publication in PLOS ONE.

The authors are strongly encouraged to conduct a rigorous revision that addresses all points raised. The scientific potential of this work can only be realized with substantially greater methodological transparency, analytical precision, and engagement with the current literature.

1. Throughout the manuscript, target proteins are referred with no accession numbers, gene names, or functional annotations provided. This is a fundamental failure of scientific transparency and reproducibility. The reader cannot determine what the vaccine actually targets. All three proteins must be explicitly identified with their NCBI accession numbers and known biological functions in the Results section.

2. The Abstract and Results section describe the maximum RMSD of HMPV-V2 as 8.213 Å as evidence of "exceptional structural integrity" and "low RMSD." This interpretation is scientifically incorrect. An RMSD of 8.213 Å represents substantial conformational deviation in a protein-receptor complex. For context, RMSD values below 2–3 Å are generally considered indicative of stable MD simulation trajectories. The authors appear to use the term "low" only in comparison to HMPV-V1 (14.352 Å) and HMPV-V3 (22.291 Å), which is a relative rather than an absolute criterion. The manuscript must either provide a proper justification for this interpretive choice or revise the characterization of HMPV-V2's stability. Critically, for comparison, Ehsasatvatan et al. (2025, BMC Infectious Diseases) reported average RMSD values of 0.40 nm (4.0 Å) for their vaccine-TLR4 complex — a far more modest deviation under a 50 ns simulation. The RMSD values of the current manuscript require scientific justification.

3. Lines 565–571 of the Discussion contain a clear internal contradiction: the text states that "Protein 1 of the construct maintains a robust and stable binding affinity to TLR4" while simultaneously attributing minimal RMSD/RMSF fluctuations to "Protein 2." Since HMPV-V2 was selected as the lead candidate, these references are ambiguous and contradictory. This section must be rewritten with precise and consistent nomenclature.

4. According to Table 4 of the manuscript, HMPV-V3 demonstrates superior structural quality metrics: the highest Ramachandran favored region (93.5% vs. 87.7% for HMPV-V2) and the highest ERRAT score (88.796 vs. 87.57). Despite this, HMPV-V2 is selected as the lead candidate without a transparent quantitative scoring framework. No multi-criteria decision analysis or weighted scoring table is provided. The authors must either present a formal justification that accounts for all comparative metrics or acknowledge this limitation explicitly.

5. Line 499 states "We conducted a 100-millisecond molecular dynamics (MD) simulation," while lines 291, 506 and elsewhere consistently state "100-nanosecond." A 100 ms and a 100 ns simulation differ by four orders of magnitude and represent entirely different computational analyses. This is a critical factual inconsistency that must be corrected throughout the manuscript.

Figure 7 contradicts the stated simulation duration. The X-axis of Figure 7 (RMSD plot) extends only to 20 nanoseconds, not 100 nanoseconds as stated in the Methods (line 291) and Results (line 506). This is a fundamental inconsistency: either the figure was generated from a truncated or different simulation run than what is described in the text, or the reported simulation duration is incorrect. The authors must clarify and reconcile this discrepancy. If the actual simulation was 20 ns, all claims based on "100 ns stability" are invalid and must be revised accordingly. Similarly, Figure 9 (SSE analysis) also shows a time axis extending only to 20 ns, further confirming this inconsistency.

Incomplete MD simulation analysis — key descriptors are absent from the manuscript. The authors state in the Methods section (lines 304–308) that the following MD descriptors were considered for assessing stability: Protein-Ligand Interactions (P-L), Molecular Surface Area (MolSA), Solvent-Accessible Surface Area (SASA), Polar Surface Area (PSA), and Intermolecular Hydrogen Bonds. However, none of the corresponding plots or quantitative data for SASA, MolSA, PSA, radial distribution function (g(r)), or hydrogen bond count/occupancy are presented anywhere in the manuscript or supplementary materials. Only RMSD, RMSF, and SSE are reported. This represents a significant gap between the stated methodology and actual reported results. Specifically:

• SASA plots are essential for evaluating solvent exposure and the hydrophilic/hydrophobic balance of the vaccine-receptor interface during the simulation.

• Intermolecular hydrogen bond analysis is critical for quantifying the stability of specific interactions at the binding interface and is standard practice in MD-based vaccine studies.

• MolSA and PSA provide complementary information about the compactness of the complex over time.

The authors must present all aforementioned analyses with appropriate figures and quantitative discussion in the Results and Discussion sections.

6. Lines 586–588 describe reference [76] as "Al-Khafaji et al. (2024) on a Staphylococcus aureus vaccine." However, Reference 76 in the bibliography (Biswas et al., 2024, Biologicals) is a study on human cytomegalovirus (CMV), not S. aureus. This is a clear citation error that was not corrected from the previous revision. All references cited in the Discussion must be verified against their actual content.

7. The authors disclosed in the response letter that 1,250 MHC-I, 1,875 MHC-II, and 980 B-cell epitopes were initially predicted, leading to a final selection of 15 (0.4% retention rate). This information is essential for evaluating the rigor of the selection process but is absent from the manuscript body. It must be incorporated into the Methods and/or Results section.

8. Roary (version 3.13.0) is a pan-genome pipeline originally developed for bacterial genomes. Its applicability to viral pan-genome analysis — particularly for RNA viruses like HMPV — has not been validated in the literature. The authors provide no justification for this methodological choice. A detailed explanation or citation of precedent studies using Roary for viral pan-genomics must be provided, or an alternative validated tool should be used.

9. Line 478 states: "For a respiratory virus like HMPV, a Th1 response is the only way to achieve viral clearance." This is an overstatement that is not supported by immunological evidence. Th2-mediated humoral responses, particularly neutralizing antibody production, also play a significant role in controlling respiratory viral infections. This sentence must be revised to reflect the current understanding of balanced Th1/Th2 immunity in HMPV clearance.

10. Reference 11 is listed as: "human-metapneumovirus-evolution-and-interaction-with-the-innate-i @ share.google. Available: https://share.google/...". A Google Share link does not constitute a citable scientific reference. This citation must be replaced with the original peer-reviewed publication.

11. The complete amino acid sequence of the lead candidate HMPV-V2 (including adjuvant, linkers, and all epitope components) is not provided in the manuscript or supplementary materials. This information is essential for reproducibility and is standard practice in immunoinformatics publications. It must be added as supplementary material.

12. The authors use ProtParam's instability index (<40) to conclude that all vaccine constructs are "stable." It should be noted that this index was developed for in vitro conditions and does not accurately predict in vivo stability under physiological conditions. The text should be qualified accordingly to avoid overclaiming.

13. A significant deficiency of the Discussion section is the absence of a comparison with directly relevant, recently published immunoinformatics studies on HMPV vaccines. The authors are required to engage with the following key studies:

Ehsasatvatan et al. (BMC Infectious Diseases, 2025)

Naji et al. (Scientific Reports, 2025)

Fardad et al. (VirusDisease, Springer, 2025)

14. Despite the authors' claim that professional language editing was performed, the following errors remain in the revised manuscript:

• Line 61: "HMPV belongs the Pneumoviridae family" → should read "HMPV belongs to the Pneumoviridae family"

• Line 65: "HMPV is now very well recognized as leading etiologic agent" → missing article: "as a leading etiologic agent"

• Line 75: "respiratory microrganisms" → spelling error: "microorganisms"

• Line 191: "screened for allergenicity, allergenicity and toxicity" → word "allergenicity" is repeated; likely should be "antigenicity, allergenicity and toxicity"

• Line 380: "immugenicity booster" → spelling error: "immunogenicity"

• Line 453: "Fig 5 also shows the complex files.Tablefiles. Table 5." → apparent copy-paste merge error: "Fig 5 also shows the complex files. Table 5."

The manuscript requires a thorough, genuine language review by a professional editor or a native English speaker with scientific background before resubmission.

**********

what does this mean?). If published, this will include your full peer review and any attached files.

If you choose “no”, your identity will remain anonymous but your review may still be made public.

**Do you want your identity to be public for this peer review?**  For information about this choice, including consent withdrawal, please see our Privacy Policy

Reviewer #4: No

Reviewer #5: No

**********

# Author comment: Author response to Decision Letter 3

**DOI:** 10.1371/journal.pone.0357758.r006

22 Aug 2026

Dear PLOS ONE Editorial Office,

We are pleased to submit the revised version of our manuscript entitled “Computational prediction of a multi-epitope Human Metapneumovirus vaccine candidate through integrated reverse vaccinology and pan-genomic approaches” (Manuscript ID: PONE-D-25-68286R2) for further consideration in PLOS ONE.

We sincerely thank the Editor and Reviewers for their valuable comments and constructive suggestions. We have carefully considered and addressed all of the comments and have made the necessary revisions to the manuscript. A detailed point-by-point response to the reviewers’ comments has also been provided with the revised submission.

We believe that the revised manuscript has been substantially improved and hope that it is now suitable for publication in PLOS ONE.

Thank you for your time and consideration. We appreciate the opportunity to revise and resubmit our manuscript and look forward to your further consideration.

Sincerely,

Md Mohaimenul Islam Tareq

Reviewer #4 Comments and Our Responses:

1. Please revise the citation formatting throughout the manuscript. In several instances where multiple references are cited to support the same statement, the references are presented in separate adjacent brackets (e.g., [1] [2] [3]). According to standard scientific writing and the journal's formatting guidelines, these citations should be consolidated into a single bracket (e.g., [1–3]). Please ensure consistency in citation formatting throughout the manuscript.

Response: We thank the reviewer for this helpful observation. We have carefully revised the citation formatting throughout the manuscript to ensure consistency with the journal's reference style. Consecutive references supporting the same statement have been consolidated into a single citation wherever appropriate, and the reference list has been updated accordingly. We believe the revised manuscript now complies with the journal's formatting requirements.

2. Authors should provide a rationale for the choice of linkers and adjuvant.

Response: We thank the reviewer for this helpful comment. The manuscript has been revised to explicitly state the rationale for selecting the AAY, GPGPG, and KK linkers, as well as the 50S ribosomal protein L7/L12 adjuvant and the EAAAK linker. While the original manuscript described the functional roles of these components, we have now clarified that they were selected based on their extensive validation in previous multi-epitope vaccine studies and their established ability to facilitate efficient antigen processing, preserve epitope integrity, minimize junctional epitope formation, enhance immunogenicity, and maintain structural separation between the adjuvant and the epitope region.

3. The authors should explain the methodology in more detail.

Response: We thank the reviewer for this valuable suggestion. We respectfully note that the methodology has already been described in detail throughout the Materials and Methods section, including the databases, software tools, selection criteria, parameters, and workflow employed at each stage of the study. We believe that these descriptions provide sufficient detail to ensure the reproducibility of the study. However, we have carefully re-examined the section to ensure that the methodological descriptions are clear and logically presented.

4. Authors should assess the solubility of the vaccine construct.

Response: We thank the reviewer for this valuable suggestion. As recommended, we evaluated the solubility of the vaccine constructs using the Protein-Sol web server. The predicted scaled solubility values for HMPV_V1, HMPV_V2, and HMPV_V3 were 0.64, 0.614, and 0.80, respectively. All values exceeded the Protein-Sol solubility threshold (0.45), indicating that the constructs are predicted to be soluble upon recombinant expression. These results have been incorporated into the manuscript and further support the favorable physicochemical properties of the designed vaccine constructs.

5. Authors should perform disulfide engineering of the vaccine structure.

Response: We appreciate the reviewer's suggestion. Disulfide engineering was performed for the refined structures of all three vaccine constructs using Disulfide by Design 2 (DbD2). Candidate residue pairs were screened based on bond energy and geometric parameters and suitable residue pairs were identified for cysteine substitution after excluding native disulfide bonds. The corresponding methodology and results have been added to the revised manuscript. Based on the overall structural validation, molecular docking, molecular dynamics simulation and disulfide engineering analyses, vaccine construct 2 remained the most promising candidate for further development.

6. Authors should predict discontinuous B-cell epitopes on the 3D structure of the vaccine.

Response: We thank the reviewer for this valuable suggestion. In the present study, B-cell epitope prediction was performed using the BCPred server, which predicts linear (continuous) B-cell epitopes based on the amino acid sequence. We acknowledge that discontinuous B-cell epitope prediction using a three-dimensional structure-based approach would provide additional insights into conformational antigenic regions. However, due to the scope of the present study and time constraints associated with the revision, this analysis has not been incorporated. We have included this as a limitation of the study and as a direction for future work.

7. Authors should perform codon optimization and in silico cloning of the vaccine construct.

Response: We thank the reviewer for this valuable suggestion. Codon optimization and in silico cloning of the selected vaccine construct (HMPV_V2) had already been performed in the original manuscript. Subsequently, the optimized sequence was successfully cloned in silico into the pET28a (+) expression vector using SnapGene software (Figure 10).

8. The authors should perform the imine response simulator in response to three doses of the vaccine.

Response: We thank the reviewer for this valuable suggestion. Immune response simulation was already performed using the C-ImmSim server and included in the original manuscript. Three doses of the selected vaccine construct were administered at simulation time steps 1, 84, and 168, corresponding to four-week intervals between immunizations. The simulation evaluated the predicted humoral and cellular immune responses, including antibody production, B-cell and T-cell populations, cytokine profiles, and immune memory. To improve clarity, we have revised the corresponding section of the manuscript and explicitly highlighted the three-dose immunization schedule and its associated immune simulation results.

9. Please provide the MM-PBSA calculations of the simulated trajectory.

Response to Reviewer - We thank the esteemed reviewer for their insightful comment regarding the absence of MM-PBSA (Molecular Mechanics Poisson-Boltzmann Surface Area) calculations in our study. We fully recognize the value of MM-PBSA as a powerful computational method for quantifying binding free energies and appreciate the opportunity to clarify our approach and future perspectives.

MM-PBSA is a widely adopted post-processing technique used in computational biophysics to estimate the binding free energy of a ligand to a receptor from molecular dynamics (MD) simulation trajectories. MM-PBSA calculations are crucial as they provide a more thermodynamically rigorous estimation of binding affinity compared to simple docking scores. We acknowledge that MM-PBSA calculations would indeed provide a more detailed and quantitative understanding of the binding affinity between our designed vaccine candidate (HMPV-V2) and the TLR4 receptor. Such an analysis would complement our current findings by offering a thermodynamic perspective on the stability of the predicted interactions, which is highly relevant for vaccine efficacy. However, Our current study represents a preliminary, pan-genomic, and immunoinformatics-driven design phase for a multi-epitope HMPV vaccine candidate. The primary objectives were:

�To identify highly conserved and immunogenic epitopes across diverse HMPV strains.

�To design and structurally validate multi-epitope vaccine constructs (HMPV-V1, HMPV-V2, HMPV-V3).

�To perform initial in silico screening for antigenicity, allergenicity, toxicity, physicochemical properties, and preliminary receptor binding (molecular docking).

�To assess the structural stability of the lead candidate (HMPV-V2) through molecular dynamics simulations (RMSD, RMSF, SSE analysis) and predict its immune response via immune simulations.

While MM-PBSA is a valuable tool, its implementation requires significant computational resources and time, particularly for generating sufficiently long and well-converged MD trajectories, and then performing the subsequent free energy calculations. Given the broad scope of our initial design and screening efforts across multiple constructs and analyses, we prioritized demonstrating the structural integrity, conformational stability, and predicted functional interactions using established and widely accepted methods for this stage of vaccine development. Our molecular docking results (indicating a strong predicted binding affinity of -969.2 for HMPV-V2 with TLR4) and robust MD simulation analyses (low RMSD of 8.213 and RMSF of 0.737 for HMPV-V2) already provide compelling evidence for a stable and energetically favorable interaction, which was sufficient for selecting our lead candidate in this initial phase.

We fully agree that MM-PBSA calculations would significantly enhance the thermodynamic characterization of HMPV-V2's interaction with TLR4. However, we also believe that our performed molecular dynamics simulations (RMSD, RMSF, SSE analysis) are sufficient to validate our findings.

10. The manuscript has spelling and grammatical errors that need to be proofread and corrected.

Response: We sincerely thank the reviewer for this valuable comment. The entire manuscript has been carefully proofread and revised to correct spelling, grammatical, typographical, and punctuation errors. We have also improved sentence structure, clarity, and readability throughout the manuscript to ensure the language meets the standards of scientific writing. We appreciate the reviewer's careful evaluation, which has helped improve the overall quality of the manuscript.

11. The authors are suggested to add insights from clinical research to which relates the study with real-world relevance.

Response: We thank the reviewer for this valuable suggestion. A paragraph has been added to the Discussion section highlighting the clinical significance of HMPV infection, the current lack of a licensed vaccine and ongoing clinical and preclinical vaccine development efforts. We have also discussed how the proposed computationally designed multi-epitope vaccine may contribute to future vaccine development while emphasizing the need for experimental validation and clinical evaluation before practical application.

12. The manuscript is very grammatically poor and a native speaker should rewrite it.

Response: We sincerely thank the reviewer for this constructive comment. We acknowledge the importance of clear and accurate scientific writing. Accordingly, the manuscript has been thoroughly revised to improve grammar, sentence structure, word choice, clarity, and overall readability. We carefully proofread the entire manuscript and corrected numerous grammatical, typographical, and stylistic issues identified during the revision process. We believe these revisions have substantially improved the quality and readability of the manuscript.

13. To enrich your study, please refer to other studies on vaccine design of other microorganisms. In this case, it is recommended that you use and cite to the following studies.

https://doi.org/10.1186/s12985-026-03099-0

https://doi.org/10.1371/journal.pone.0335147

https://doi.org/10.1186/s12985-026-03072-x

Response: We sincerely thank the reviewer for this valuable suggestion. Following the recommendation, we have carefully reviewed the suggested publications and incorporated them into the revised manuscript. The Discussion section has been expanded to include recent applications of immunoinformatics-based multi-epitope vaccine design against diverse pathogens, including Group A rotavirus, Chandipura virus, and human papillomavirus (HPV)-associated cervical cancer. These studies demonstrate the broad applicability and effectiveness of computational vaccine design approaches across both infectious and non-infectious disease models. The inclusion of these references provides a broader perspective on the current progress in immunoinformatics-driven vaccine development and further highlights the relevance of our proposed HMPV multi-epitope vaccine strategy.

Reviewer #5 Comments and Our Responses:

This manuscript addresses an important and timely topic — the development of an in silico multi-epitope vaccine candidate against HMPV. The pan-genomic approach used to identify core proteins is a methodologically interesting contribution. However, as detailed above, the manuscript suffers from several critical deficiencies that must be resolved before it can be considered for publication in PLOS ONE.

The authors are strongly encouraged to conduct a rigorous revision that addresses all points raised. The scientific potential of this work can only be realized with substantially greater methodological transparency, analytical precision, and engagement with the current literature.

Response - We are grateful for the positive recognition of our work's relevance and the novelty of our pan-genomic strategy. We acknowledge the reviewer's assessment regarding the need for greater methodological transparency, analytical precision, and engagement with the current literature. We are confident that the current manuscript already provides a solid foundation in these areas, and we are committed to enhancing these aspects further in our revision.

1.Methodological Transparency:

Our Current Approach:

We believe that our current manuscript already provides a detailed account of the methodologies employed. For instance, in the "Materials and methods" section, we explicitly state the databases used (NCBI Virus), the software and versions for pan-genome analysis (PROKKA v1.14.6, Roary v3.13.0, PRANK v.170427, Virus-mPLoc), and the specific tools and parameters for epitope prediction (IEDB, netMHCpan-4.1 EL for MHC I and II, BCpred with thresholds). We also detail the screening criteria for epitopes (antigenic, non-allergenic, non-toxic) and the rationale behind linker selection (AAY for CTL, GPGPG for HTL, KK for B-cells, EAAAK for adjuvant). Furthermore, we specify the tools for physicochemical profiling (ExPASy ProtParam), 3D structure prediction and validation (GalaxyWEB, GalaxyRefine 2, ERRAT, PROCHECK, ProSA-Web), molecular docking (ClusPro v2.0), immune simulation (C-ImmSim), molecular dynamics (Schrödinger Desmond v3.6), and codon optimization (JCat, SnapGene).

2.Analytical Precision:

The manuscript presents quantitative data and specific metrics for the evaluation of our vaccine constructs. For example, in the "Results" section, we provide: Antigenicity scores, instability index, aliphatic index, and GRAVY values for all constructs (Table 3). ProSA Z-scores, Ramachandran plot percentages (e.g., 87.7% favored for HMPV-V2), ERRAT scores, and MolProbity scores for structural validation (Table 4). Detailed docking energy scores and cluster members for HMPV-V2 with TLR4 (Table 5). Specific RMSD (8.213 Å) and RMSF (0.737) values from molecular dynamics simulations, highlighting the stability of HMPV-V2. Immune simulation results describing IgG1 dominance, memory B-cell maturation, and Th1-dominant cytokine profiles.

3. Engagement with Current Literature:

Our Current Approach: We have made a concerted effort to contextualize our work within the broader landscape of HMPV vaccine research and the field of immunoinformatics. The "Introduction" highlights the unmet gaps in HMPV vaccine research and the limitations of previous strategies. The "Discussion" section explicitly compares our multi-epitope

Attachment Submitted filename: Response to Reviewer.docx

# Peer review: Decision Letter 3

**DOI:** 10.1371/journal.pone.0357758.r007

**Authors:** Haitham Amer

1 Sep 2026

Computational prediction of a multi-epitope Human Metapneumovirus vaccine candidate through integrated reverse vaccinology and pan-genomic approaches

PONE-D-25-68286R3

Dear Dr. Islam Tareq,

We’re pleased to inform you that your manuscript has been judged scientifically suitable for publication and will be formally accepted for publication once it meets all outstanding technical requirements.

Within one week, you’ll receive an e-mail detailing the required amendments. When these have been addressed, you’ll receive a formal acceptance letter and your manuscript will be scheduled for publication.

An invoice will be generated when your article is formally accepted. Please note, if your institution has a publishing partnership with PLOS and your article meets the relevant criteria, all or part of your publication costs will be covered. Please make sure your user information is up-to-date by logging into Editorial Manager at Editorial Manager® and clicking the ‘Update My Information' link at the top of the page. For questions related to billing, please contact billing support.

If your institution or institutions have a press office, please notify them about your upcoming paper to help maximize its impact. If they’ll be preparing press materials, please inform our press team as soon as possible -- no later than 48 hours after receiving the formal acceptance. Your manuscript will remain under strict press embargo until 2 pm Eastern Time on the date of publication. For more information, please contact onepress@plos.org.

Kind regards,

Haitham Mohamed Amer, PhD

Academic Editor

PLOS One

Reviewers' comments:

Reviewer's Responses to Questions

**Comments to the Author**

Reviewer #4: All comments have been addressed

Reviewer #5: All comments have been addressed

**********

2. Is the manuscript technically sound, and do the data support the conclusions?

Reviewer #4: Yes

Reviewer #5: Yes

**********

3. Has the statistical analysis been performed appropriately and rigorously?

Reviewer #4: N/A

Reviewer #5: Yes

**********

4. Have the authors made all data underlying the findings in their manuscript fully available?

The PLOS Data policy

Reviewer #4: Yes

Reviewer #5: Yes

**********

5. Is the manuscript presented in an intelligible fashion and written in standard English?

Reviewer #4: Yes

Reviewer #5: Yes

**********

Reviewer #4: The authors have carefully and accurately incorporated all comments into the manuscript, and the article is eligible for publication in this journal.

Reviewer #5: (No Response)

**********

what does this mean?). If published, this will include your full peer review and any attached files.

If you choose “no”, your identity will remain anonymous but your review may still be made public.

**Do you want your identity to be public for this peer review?**  For information about this choice, including consent withdrawal, please see our Privacy Policy

Reviewer #4: No

Reviewer #5: No

**********

# Editor report: Acceptance letter

**DOI:** 10.1371/journal.pone.0357758.r008

**Authors:** Haitham Amer

PONE-D-25-68286R3

PLOS One

Dear Dr. Tareq,

I'm pleased to inform you that your manuscript has been deemed suitable for publication in PLOS One. Congratulations! Your manuscript is now being handed over to our production team.

At this stage, our production department will prepare your paper for publication. This includes ensuring the following:

* All references, tables, and figures are properly cited

* All relevant supporting information is included in the manuscript submission,

* There are no issues that prevent the paper from being properly typeset

You will receive further instructions from the production team, including instructions on how to review your proof when it is ready. Please keep in mind that we are working through a large volume of accepted articles, so please give us a few days to review your paper and let you know the next and final steps.

Lastly, if your institution or institutions have a press office, please let them know about your upcoming paper now to help maximize its impact. If they'll be preparing press materials, please inform our press team within the next 48 hours. Your manuscript will remain under strict press embargo until 2 pm Eastern Time on the date of publication. For more information, please contact onepress@plos.org.

You will receive an invoice from PLOS for your publication fee after your manuscript has reached the completed accept phase. If you receive an email requesting payment before acceptance or for any other service, this may be a phishing scheme. Learn how to identify phishing emails and protect your accounts at https://explore.plos.org/phishing.

If we can help with anything else, please email us at customercare@plos.org.

Thank you for submitting your work to PLOS One and supporting open access.

Kind regards,

PLOS One Editorial Office Staff

on behalf of

Dr. Haitham Mohamed Amer

Academic Editor

PLOS One
